# Genome-Wide Evolutionary Analysis of Putative Non-Specific Herbicide Resistance Genes and Compilation of Core Promoters between Monocots and Dicots

**DOI:** 10.3390/genes13071171

**Published:** 2022-06-29

**Authors:** Saket Chandra, Ramon G. Leon

**Affiliations:** 1 Department of Crop and Soil Sciences, North Carolina State University, Raleigh, NC 27695, USA; schand24@ncsu.edu; 2 Genetic Engineering and Society Center, Center for Environmental Farming Systems, North Carolina State University, Raleigh, NC 27695, USA

**Keywords:** non-target site, cytochrome-P450, monooxygenase, gluthatione, transferase, metabolism

## Abstract

Herbicides are key weed-control tools, but their repeated use across large areas has favored the evolution of herbicide resistance. Although target-site has been the most prevalent and studied type of resistance, non-target-site resistance (NTSR) is increasing. However, the genetic factors involved in NTSR are widely unknown. In this study, four gene groups encoding putative NTSR enzymes, namely, *cytochrome-P450*, *glutathione-S-transferase* (*GST*), *uridine 5′-diphospho-glucuronosyltransferase* (*UDPGT*), and *nitronate monooxygenase* (*NMO*) were analyzed. The monocot and dicot gene sequences were downloaded from publicly available databases. Phylogenetic trees revealed that most of the *CYP450* resistance-related sequences belong to *CYP81* (5), and in *GST*, most of the resistance sequences belonged to *GSTU18* (9) and *GSTF6* (8) groups. In addition, the study of upstream promoter sequences of these NTSR genes revealed stress-related *cis*-regulatory motifs, as well as eight transcription factor binding sites (TFBS) were identified. The discovered TFBS were commonly present in both monocots and dicots, and the identified motifs are known to play key roles in countering abiotic stress. Further, we predicted the 3D structure for the resistant *CYP450* and *GST* protein and identified the substrate recognition site through the homology approach. Our description of putative NTSR enzymes may be used to develop innovative weed control techniques to delay the evolution of NTSR.

## 1. Introduction

Crop-infesting weeds are a foremost cause of crop loss worldwide, posing a significant danger to food security. For the past several decades, herbicides have been the tool of choice for effective weed management. Management of weeds in modern cropping systems is imperative for maintaining high crop productivity, sustainable agri-business, and global food production. Herbicide usage, especially in large extensions of land dedicated to growing a few crops, creates a substantial selection pressure and is responsible for the evolution of herbicide-resistant weeds. As the first report of 2,4-D herbicide-resistant weeds [1], herbicide resistance has evolved in all major cropping systems due to widespread herbicide use over time, causing the strong selection of resistant alleles [2]. Herbicide resistance has been recorded in 152 broadleaf and 110 grass species affecting 70 different crops to date [3,4]. There has been abundant research on the processes that bestow herbicide resistance. Target-site herbicide resistance (TSR) and non-target-site herbicide resistance (NTSR) are two broad categories in which resistance mechanisms are grouped [5]. Target-site herbicide resistance occurs when the functional or active site of proteins (frequently enzymes) undergo structural changes as a consequence of amino acid deletion or substitution, as well as when gene overexpression or amplification causes an increase in the abundance of the target protein [6]. The NTSR mechanisms, on the other hand, encompasses all resistance biochemical processes that are independent of the binding site of the herbicide to its target protein. Generally, decreased herbicide absorption/translocation, increased herbicide detoxification rate, or sequestration to organelles where the herbicide has no herbicidal activity are common NTSR mechanisms that ultimately limit the amount of herbicide reaching the site of action [7]. *CytochromeP450*, *glutathione-S-transferase* (*GST*), *uridine 5′-diphospho-glucuronosyltransferase* (*UDPGT*), *nitronate monooxygenase* (*NMO*), esterases, transporters, and aldo-keto reductase genes are known to be involved in NTSR [8,9,10,11]. NTSR mechanisms play major roles in the evolution of cross-resistance and potentially multiple-resistance, including herbicides to which weeds have never been exposed [12]. Increased detoxification rates of herbicides due to upregulation of *GST* or *CytochromeP450* genes have been associated with gains in resistance to herbicides [10,13,14,15]. Herbicide resistance has evolved rapidly as vast and genetically diverse weed species have been exposed to recurrent selection, with herbicide programs lacking diversity in mechanisms of action.

In the last 30 years, no new herbicide mechanism of action (MOA) has been registered for commercial use [16]. To delay the evolution of weed resistance to existing herbicides, the search for new herbicide MOA is urgent, but the difficulty of finding new effective lethal target sites and the lack of a detailed understanding of the mechanisms of NTSR are limiting the development of new herbicides [17]. The conventional way of identifying an MOA was to find a herbicidal or phytotoxic agent and then use physiological and biochemical methods to describe how the agent disrupted the metabolism of the plant. In recent years, ‘omics’ methods have been used to look for new targets for herbicides. Transcriptomics, proteomics, and metabolomics are examples of ‘omics’ methods that are nowadays used for herbicide target identification. Currently, the use of genomics for weed control is lagging behind applications for controlling arthropod pests and pathogens [18,19,20]. Weed reference genomes will help understand the regulatory and structural characteristics of a wide range of weedy genes and their evolution. Genome assemblies of *Conyza canadensis* (L.), *Raphanus raphanistrum* (L.), *Echinochloa crus-galli* (L.) P. Beauv., *Thlapsi arvense* (L.), *Amaranthus palmeri* S. Watson, *Amaranthus tuberculatus* (Moq.) J. D. Sauer, and *Amaranthus hybridus* (L.) have all been recently published [21,22,23,24,25,26,27]. Beyond transcriptomics, genomics holds the potential for providing new insights into weed management. The conserved motif and regulatory proteins, such as transcription factors (TF), play a critical role in the intensity and efficacy of NTSR mechanisms [28]. However, little is known about those mechanisms in the context of herbicide detoxification metabolism, in part, because of the wide range of proteins and enzymes that might be involved. Bioinformatics tools and public transcriptome and genome databases may be used to predict regulatory elements and TF based on hundreds of independent experiments. This method has been used to mine critical components of signaling and metabolic pathways in a variety of species [9,29,30,31].

Anticipating the mechanisms and conditions that favor the evolution of herbicide resistance in weed species must be a cornerstone of the development and stewardship of herbicides. For this reason, the goals of this study were (1) to identify putative NTSR genes via genomic mining and to analyze their phylogenetic relationships, (2) to identify regulatory elements responsible for NTSR genes expression that could aid in the management of NTSR evolution in weed species and development of crops with an increased spectrum of herbicide tolerance, and (3) to predict the 3-D structure of resistant CYP450 and GST proteins by homology modeling to identify key substrate-binding residues.

## 2. Materials and Methods

### 2.1. Sequence Retrieval

cDNA, protein, and genomic DNA sequences of dicotyledonous (dicot) plants (*Arabidopsis thaliana* (L.) Heynh., *Capsicum annuum* L., *Citrus clementina* hort., *Daucus carota* var. *sativus* Hoffm., *Glycine max* (L.) Merr., *Ipomoea triloba* L., *Prunus persica* (L.) Batsch., *Vitis vinifera* L., and *Amaranthus hypochondriacus* L., and monocotyledonous (monocot) plants *Eragrostris curvula* (Schrad.) Nees, *Hordeum vulgare* L., *Oryza sativa* subsp. *japonica* S. Kato, *Setaria viridis* (L.) P. Beauv., *Sorghum bicolor* (L.) Moench, *Triticum aestivum* L., and *Zea mays* L. were downloaded from Ensembl Plants (https://plants.ensembl.org/info/data/ftp/index.html; accessed on 5 November 2021). The cDNA sequence of monocot weed species *Lolium multiflorum* Lam. was retrieved from https://zenodo.org/record/832654# (accessed on 7 November 2021). YWNH2prMIuU [32] and *Echinochloa crus-galli* (L.) P. Beauv. from http://ibi.zju.edu.cn/RiceWeedomes/Echinochloa/ (accessed on 10 November 2021) [23]. The Hidden Markov Model profile UDPGT (PF00201, NMO (PF03060), CytochromeP450 (PF00067), GST_N (PF02798) and GST_C (PF00043) were downloaded from Pfam [33] (http://pfam.xfam.org/; accessed on 5 November 2021). The resistant CYP450 and GST sequences were downloaded from NCBI (https://www.ncbi.nlm.nih.gov/; accessed on 5 November 2021).

### 2.2. Identification of UDPGT, GST, NMO, and CytochromeP450

The sequences of UDPGT, GST, NMO, and CytochromeP450 proteins were retrieved from the above-mentioned plant species using the hmmsearch program of the HMMER suite [34]. HMMER searches sequence databases for homology and performs alignment of sequences by creating a profile and further, with the help of the hmmsearch program, searches and retrieves for homologs. The HMM predicted sequences were further confirmed using blastp at e-value = 1 × 10^−10^ and used for further analysis.

### 2.3. Phylogenetic Study

The Molecular Evolutionary Genetics Analysis (MEGA-X) [35] program aligned the downloaded sequences with ClustalW (v. 2.0.12, UCD, Dublin, Ireland). This software provides flexibility and allows users to work with large datasets of protein and DNA sequences with statistical methods for phylogenetic analysis. The ClustalW provides the option for simultaneously aligning a large number of sequences at a much faster rate. Following the first protein alignment, any sequences that were small, divergent, or lengthy were deleted, and re-alignment of the remaining sequences was then performed. MEGA-X was used to do phylogenetic analysis using the maximum likelihood statistical technique with 1000 bootstrap repetitions. The phylogenetic tree was edited and visualized in the software FigTree.v1.4.4 (http://tree.bio.ed.ac.uk/software/figtree/; accessed on 12 November 2021). The software FigTree is used to visualize and edit the phylogenetic tree.

### 2.4. Gene Ontology and Kyoto Encyclopedia of Genes and Genomes Pathway Analysis

The Blast2Go program [36] was used to analyze gene ontology and Kyoto Encyclopedia of Genes and Genomes (KEGG) for pathway mapping of UDPGT, GST, NMO, and CytochromeP450 selected sequences. This software imparts the flexibility for annotating a large number of DNA and protein sequences conveniently and especially for non-model organisms. In the gene ontology, every protein sequence is characterized by molecular, biological, and cellular activities. The KEGG database was mined for retrieving biochemical pathway information and enzyme codes for each gene. The KEGG database comprises information for organism biochemical pathways and genes involved in it. The conserved motif in UDPGT, GST, NMO, and CytochromeP450 were discovered using the MEME suite (https://meme-suite.org/meme/tools/meme; accessed on 12 November 2021) with no more than 15 motifs per sequence. The MEME Suite is a comprehensive software tool for analyzing sequence motifs in DNA and protein sequences. Numerous biological activities are encoded by such motifs, and their identification and characterization are critical in studying vital cell regulatory mechanisms, including the modulation of gene expression.

### 2.5. In Silico Expression Analysis of NTSR Genes

Genevestigator (https://genevestigator.com/; accessed on 15 November 2021) was used to explore the expression of UDPGT, GST, NMO, and CytochromeP450 genes after herbicide exposure. The Genevestigator database comprises carefully curated and extensive expression profiles from 11 distinct plant species (www.genevestigator.com; accessed on 15 November 2021). This software analyzes carefully curated bulk tissue and single-cell transcriptome data from public sources for different species.

### 2.6. Identification of Cis Motifs and Transcription Factors Binding Sites in Promoters of NTSR Genes

Sequences in the promoter region were extracted up to 1 kb of NTSR genes. The promoters of NTSR genes were evaluated using the web-based program “The PlantPan 3.0 (http://plantpan.itps.ncku.edu.tw/ accessed on 15 November 2021) and New PLACE database [37] to check for the presence of transcription factors binding sites (TFBS) and motifs in *cis*-acting regulatory DNA elements in the promoter region of NTSR genes. The PlantPan and New PLACE database is a useful tool for identifying TFBs, associated TFs, and other relevant regulatory features in a plant promoter region.

### 2.7. Homology Modelling and Protein Secondary Structure Assignment

The resistant GST and CYP450 sequences were aligned as representations to better understand the structure and identify substrate binding sites. The software ESPript [38] was used to assign secondary structure elements to the matching aligned sequences. The substrate recognition site was predicted by aligning the closest matched sequence for GST, and for CYP450, the substrate binding site was identified as described by Dueholm et al. [39]. To choose the optimum templates and target for the homology model, all resistant GST and CYP sequences were submitted to the Phyre2 server [40]. Phyre2 uses homology-based alignment and builds accurate 3-D models based on similarity with the earlier reported 3-D structures. The model building technique was based on model confidence, query sequence coverage, and query sequence resemblance with the template. Further, the homology 3-D model was built using the phyre2 server. The details of the target-template used for homology modeling is provided in Table S1. To confirm the validity of the homology model, Ramachandran plot analysis in the Ramachandran plot server (https://zlab.umassmed.edu/bu/rama/; accessed on 21 November 2021) service was employed, and ERRAT (https://saves.mbi.ucla.edu/; accessed on 21 November 2021) was used to analyze the amino acid environment.

## 3. Results

### 3.1. Identification of Putative NTSR Genes

For this study, we selected four important NTSR genes, namely, *CYP450*, *GST*, *UDPGT,* and *NMO,* and mined these genes in monocot and dicot species. Furthermore, we identified a total of 1190 genes, 559 *CytochromeP450*, 534 *GST*, 73 *UDPGT*, and 24 *NMO* genes. The species-wise classification of NTSR genes is provided in Table 1. The validation of *CytochromeP450*, *GST*, *UDPGT*, and *NMO* domain was confirmed with pfam [33] (http://pfam.xfam.org/; accessed on 5 November 2021). Additionally, to have a better understanding of NTSR, we incorporated resistant CYP450s and GSTs belonging to different classes based on earlier studies (Table S2). The resistant *CYP450* and *GST* genes belonged to species *Glycine max*, *Papaver rhoeas* L., *Arabidopsis thaliana*, *Aegilops tauschii* Coss., *Alopecurus myosuroides* Huds., *Lolium rigidum* Gaudin, *Hordeum vulgare*, *Triticum aestivum*, *Oryza sativa* L., *Helianthus tuberosus* L., *Nicotiana tabacum* L., *Echinochloa phyllopogon* (Stapf) Vasc., and *Zea mays*.

### 3.2. Gene Ontology and KEGG Pathway Analysis

Furthermore, the potential gene ontology and KEGG pathways were analyzed for the extracted gene sequences. The gene ontology is a set of three terms (i.e., molecular function, biological process, and cellular component) that describe our understanding of the biological role of a gene [41]. The KEGG is a database that contains information about genomes and genes and biological pathways associated with them. The gene ontology analysis of *CytochromeP450* genes showed that they are involved in ion binding and oxidoreductase activity. The gene ontology analysis of *GST* linked this gene family to cellular nitrogen and sulfur compound metabolic processes, transferase activity, transferring alkyl or aryl (other than methyl) groups, and ion binding. Similarly, *NMO* dominant gene ontology processes were related to catalytic, oxidoreductase activity, acting on single donors with incorporation of molecular oxygen and incorporation of one atom of oxygen (internal monooxygenases or internally mixed-function oxidases). The major pathways for the identified *NMO* genes were glyoxylate metabolism and glycine degradation. Finally, *UDPGT* genes were involved in glucosyltransferase activity, glucuronidation of flavonoids, and their biosynthesis and hormonal glycosylation. The major KEGG pathways for *UDPGT* genes were glucuronidation, zeatin biosynthesis, heme degradation, biosynthesis of cofactors, steroid hormone biosynthesis, glycosphingolipid metabolism, and phenylpropanoid biosynthesis.

A motif is a small stretch of evolutionarily conserved protein, RNA, or DNA sequence, and that conservation may be related to important structural functions such as correct folding or an active site in enzymes. Sequence motifs are thus one of the most fundamental functional elements in molecular evolution. As a result, recognizing and comprehending these motifs is critical for understanding biological processes and twigging the causes of NTSR. Therefore, we used the MEME suite to find the most common and conserved motifs within all four NTSR gene families. In the CytochromeP450 protein motif, the three most common motifs are found in all analyzed sequences. In GST protein, motifs 1 and 2 were found in the majority of the sequences. Further, in NMO protein, motifs 1, 3, 4, and 5 were found in all sequences. Lastly, in UDPGT protein sequences, motifs 3, 4, 7, and 8 were present in most of the sequences. The summarized output of motif analysis for the four NTSR genes is provided in Table 2.

### 3.3. Phylogenetic Analysis

**Figure 1:**
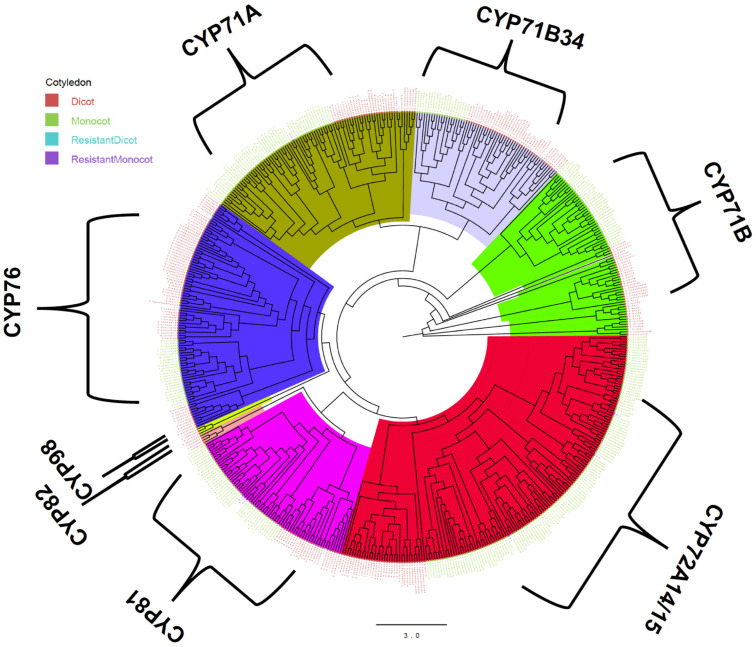
Phylogenetic tree of the *CytochromeP450* genes. The tree was constructed using the maximum likelihood statistical technique with 1000 bootstrap repetitions. The letters in red, green, violet, and blue legend represents dicot, monocot, *CYP450* resistant monocot, and *CYP450* resistant dicot. The scale bar for evolutionary distance is 3.0. Major clades are highlighted with colors: CYP76 (blue), CYP71A (dark green), CYP71B34 (light blue), CYP71B (green), CYP72A14/15 (red), CYP81 (pink), CYP82 (orange), and CYP98 (light green).

The phylogenetic tree is a pictorial representation that infers how different organisms, species, or genes are derived from a common progenitor. Phylogenetic trees are valuable for shaping information on biological variability, arranging classifications, and offering insight into evolutionary events. Here, phylogenetic analysis showed that the different putative NTSR genes were not equally distributed between monocots and dicots and were not clearly separated in different clades. Furthermore, monocot species had more and more diverse gene families than dicot species. The phylogenetic tree of CytochromeP450 further revealed that the identified sequences belong to families *CYP71A*, *CYP71B34*, *CYP71B*, *CYP72A14/15*, *CYP81*, *CYP82*, *CYP98*, and *CYP76*, and the largest clade belonged to the group *CYP72A14/15* (Figure 1). Most of the resistant genes were grouped within *CYP81* (5), followed by *CYP72A14/15* (Figure 1).

**Figure 2:**
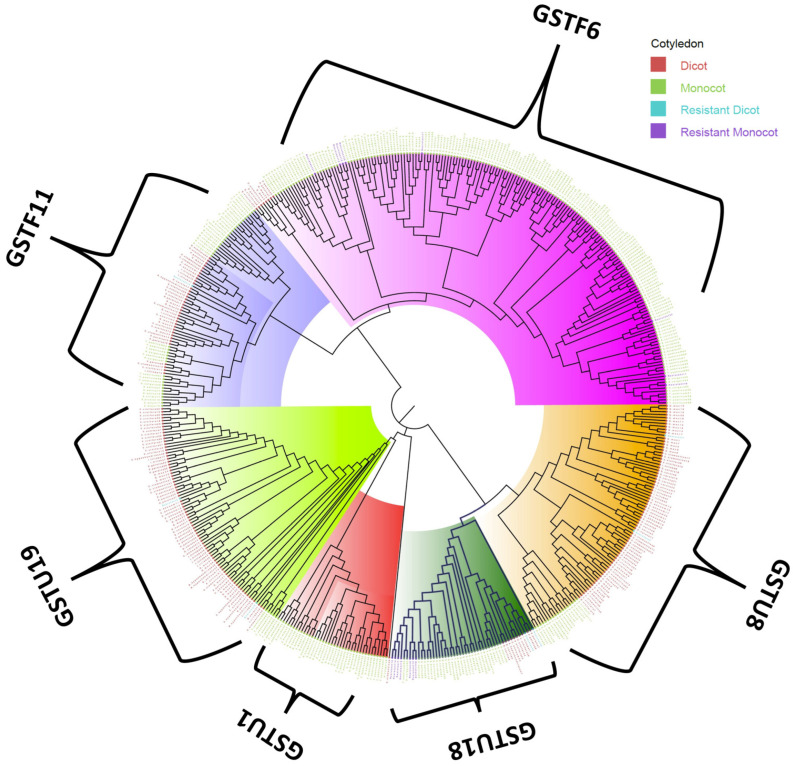
Phylogenetic tree of gene *GST*. The tree was constructed using the maximum likelihood statistical technique with 1000 bootstrap repetitions. The letters in red, green, violet, and blue legend represents dicot, monocot, *GST* resistant monocot, and *GST* resistant dicot. The scale bar for evolutionary distance is 3.0. Major clades are highlighted with colors: GSTU19 (light green), GSTF11 (light blue), GSTF6 (pink), GSTU8 (orange), GSTU18 (dark green), and GSTU1 (red).

GSTs have a wide range of substrate specificity, and lone genes can impart resistance against various herbicides. Based on the phylogenetic analysis, we found that GSTs were categorized into *GSTF6*, *GSTF11*, *GSTU1*, *GSTU8*, *GSTU18*, and *GSTU19* (Figure 2). The majority of the resistant genes belonged to *GSTU18* (9) and *GSTF6* (8) groups.

**Figure 3:**
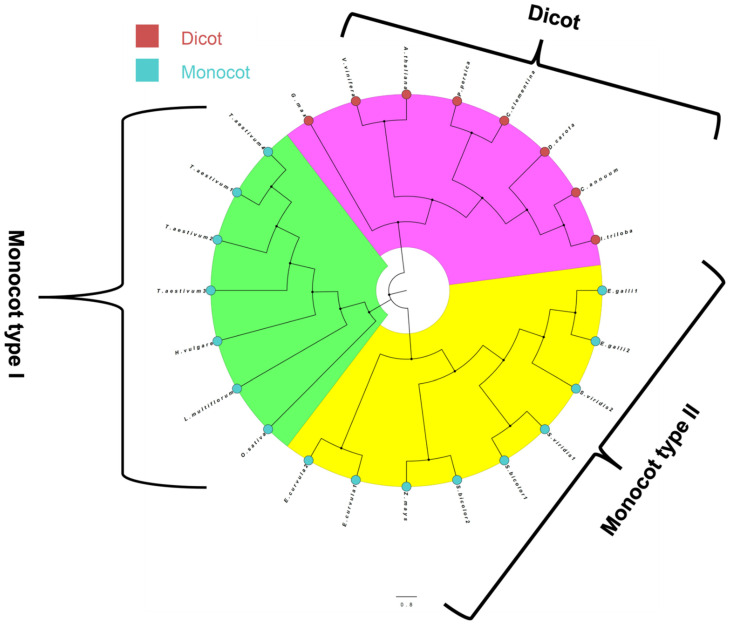
Phylogenetic tree of gene NMO. The tree was constructed using the maximum likelihood statistical technique with 1000 bootstrap repetitions. The circular nodes in red and blue represent dicot and monocot species. The scale bar for evolutionary distance is 0.8. The NMO is broadly divided into three clades; the dicot clade is highlighted with pink color, whereas monocot type I and monocot type II are highlighted with green and yellow, respectively.

**Figure 4:**
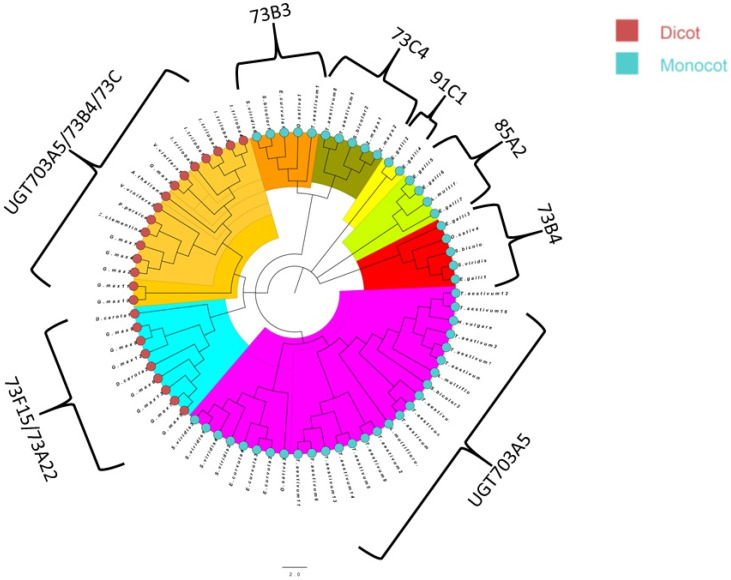
Phylogenetic tree of gene *UDPGT*. The tree was constructed using the maximum likelihood statistical technique with 1000 bootstrap repetitions. The circular nodes in red and blue represent dicot and monocot species. The scale bar for evolutionary distance is 2.0. Major clades are highlighted with colors: UGT703A5/73B4/73C (light orange), 73B3 (dark orange), 73C4 (green), 91C1 (yellow), 85A2 (light green), 73B4 (red), UGT703A5 (pink), and 73F15/73A22 (blue).

The phylogenetic tree of NMO was broadly categorized into dicot and monocot, where monocot is further subdivided into two groups (Figure 3). Finally, the UDPGT phylogenetic tree was sub-categorized into *UGT703A5*, *UGT73B4*, *UGT85A2*, *UGT91C1*, *UGT73C4*, *UGT73B3*, *UGT703A5/73B4/73C*, and *UGT73F15/73A22*. The maximum number of sequences belonged to group *UGT703A5* in UDPGT phylogenetic tree (Figure 4).

### 3.4. In-Silico Expression Analysis

**Figure 5:**
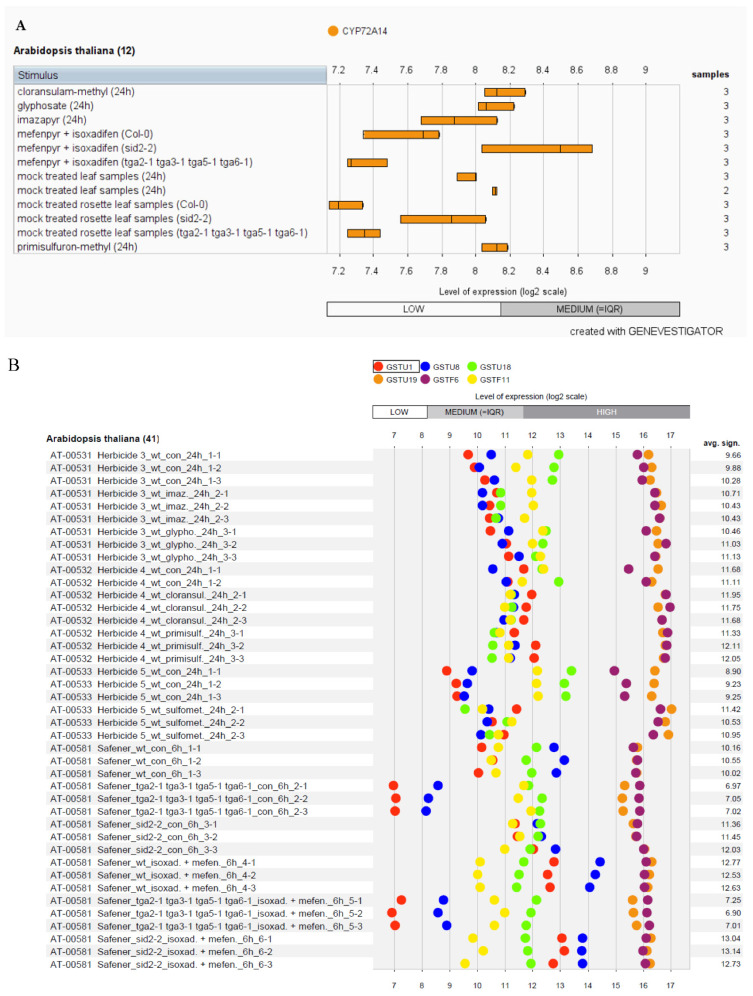

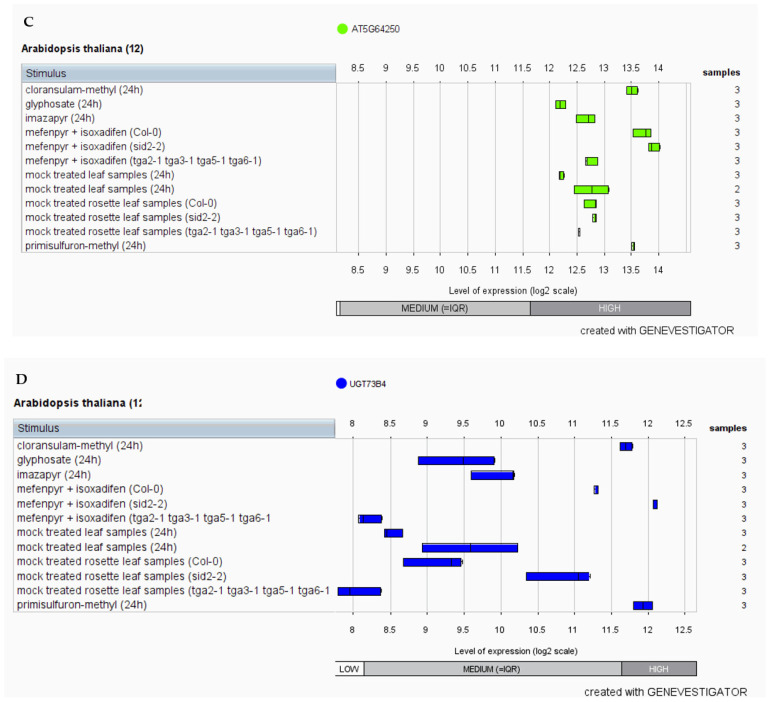
The expression profile of *CYP72A14* (**A**), *GSTU1*, *GSTU8*, *GSTU18*, *GSTU19*, *GSTF6*, and *GSTF11* (**B**), *NMO* gene *AT5G64250* (**C**), *UDPGT* gene *UGT73B4* (**D**) under herbicide stress using the software Genevestigator. The level of expression is provided in the log2 scale. The details of herbicide used, rate of applications, and NCBI Accession no. are provided in Supplementary Table S3.

We used a publicly accessible gene expression database (Genevestigator) to investigate the differential expression of putative NTSR genes in *Arabidopsis thaliana* (L.) Heynh. in response to applying different herbicides [42,43,44,45,46] (Table S3). The cytochromeP450 gene expression (*CYP72A14*) was increased in response to cloransulam-methyl, glyphosate, and mefenpyr + isoxadifen applications (Figure 5A). The expression of *GSTF6* and *GSTU19* were higher when compared with *GSTU1*, *GSTU8*, *GSTU18*, and *GSTF11* (Figure 5B). Further, NMO, *AT5G64250* gene expression was increased due to cloransulam-methyl, glyphosate, mefenpyr+isoxadifen, and primisulfuron-methyl (Figure 5C), while UDPGT gene, *UGT73B4*, was upregulated by cloransulam-methyl, imazapyr, mefenpyr + isoxadifen and primisulfuron-methyl (Figure 5D).

**Figure 6:**
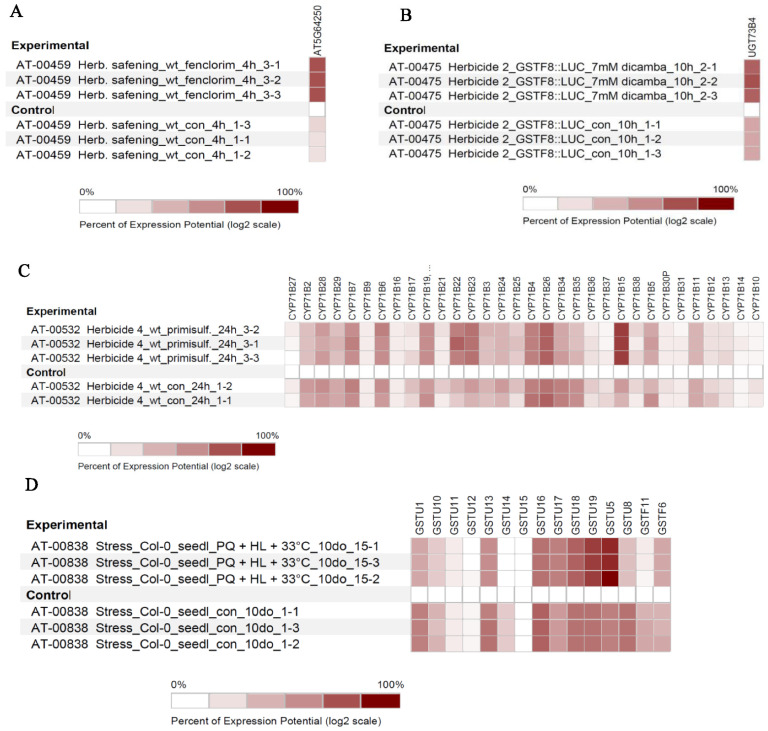
Heatmap-list view of the expression profile of *NTSR* gene under herbicide stress using the Genevestigator software *NMO* affymetrix Arabidopsis expression analysis with response to herbicide safener fenclorim (**A**), *UGT73B4* affymetrix Arabidopsis expression increases proportionally in the presence of herbicide dicamba when compared with control (**B**), *CYP45071* sub-family affymetrix Arabidopsis expression study due to the presence of herbicide primisulfuron (sulfonylurea) (**C**), and *GST* affymetrix Arabidopsis expression study when plants were exposed to herbicide paraquat (**D**). The level of expression is provided in the log2 scale. The details of the experiments are provided in Supplementary Table S3.

**Figure 7:**
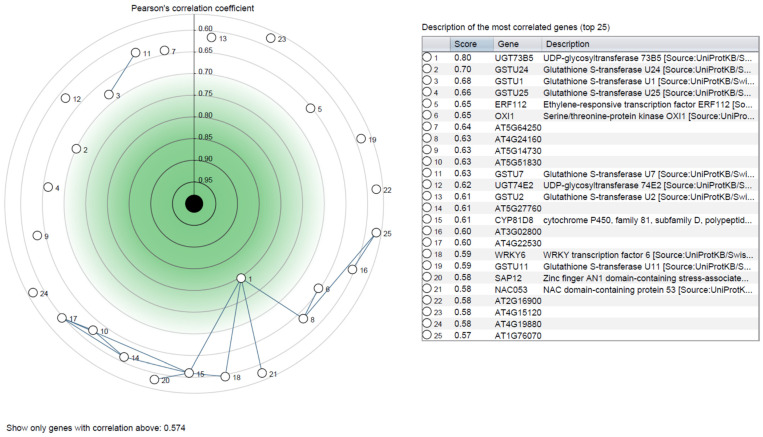
Co-expression analysis for the NTSR genes in response to herbicide stress. The top 25 co-expressed genes are represented in the circular plot. There is a cluster of genes that are mutually connected. The mutual correlation score will make it possible to see links between genes that are mutually associated. The top 25 co-expressed genes are listed in the adjacent table. The details of the experiments are provided in Supplementary Table S3.

Additionally, we compared the expression of four NTSR genes with control samples individually. The expression of NMO genes was upregulated in response to safener fenclorim when compared with the control samples (Figure 6A). Similarly, the UDPGT gene expression was increased after exposure to dicamba (Figure 6B). Furthermore, in response to primisulfuron-methyl, we found the expression of *CYP71B15* was highest (Figure 6C). The heatmap expression analysis of GST revealed that the expression of *GSTU19* and *GSTU5* were highest with paraquat when compared with control samples (Figure 6D). Lastly, Pearson’s correlation coefficient was used to identify the top 25 most upregulated and correlated genes in response to herbicide exposure (Figure 7).

### 3.5. Identification of Cis-Motifs and TFBS in Promoters

Deciphering the architecture of the NTSR response is complicated by the co-occurrence of single to many NTSR proteins and regulatory factors, which seem to interact through convergent signal transduction pathways, especially in resistant weeds. This resistance is caused by the regulators and TF that act in the promoter region, increasing the expression of NTSR genes, but the level and efficacy of the resistance are dependent on the specificity of the signal transduction in response to the herbicide and the appropriate control of the NTSR genes [9,47]. Sequence-dependent secondary characteristics of promoters are undoubtedly significant in their function, according to experimental findings. Although the genomes of several weeds have been sequenced, we still lack high-quality assembled reference genomes. Therefore, we could not extract the 1 kb promoter region of many weed species, so the analysis included more cultivated species.

The most prevalent motif in CytochromeP450, GST, NMO, and UDPGT is provided in Table 3 (Supplementary Files S1–S4). The occurrence of motifs remains approximately the same in both monocot and dicot species. Comparing the TFBS of monocot and dicot, Dof, Myb/SANT; MYB; ARR-B, and AT-hook (a small DNA binding domain) are found mostly in dicots and the rest of the binding motifs are present in monocots and dicots at similar frequencies. The TFBS in GST upstream region in both dicot and monocot are mostly the same, except for the presence of α-amylase in dicots. The motifs between monocot and dicot species in NMO are almost the same. The TF C_2_H_2_ is mostly found in diploid species when compared with UDPGT upstream sequences of monocot species.

### 3.6. Homology Modelling of Resistant CYP450 and GST Proteins

We conducted a homology modeling approach to unravel the 3D structure of resistant CYP450 and GST protein using Phyre2. This analysis helped model the protein sequence with the top-scoring template (Table S1). The Ramachandran plot for the corresponding protein revealed that most of the residues fall in the allowed regions. Additionally, the 3D model was also verified with the ERRAT scores indicating an adequate protein environment. The resistant CYP450 3D structure homology modeled is provided in Supplementary File S5. Despite their minimal amino acid sequence similarity, organisms appear to adopt a 3D structure that has remained consistent during evolution when compared with the template. Furthermore, according to Gotoh’s hypothesized models, we found six probable substrate recognition and binding sites for CYP450 (Supplementary File S6). For GST proteins, based on alignment, we modeled the 3D structure of 22 resistant sequences (Supplementary File S7). Despite the low overall commonality across the sequences of diverse GST classes, 3D structures of GST were discovered to have a 3D similar structure [48].

## 4. Discussion

Resistance to herbicides is a form of evolution and a well-known instance of dynamic responses against selection pressure applied by humans [49,50]. Because herbicide resistance is becoming more common worldwide, a greater knowledge of resistance evolution and the mechanisms that underpin resistance are critical for the development of better weed management techniques. However, because plants have enzymes with different substrate specificities, this makes gene identification for NTSR an arduous task [51]. In this study, we selected four important gene groups (*CYP450*, *GST*, *UDPGT*, and *NMO*) which have been reported to confer NTSR [9,52]. Our goal was to analyze the genes related to herbicide resistance; therefore, we included only the plant-specific GSTs reputed to have a very critical role in herbicide detoxification [53,54,55]. GSTs defend plants against oxidative damage by catalyzing the conjugation of glutathione with different herbicide classes [53]. Another enzyme, CYP450, plays a critical role in herbicide detoxification in major weeds [56]. Also, a variety of plants have been reported to have herbicide detoxifying CYP450s [57]. For example, CYP450s can detoxify herbicides such as ALS- and ACCase-inhibitors either by dealkylation or hydroxylation [58]. Also, cytochromeP450 is responsible for the detoxification of noxious chemicals by facilitating NADPH and oxygen-dependent mono-oxygenation steps that help in transforming herbicides into water-soluble, herbicidal-inactive metabolites [59,60]. UDPGTs, in addition to GSTs, have been implicated in herbicide detoxification based on conjugation with proteins [61,62]. Phytohormones, secondary metabolites, and xenobiotics are among the lipophilic small molecule acceptors conjugated by UDPGT proteins [63]. UDPGT can metabolize different organic molecules such as the herbicide pinoxaden and the explosive 2,4,6–trinitrotoluene [64,65]. In the case of UDPGT, herbicide detoxification is achieved by glucuronidation-mediated removal of lipophilic xenobiotics by aiding their transfer to the vacuole for further degradation [66]. Although glycosylation is a crucial phase for herbicide resistance in plants due to an intrinsically high level of glycosyltransferase activity, its role in herbicide tolerance in weeds is unknown. Nevertheless, multiple insect UDPGT have been found to contribute to pesticide resistance in various species by being part of the detoxification metabolism [67]. In *Alopecurus myosuroides* Huds., increased UDPGT action, in combination with GSTs and CytochromeP450, detoxifies several herbicides, namely dichlormid, cloquintocet mexyl, aryloxyphenoxypropionate, phenylurea, and sulphonylurea [68]. Most reports of herbicide resistance with the involvement of UDPGT genes are based on RNA-seq research for weeds resistant to ALS- and ACCase-inhibitors [66]. In addition, nitrochemicals are abundantly found throughout the environment and are regularly used in herbicides [69], and NMO enzymes are involved in the catabolism of these nitrochemicals [70,71]. Similarly, NMO genes have been related to enhanced metabolism-mediated herbicide resistance in weeds [11,61].

The gene ontology and KEGG pathway analysis indicated that all identified *CytochromeP450* genes are involved in pathways related to the metabolism of xenobiotics by cytochrome P450 and glutathione conjugation [72,73]. It is worth mentioning that CytochromeP450 contributes to about 1% of the plant genome, which highlights the scale and importance of this gene family as part of regulating pathways for a diverse range of stress responses, including herbicide detoxification in plants [51]. *GST* genes are involved in a variety of processes, including plant stress responses, development, and xenobiotic detoxification. Our findings revealed that most *GST* genes were involved in a variety of catalytic tasks. Both gene ontology and KEGG analyses provided robust evidence that the retrieved sequences are conserved and coincide with other GSTs [74,75].

In the phylogenetic analysis, most of the resistant *CYP450* belonged to *CYP81* and *CYP72A14/15* groups. *CYP81* has been reported to play an important role in resistance to bensulfuron-methyl in *Echinochloa phyllopogon* by detoxification through O-demethylation [76]. The CYP81 has also been associated with resistance to pendimethalin, fenoxaprop-P-ethyl, and other ACCase- and ALS-inhibitors [14,76,77]. Furthermore, the expression of *CYP72A15* increases in *Echinochloa colona* after florpyrauxifen-benzyl, quinclorac, and propanil applications, suggesting involvement in NTST [78,79]. *CYP72A15* is involved in hydroxylation reactions to reduce herbicides for further breakdown [52]. The tau and phi classes are the most prevalent in plants and critical factors in the removal of xenobiotics such as herbicides and industrial pollutants from the environment [80]. For the phylogenetic analysis of the GST gene, our sequence belonged to the tau and phi classes, which have distinct substrate specificity. Herbicides such as thiocarbamate and chloroacetanilide are strongly reactive to phi-class enzymes [81]. The phi class has high glutathione peroxidase activity that aids plants in gaining resistance to different classes of herbicides [55,82]. On the other hand, aryloxyphenoxypropionate and diphenylether herbicides are effectively countered by tau enzymes [81]. Tau-class GSTs have important roles in safener-induced responses [45,80]. The majority of the genes found in the present study belonged to the tau class.

UDPGT is the largest class of glycosyltransferases present in the plant kingdom [83]. The phylogenetic analysis of *UDPGT* genes indicated a considerable expansion of UDPGTs among monocot and dicot plant species spread across eight groups. Interestingly, *UGT703A5* monocot and dicot fall in different clades, indicating structural differences between them. Although there is little protein similarity among the *UDPGT* families, the protein structure appears to be conserved similarly to an earlier report [84]. Lastly, in NMO phylogenetic analysis, we found that monocots and dicots are clearly grouped into different clades, and monocot NMOs were further subdivided into two groups. NMOs belong to class H of the favin-dependent monooxygenases. They are divided into two classes, class I and class II, based on structural differences, substrate specificity, and catalytic efficiencies [71].

In the gene expression analysis, we found that all four NTSR genes correlated in response to herbicide resistance, especially *UGT73B5*. These findings clearly indicate that the expression of these putative NTSR genes is part of signal transduction pathways that identify the presence and/or action of the herbicides and differentiate them from mock applications, increasing the potential for herbicide detoxification. The upregulation of these genes has been reported in several studies in dicots and monocots [52,58,61,65,85,86].

The bendability, curvature, and stability of DNA in these promoter regions are three important qualities that are frequently associated with the TFBS and *cis*-regulatory motifs [87]. Therefore, to find common and important motifs and TF in monocot and dicot species, we implemented a detailed in silico analysis on 20 monocot and dicot species, including some weeds, which used two plant-specific nucleotide databases, New PLACE and PlantPan. The New PLACE database is a collection of motifs for plant cis-acting regulatory DNA elements gathered from published findings. Furthermore, the PlantPan database is a useful tool for discovering TFBSs, associated TFs, and additional significant regulatory features (tandem repeats and CpG islands) in a plant promoter or group of promoters. Identifying regulatory *cis*-elements in promoter regions is critical for understanding the temporal and spatial expression patterns of genes engaged in a certain function. These genes might be controlled by groups of TFs with similar properties and DNA sequence affinities, which may be identified by the presence of certain regulatory *cis*-motifs in the promoter. Therefore, we examined the promoter regions of NTSR genes and identified the topmost motifs and TFBS present in the studied monocot and dicot species.

Considering all four NTSR gene types, TFs: AP2/ERF, B3, Dof, NF-YB, TCP, Trihelix, ZF-HD, and GATA:tify were discovered to be mutual in dicot and monocot species, while six conserved motifs: ARR1AT, CAATBOX1, CACTFTPPCA1, DOFCOREZM, GT1 CONSENSUS, and GTGANTG10, were found to be common in the promoter region of monocot and dicot species. The AP2/ERF family is one of the TF families implicated in plant responses to abiotic stress and is part of various regulatory and developmental pathways [88,89,90]. The B3 TF is part of stress signaling and disease resistance [91]. The Dof TF cloned from rice and expressed in *Escherichia coli* gained the characteristic to survive in drought as well as saline stress [92]. The TF NF-YB has an important role in improving the antioxidant ability and drought stress tolerance in plants [93], while TCP TFs are important growth regulators that transduce a variety of environmental and endogenous cues that are suited to guarantee plant growth and development [94]. Some TFs can act in a tissue-specific manner, such as Trihelix TF, which has roles not only in biotic stress but also in perianth, stomata, and trichome development, late embryogenesis, and abscission layer of seeds [95]. In addition, the GATA:tify TF has been found to be upregulated in response to imazamox, suggesting a potential role in herbicide resistance [96] (Wright 2017), while the ZF-HD TF is upregulated in abiotic stress against xenobiotics [9,97].

The distribution of *cis*-elements identified in all NTSR gene promoters are involved in TF modulation and expression of herbicide detoxification genes. Therefore, looking into the conserved motifs common between monocots and dicots, we found that all six motifs are related to stress tolerance [98,99,100,101,102,103,104,105].

In resistant CYP450s, six probable substrate recognition site areas were identified in this study based on homology. This type of recognition site plays an important role in determining substrate specificity [39] and consequent substrate transformation [106,107,108]. For example, in the proline-rich membrane pivot found in both animal and plant CYP450, the amino acid surrounding cysteine in substrate recognition site 4, which forms an axial ligand for a heme called “I-helix,” binds oxygen and the Glu–Arg–Arg trio utilizing the glutamic acid and arginine residues of the K-helix (ExxR) and the arginine in the “PERF” conserved region [39]. The Glu–Arg–Arg trio is assumed to be crucial in securing the heme site into place and ensuring the stability of the core structure [39]. We also modeled 22 resistant GST proteins. GSTs are 50-kDa homodimers with an α-helical C-terminal domain and a thioredoxin-folded N-terminal domain which are joined by a linker [109]. GST has two ligand-binding sites in each subunit, with the N-terminal exhibiting a conserved glutathione binding site known as G-site and the C-terminal containing a variable H-site that can bind to xenobiotic compounds [110]. In this study, we identified both the G-site and the H-site for the herbicide-resistant proteins.

## 5. Conclusions

Herbicide resistance is one of the most significant challenges to agricultural production, and it has a worldwide impact. It is vital to know how plants cope with stress to maintain growth. Therefore, a thorough analysis is required to identify and understand the regulatory mechanisms related to herbicide stress that will help researchers discover and alter important regulatory elements to delay or mitigate problems associated with herbicide-resistant weeds. Since the start of agriculture, TF and *cis*-regulatory motifs have played a significant role in crop development. Because of their position as master regulators of gene clusters, TFs and *cis*-regulatory motifs are attractive candidates for genetic engineering to increase herbicide resistance in crops. In this study, we extracted the NTSR genes and performed phylogenetic, gene ontology, in-silico expression analysis, homology modeling, identification of key residues, and explored the promoter region to identify the TFBS and *cis*-regulatory motifs. In total, we found eight TFBS and six *cis*-regulatory motifs common to all NTSR genes. All of these TF and *cis*-motifs have been reported to play important roles in mitigating the potential negative effects of numerous abiotic stresses. Importantly, the fact that TF and *cis*-regulatory motifs are common to all NTSR genes studied here suggests that selection might act on a single or few TFs and not directly on the NTSR genes. This TF selection could raise the expression of NTSR genes, ultimately favoring enough herbicide detoxification to achieve resistance to label rates by the additive or synergistic action of multiple NTSR proteins. For this reason, the study of TFs, as well as key regulatory motifs, must be a core component of NTSR research.

## Figures and Tables

**Table 1:** Species-wise identification of non-specific herbicide resistance genes.

Species	Functional Group	NMO	UDPGT	Cytochrome P450	GST
*Amaranthus hypochondriacus*	Dicot	0	0	5	8
*Arabidopsis thaliana*	Dicot	1	1	0	0
*Capsicum annuum*	Dicot	1	0	39	24
*Citrus clementina*	Dicot	1	1	48	21
*Daucus carota*	Dicot	1	2	26	23
*Glycine max*	Dicot	1	13	47	39
*Ipomoea triloba*	Dicot	1	5	36	20
*Prunus persica*	Dicot	1	1	17	31
*Vitis vinifera*	Dicot	1	2	22	38
*Echinochloa crus-galli*	Monocot	2	7	0	0
*Eragrostris curvula*	Monocot	2	4	23	30
*Hordeum vulgare*	Monocot	1	1	71	51
*Lolium multiflorum*	Monocot	1	3	0	0
*Oryza sativa*	Monocot	1	3	39	43
*Setaria viridis*	Monocot	2	6	23	22
*Sorghum bicolor*	Monocot	2	4	30	28
*Triticum aestivum*	Monocot	4	18	99	115
*Zea mays*	Monocot	1	2	22	19

**Table 2:** Most predominant motif distribution in NTSR genes.

Gene		Most Common Motif
*CYP450*	Motif 1	GDBFEFIPFGAGRRICPGQNFAL
	Motif 2	IKAECKDLFFAGTETTSVTLEWAM
	Motif 3	YLTMIIKETLRLHPPAPLLLP
*GST*	Motif 1	TYYFMATPYASLFDAYPHVKAWWEDJMARP
	Motif 2	GEHKSPEHLARNPFGQVPALQD
*NMO*	Motif 1	CLGTRFVATEESFAHPLYKRKLIEMSCTDYTBVFGRARWPGAPQRVLETP
	Motif 3	DHVRELIRKTRSLTEKPFGAAIVLAFPHEENLRVVLEEKLAVLQVYWGEF
	Motif 4	DGIIVQGREAGGHVIGQEGLLPLLPRVVDLVSDSGIPVIAAGGIVDGRGY
	Motif 5	GILGFDYGIVQAPLGPDISGPELAAAVANAGAIGLLRLPDW
*UDPGT*	Motif 3	PLHILFFPFLAPGHLIPLADMA
	Motif 4	SYGEVFNSFHELEPDYAEHYRT
	Motif 7	RAKELGEKARAAVEEGGSSYNDVGRLIDE
	Motif 8	CTIJTTPVNAAVIRSAVDRAN

**Table 3:** List of dominant motifs and TFBS present in all four NTSR genes.

NTSR Gene	Motif	TFBS
*CYP450*	ABRELATERD1, IDE1 element, SURECOREATSULTR11, IBOXCORE, TAAGSTKST1, ASF1MOTIFCAMV, WBOXHVISO1, BIHD1OS, WBOXATNPR1 CCAATBOX1, DOFCOREZM, WRKY71OS, CACTFTPPCA1, GT1CONSENSUS, GTGANTG10, ARR1AT	GATA; tify, AP2; ERF, Dof, ZF-HD, Homeodomain; TALE, B3, NF-YB, TCP, Trihelix, dehydrin
*GST*	CAATBOX1, CCAATBOX1, IBOXCORE, LTRECOREATCOR15, CGACGOSAMY3, MYBST1, SORLIP1AT, BIHD1OS, GT1CONSENSUS, DOFCOREZM, GTGANTG10, WRKY71OS, CACTFTPPCA1, ARR1AT, EBOXBNNAPA, MYBCOREATCYCB1, ABRELATERD1	GATA; tify, Dof, AP2; ERF, B3, bHLH, ZF-HD, NF-YB, Trihelix, TCP, Myb/SANT; MYB; ARR-B, SBP, WRKY, bZIP
*NMO*	CAATBOX1, GATABOX, EBOXBNNAPA, GT1CONSENSUS, POLLEN1LELAT52, DOFCOREZM, GTGANTG10, TAAAGSTKST1, MYCCONSENSUSAT, WRKY71OS, CACTFTPPCA1, ARR1AT	GATA; tify, Dof, ZF-HD, Homeodomain; TALE, Myb/SANT; MYB; ARR-B, AP2; ERF, B3, NF-YB, Dehydrin, TCP, Trihelix, bZIP, bHLH, SBP
*UDPGT*	CAATBOX1, GATABOX, DOFCOREZM, WRKY71OS, CACTFTPPCA1, ARR1AT	GATA; tify, Dof, Homeodomain;TALE, AP2; ERF, B3, NF-YB, Dehydrin, TCP, Trihelix, bZIP

## Data Availability

Not applicable.

## Supplementary Materials

The following supporting information can be downloaded at: https://www.mdpi.com/article/10.3390/genes13071171/s1, Table S1: Template and target protein used for homology modeling; Table S2: List of resistant CYP450 and GST used for this study; Table S3: Information on the differential expression study used for this study; Supplementary Files S1–S4: Motifs sequences; Supplementary File S5: Homology modeled 3D structure of Resistant CYP450 genes; Supplementary File S6: Substrate recognition site identified in Resistant CYP450 protein sequence; Supplementary File S7: Homology modeled 3D structure of Resistant GST genes; Supplementary File S8: Sequence showing the GSH binding site (G-site) and substrate binding pocket site (H-site) in the resistant GST sequence.
