# Supplementary material for: Genome-Wide Evolutionary Analysis of Putative Non-Specific Herbicide Resistance Genes and Compilation of Core Promoters between Monocots and Dicots
Source: Genes (Basel). 2022 Jun 29;13(7):1171. doi: 10.3390/genes13071171 (PMC9316059; doi:10.3390/genes13071171)
Supplement: Supplementary file 1 [file genes-13-01171-s001.zip › Supplementary file S5.pdf]

**Supplementary file S5: Homology modelled 3D structure of Resistant CYP450 genes**

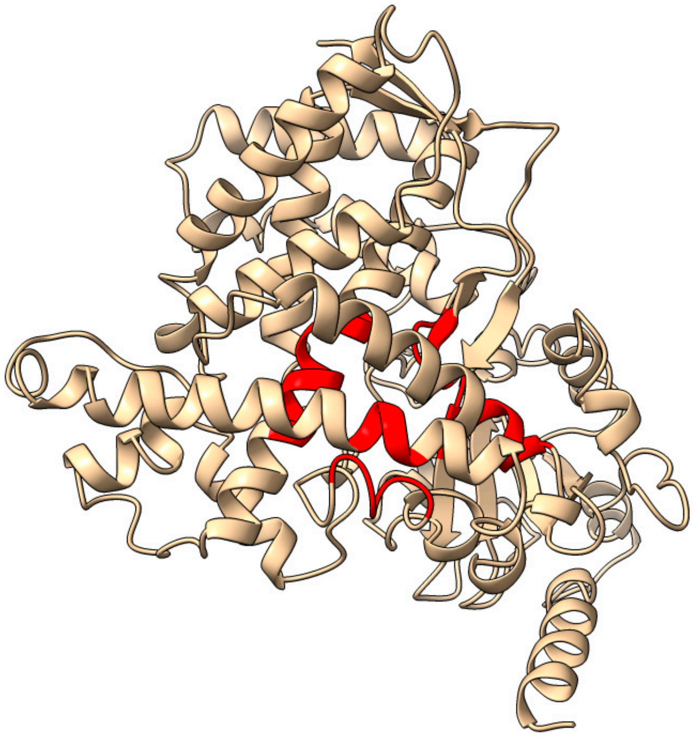

Figure 1: 3D homology modelling of CYP450 resistant gene R1. The Substrate recognition site is highlighted in Red

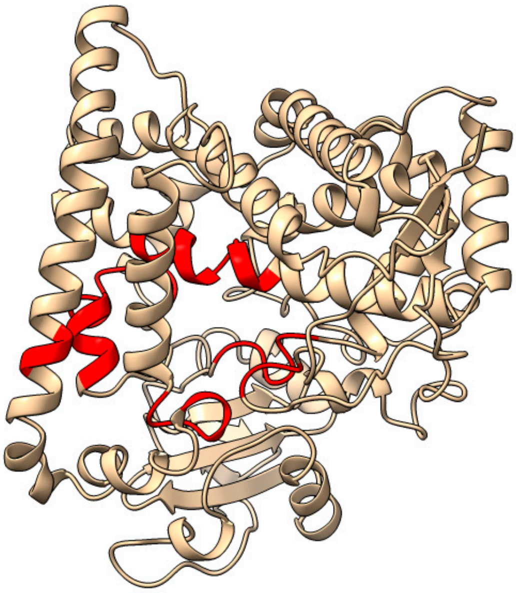

Figure 2: 3D homology modelling of CYP450 resistant gene R2. The Substrate recognition site is highlighted in Red

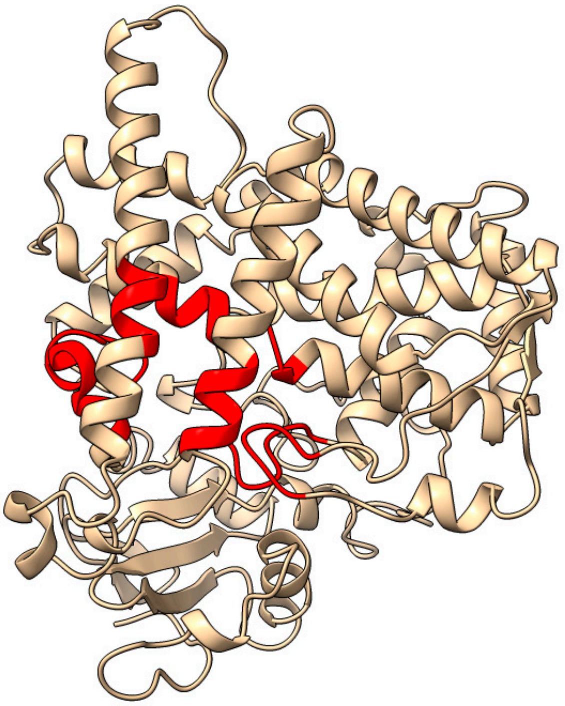

Figure 3: 3D homology modelling of CYP450 resistant gene R3. The Substrate recognition site is highlighted in Red

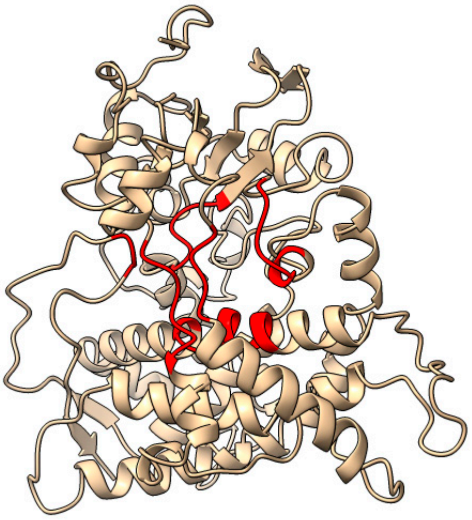

Figure 4: 3D homology modelling of CYP450 resistant gene R4. The Substrate recognition site is highlighted in Red

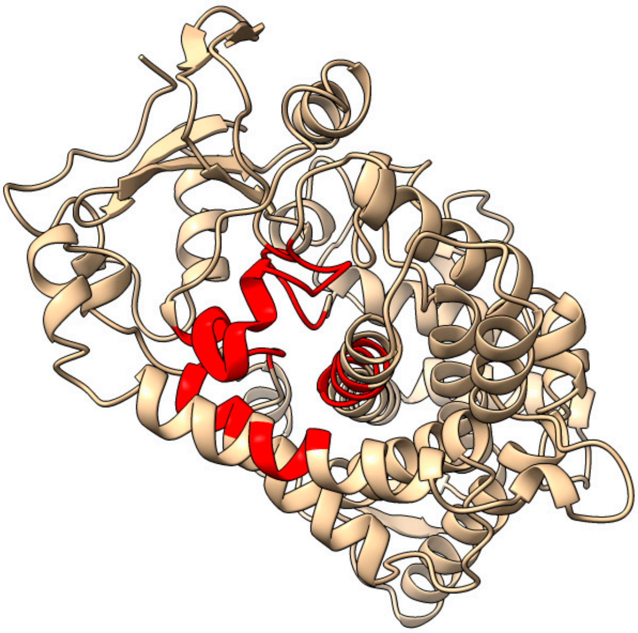

Figure 5: 3D homology modelling of CYP450 resistant gene R5. The Substrate recognition site is highlighted in Red

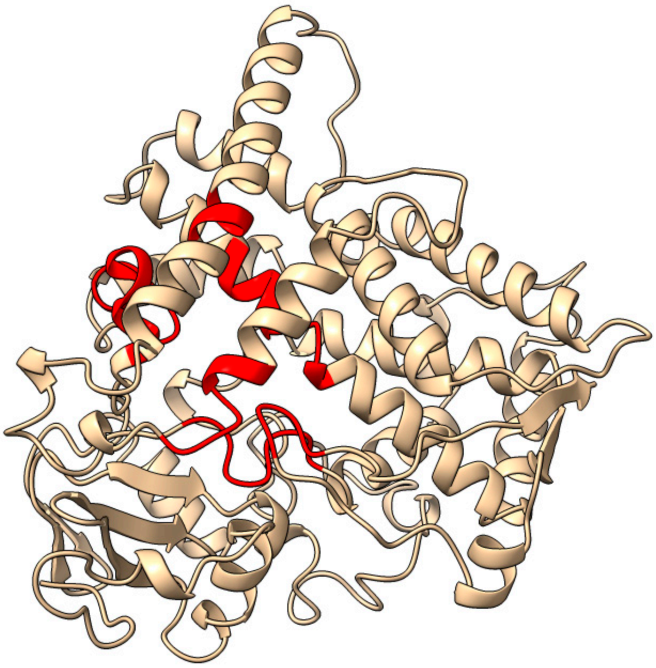

Figure 6: 3D homology modelling of CYP450 resistant gene R6. The Substrate recognition site is highlighted in Red

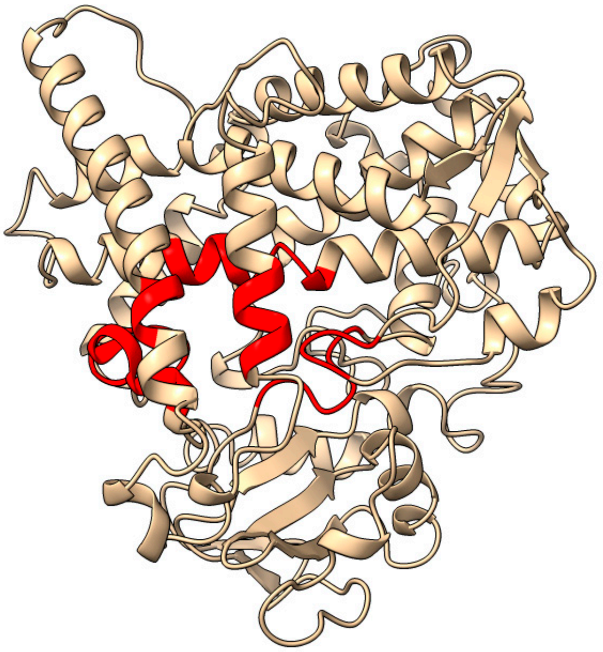

Figure 7: 3D homology modelling of CYP450 resistant gene R7. The Substrate recognition site is highlighted in Red

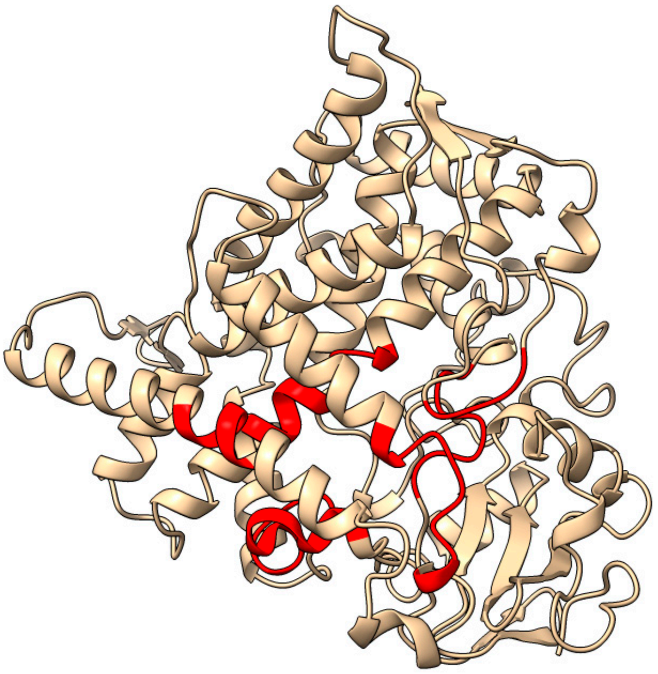

Figure 8: 3D homology modelling of CYP450 resistant gene R8. The Substrate recognition site is highlighted in Red

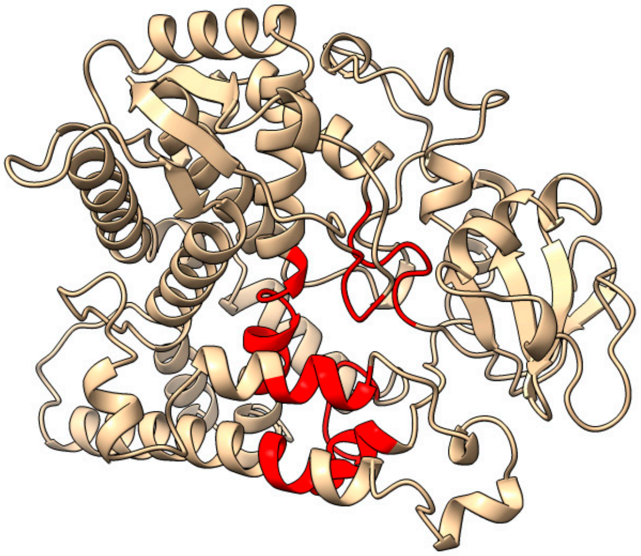

Figure 9: 3D homology modelling of CYP450 resistant gene R9. The Substrate recognition site is highlighted in Red

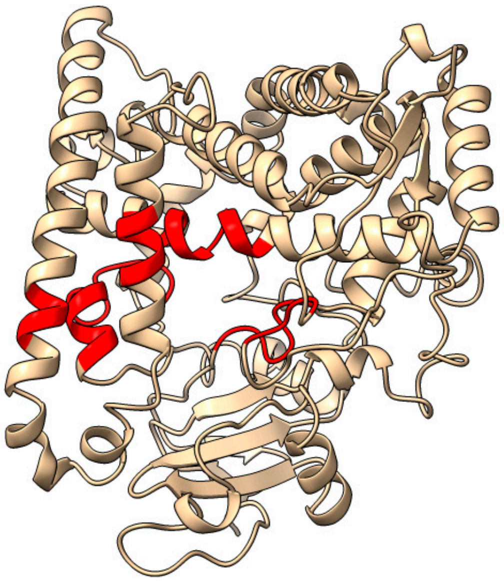

Figure 10: 3D homology modelling of CYP450 resistant gene R10. The Substrate recognition site is highlighted in Red

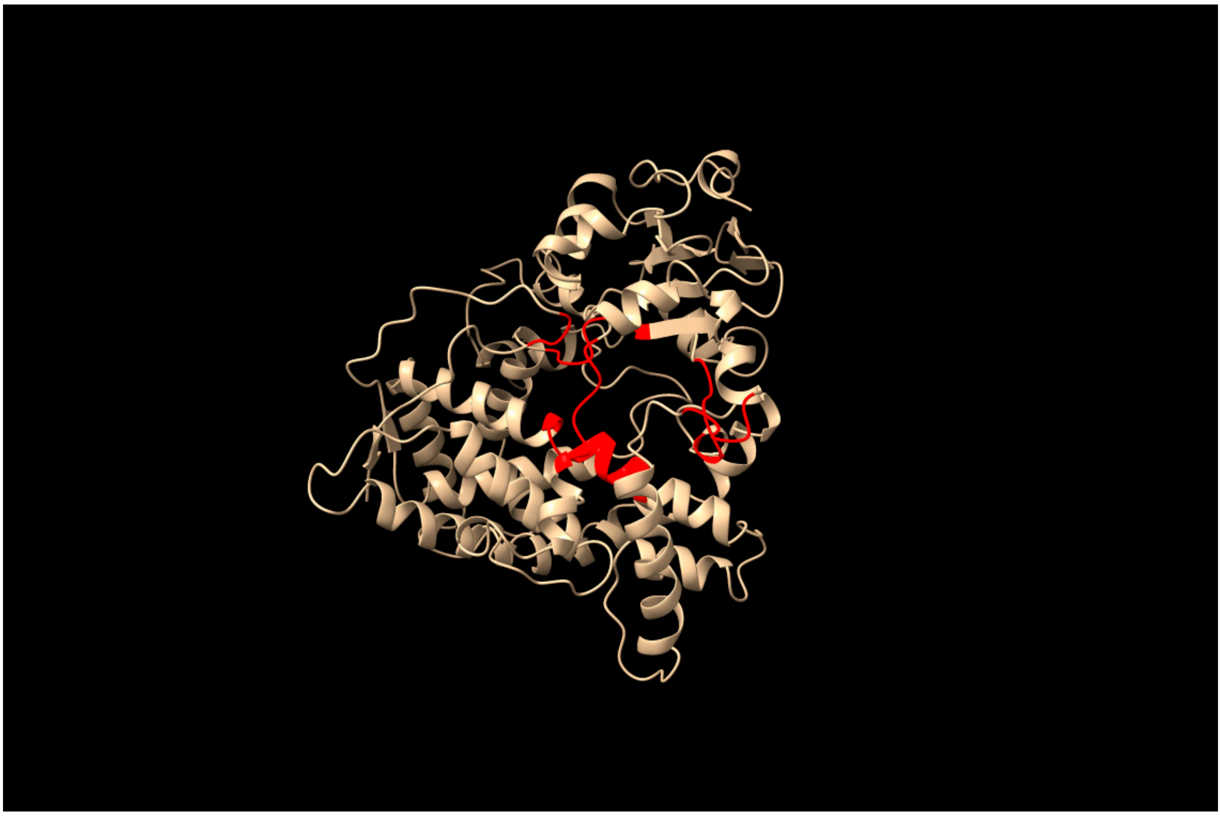

Figure 11: 3D homology modelling of CYP450 resistant gene R11. The Substrate recognition site is highlighted in Red

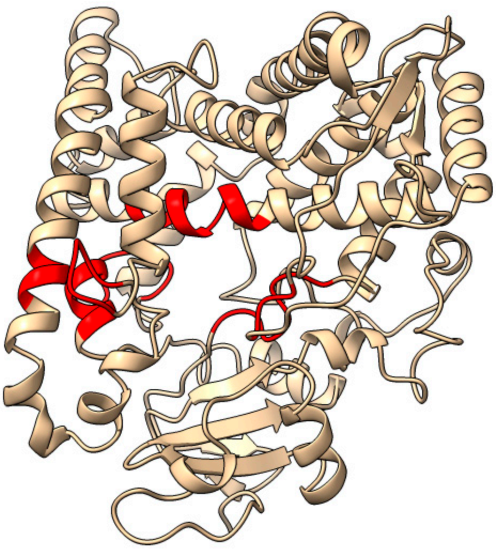

Figure 12: 3D homology modelling of CYP450 resistant gene R12. The Substrate recognition site is highlighted in Red
