# Supplementary material for: Genome-Wide Evolutionary Analysis of Putative Non-Specific Herbicide Resistance Genes and Compilation of Core Promoters between Monocots and Dicots
Source: Genes (Basel). 2022 Jun 29;13(7):1171. doi: 10.3390/genes13071171 (PMC9316059; doi:10.3390/genes13071171)
Supplement: Supplementary file 1 [file genes-13-01171-s001.zip › Supplementary file S6.pdf]

Supplementary file S6: Substrate recognition site identified in Resistant CYP450 protein sequence. The sequence highlighted in yellow is substrate recognition site

```
R1             ------------------------------------------------------------ 0
R4             ------------------------------------------------------------ 0
R11            ------------------------------------------------------------ 0
R8             ------------------------------------------------------------ 0
R2             ------------------------------------------------------------ 0
R9             MAFLGWAVDIARDSGASSSVVLTCDGYGSALYFSPWDSVPLPATASPDDGFLLPRFPDVC 60
R12            ------------------------------------------------------------ 0
R3             ------------------------------------------------------------ 0
R5             ------------------------------------------------------------ 0
R10            ------------------------------------------------------------ 0
R6             ------------------------------------------------------------ 0
R7             ------------------------------------------------------------ 0
CYP71AJ13      ------------------------------------------------------------ 0
CYP71AJ14      ------------------------------------------------------------ 0
CYP71AJ12      ------------------------------------------------------------ 0
CYP71AJ11      ------------------------------------------------------------ 0
CYP71AJ9       ------------------------------------------------------------ 0
CYP71AJ8       ------------------------------------------------------------ 0
CYP71AJ7       ------------------------------------------------------------ 0
CYP71AJ15      ------------------------------------------------------------ 0
CYP71AJ6       ------------------------------------------------------------ 0
CYP71AJ5       ------------------------------------------------------------ 0
CYP71AJ4       ------------------------------------------------------------ 0
CYP71AJ21      ------------------------------------------------------------ 0
CYP71AJ25      ------------------------------------------------------------ 0
CYP71AJ3       ------------------------------------------------------------ 0
CYP71AJ1       ------------------------------------------------------------ 0
CYP71AJ2       ------------------------------------------------------------ 0


R1             ------------------------------------------------------------ 0
R4             ------------------------------------------------------------ 0
R11            ------------------------------------------------------------ 0
R8             ------------------------------------------------------------ 0
R2             ------------------------------------------------------------ 0
R9             VQRSQFTNHLAPANGTGGGGSRTGVKEEASEVLSWPPTSKQSVRRLEVAEHWYRLYKTDN 120
R12            ------------------------------------------------------------ 0
R3             ------------------------------------------------------------ 0
R5             ------------------------------------------------------------ 0
R10            ------------------------------------------------------------ 0
R6             ------------------------------------------------------------ 0
R7             ------------------------------------------------------------ 0
CYP71AJ13      ------------------------------------------------------------ 0
CYP71AJ14      ------------------------------------------------------------ 0
CYP71AJ12      ------------------------------------------------------------ 0
CYP71AJ11      ------------------------------------------------------------ 0
CYP71AJ9       ------------------------------------------------------------ 0
CYP71AJ8       ------------------------------------------------------------ 0
CYP71AJ7       ------------------------------------------------------------ 0
CYP71AJ15      ------------------------------------------------------------ 0
CYP71AJ6       ------------------------------------------------------------ 0
CYP71AJ5       ------------------------------------------------------------ 0
CYP71AJ4       ------------------------------------------------------------ 0
CYP71AJ21      ------------------------------------------------------------ 0
CYP71AJ25      ------------------------------------------------------------ 0
CYP71AJ3       ------------------------------------------------------------ 0
CYP71AJ1       ------------------------------------------------------------ 0
CYP71AJ2       ------------------------------------------------------------ 0


R1             ------------------------------------------------------------ 0
R4             ------------------------------------------------------------ 0
R11            ------------------------------------------------------------ 0
R8             ------------------------------------------------------------ 0
R2             ------------------------------------------------------------ 0
R9             QRLSPDSQQVSVLAESHCDLASGNWKEISIHHKKMPSSTTTKTTTPSRDAWIVSARSDPF 180
```

```
R12             ------------------------------------------------------------ 0
R3              ------------------------------------------------------------ 0
R5              ------------------------------------------------------------ 0
R10             ------------------------------------------------------------ 0
R6              ------------------------------------------------------------ 0
R7              ------------------------------------------------------------ 0
CYP71AJ13       ------------------------------------------------------------ 0
CYP71AJ14       ------------------------------------------------------------ 0
CYP71AJ12       ------------------------------------------------------------ 0
CYP71AJ11       ------------------------------------------------------------ 0
CYP71AJ9        ------------------------------------------------------------ 0
CYP71AJ8        ------------------------------------------------------------ 0
CYP71AJ7        ------------------------------------------------------------ 0
CYP71AJ15       ------------------------------------------------------------ 0
CYP71AJ6        ------------------------------------------------------------ 0
CYP71AJ5        ------------------------------------------------------------ 0
CYP71AJ4        ------------------------------------------------------------ 0
CYP71AJ21       ------------------------------------------------------------ 0
CYP71AJ25       ------------------------------------------------------------ 0
CYP71AJ3        ------------------------------------------------------------ 0
CYP71AJ1        ------------------------------------------------------------ 0
CYP71AJ2        ------------------------------------------------------------ 0


R1              --------------------------------MATR-ALQMLGEASPWNLACA--AAAMA 25
R4              --------------------------------MVFRGWLMWAPASAP-VLVVFGLLFGLA 27
R11             --------------------------------MA-LGALATEFASTPWSFLIYGLLGALL 27
R8              --------------------------------MENL------NSSYYYYYYYLAI----- 17
R2              ----------------------------------------------MDKAY-IAI----- 8
R9              HLLLEAQAPLGIKADALSQIAAVHQSHRNTSHIREL------SLA-MDNAYIIAI----- 228
R12             ----------------------------------------------MDNAYIIAI----- 9
R3              --------------------------------MDII------SGQA---LLLL------- 12
R5              --------------------------------MDF--------------LIIV------- 7
R10             ---------------------------------------------MSPYLLAAAAA---A 12
R6              --------------------------------MALLSS---VLKQLPHEL--SST----- 18
R7              ----------------------------------------------MKFLLVVAS----- 9
CYP71AJ13       --------------------------------MIL-------EQQP-LFLSICSL----- 15
CYP71AJ14       --------------------------------MIL-------DQQF-LFLSLCSL----- 15
CYP71AJ12       --------------------------------MIL-------DQQL-LFLSLCSL----- 15
CYP71AJ11       ----------------------------------M-------DQQSL-FLCLCFL----- 13
CYP71AJ9        --------------------------------MVL-------DQQSLFLLSLCSL----- 16
CYP71AJ8        ----------------------------------M-------DQQSL-FLSLCSL----- 13
CYP71AJ7        ---------------------------------MM-------DQQAL-FLSLSLM----- 14
CYP71AJ15       ---------------------------------MM-------DQQAL-FLSLCSM----- 14
CYP71AJ6        ---------------------------------MM-------DQQTL-FLSLCSM----- 14
CYP71AJ5        --------------------------------MMM-------DQQTL-FLSLCSM----- 15
CYP71AJ4        --------------------------------------------------YFFSL----- 5
CYP71AJ21       --------------------------------MKM-------VEQYPLYLYFFSL----- 16
CYP71AJ25       --------------------------------MKM-------LEQYHLYVYFFSL----- 16
CYP71AJ3        --------------------------------------------------YFFPL----- 5
CYP71AJ1        --------------------------------MKM-------LEQNPQYLYFFSL----- 16
CYP71AJ2        --------------------------------------------------YFFSL----- 5


R1              VLWLAAWILEWAWWTPRRLGRALEAQGLKGTRYRLFTGDVPENARLNKEARSKPLPLGSH 85
R4              LVWQAGRLLHRLWWRPRRLEKALRARGLRGSSYRFLTGDLAEESRRRKEAWARPLPLRCH 87
R11             LWKGAARLLEPLWWAPRRLERALRAQGLRGTSYRFLTGDLKEYGRANKEAWSRPLPLRRH 87
R8              LLFFFVILFKYLL----------------PSGKR-----------LPPSPLSLPIIGHLY 50
R2              LSCAFLFLVHYVLGKVSHGRRG------KKGAVQ-----------LPPSPPAIPFIGHLH 51
R9              LSVAILFLLHYYLL-----GRG------NGGAAR-----------LPPGPPAVPILGHLH 266
R12             LSVAILFLLHYYLL-----GRG------NGGAAR-----------LPPGPPAVPILGHLH 47
R3              ---FCFILSCFLIFTTTRSGRI------SRGATA-----------LPPGPPRLPIIGNIH 52
R5              ---STLLLSYILIWVL-G----------VGKPKN-----------LPPGPTRLPIIGNLH 42
R10             AGVLIVFL---Y---VVK----------NRRRGK-----------LPPSPPSMPLIGHLH 45
R6              -HYLTVFFCIFLILLQLI----------RRNKYN-----------LPPSPPKIPIIGNLH 56
R7              -LFLFVFL---ILSATKR----------KSKAKK-----------LPPGPRKLPVIGNLL 44
CYP71AJ13       -LFVLVFL---YLWLSTS----------RTTGKN-----------LPPSPPKLPIIGNLH 50
CYP71AJ14       -FFVLVFL---YIWLSTT----------KTTGKN-----------VPPSPRKLPIIGNLH 50
CYP71AJ12       -FFVLVFL---YIWLSTT----------KTTGKN-----------VPPSPRKLPIIGNLH 50
CYP71AJ11       -FFVLVFL---YIWLSTS----------KTTGKN-----------LPPSPRKLPIIGNLH 48
CYP71AJ9        -FFVLVFL---YIWLSTS----------KPTGKN-----------LPPSPRKLPIIGNLH 51
```

```
CYP71AJ8            -FFVLVFL---YIWLSTS----------KPTGKN-----------LPPSPRKLPIIGNLH 48
CYP71AJ7            -FFVLAFL---YIWLSAS----------KTSSKN-----------LPPSPGKLPIIGNLH 49
CYP71AJ15           -FFVLGFL---YIWLSTS----------KTTGKN-----------LPPSPQKLPIIGNLH 49
CYP71AJ6            -LSVLVFL---YIWLSTS----------KTTGKN-----------LPPSPQKLPIIGNLH 49
CYP71AJ5            -FFVLVFL---YIWLSTS----------KTTGKN-----------LPPSPQKLPIIGNLH 50
CYP71AJ4            -FLVTIFL---YKWLA-K----------KTPSKN-----------LPPSPPRLPIIGNLH 39
CYP71AJ21           -LSATIFF---YKWLTLK----------KTALRN-----------LPPSPPRFPVIGNLH 51
CYP71AJ25           -ISATIFL---YKWLTLR----------KTALKN-----------LPPSPPTFPIIGNLH 51
CYP71AJ3            -FLVTIFL---YKWLV-K----------KTPSKN-----------LPPSPPRLPIIGNLH 39
CYP71AJ1            -FLVTIFL---YKWLTLK----------KTPLKN-----------LPPSPPQYPIIGNLH 51
CYP71AJ2            -FLVTVFV---YKLLTLK----------KTPSKN-----------LPPSPPRYPIIGNLH 40
                           .                                              *.

R1                  DIIPRVQPMISNAIKENGK---LSFTWFGPEPRVTILDPESVREILSNKFGHY-GKPRSS 141
R4                  DIAPRIKPFLHDTLGEHGKQRQPCITWFGPTPEVNITDPELAKVVLSNKFGHL-ERVRFK 146
R11                 DIAAYVAPFICAAVREHGK---TCFTWFGPIPKVTITDPDLARDVMSNKFGHF-EKPKFP 143
R8                  LIKNSLHETLTSLSTKY---GPVLYLRFGCRNLLVVSSPSAMEECFTKNDIIFANRPQSM 107
R2                  LVEKPIHATMCRLAARL---GPVFSLRLGSRRAVVVSSSECARECFTEHDVTFANRPKFP 108
R9                  LVKKPMHATMSRLAERY---GPVFSLRLGSRRAVVVSSPGCARECFTEHDVTFANRPRFE 323
R12                 LVKKPMHATMSRLAERY---GPVFSLRLGSRRAVVVSSPGCARECFTEHDVTFANRPRFE 104
R3                  LVGKHPHRSFAELSKTY---GPVMSLKLGSLNTVVIASPEAAREVLRTHDQILSARSPTN 109
R5                  LLGALPHQSLAKLAKIH---GPIMSLQLGQITTLVISSATAAEEVLKKQDLAFSTRNVPD 99
R10                 LIGRLAHRSLHDLQLRHGGGGGLLFLQLGRRRTLVVSTAAAAADLFRNHDLAFASRPRSV 105
R6                  QLGTLPHRSFHALSHKY---GPLMMLQLGQIPTLVVSSADVAREIIKTHDVVFSNRRQPT 113
R7                  QIGKLPHRSLQKLSNEY---GDFIFLQLGSVPTVVVFSAGIAREIFRTQDLVFSGRPALY 101
CYP71AJ13           QVNQDPHVALRSLAQKY---GPVMQLHFGSVPVLVVSSADAAKEIMKTHDLAFANRPDSS 107
CYP71AJ14           LVNQDPHVALRSLAQKY---GPFMLLHLGSVPVLVVSSADAAKEIMKTHDLAFANRPVSS 107
CYP71AJ12           QVNQDPHVALRSLAQKY---GPFMLLHFGSVPVLVVSSADAAKEIMKTHDLAFANRPDSS 107
CYP71AJ11           QVNQDPHISLRSLAKRY---GPVMQLHFGSVPVLVVSSADAAKEIMKTHDLAFANRPNSS 105
CYP71AJ9            QVNKDPHISLRSLAKRY---GQIMQLHFGSVPVLVVSSADAAKEIMKTHDLAFANRPNSS 108
CYP71AJ8            QVNKDPHISLRSLAKRY---GPLMQLHFGSVPVLVVSSADAAKEIMKTHDLAFANRPNSS 105
CYP71AJ7            LVNQDPHIALRSLAKKY---GYVMQLQFGSVPVLVVSSADAAKEVMKTHDLAFANRPNSS 106
CYP71AJ15           QVNQDPHISLRSLAKKY---GPVMQLQFGSIPVLVVSSADAAKEIMKTHDLAFANRPNSS 106
CYP71AJ6            QVNQDPHISLRSLAKKY---GPVMQLHFGSIPVLVVSSADAAKEIMKTHDLAFANRPNSS 106
CYP71AJ5            QVNQDPHISLRSLAKKY---GPVMQLHFGSIPVLVVSSADAAMEIMKTHDLAFANRPNSS 107
CYP71AJ4            QIGPDLHISLRDLARKY---GPLMQLQLGRIPVLVVSSAEATREVLKTHDVVFSQRPITS 96
CYP71AJ21           QVGPDPYISLRTLAEKY---GPLMLLKFGSVPVVVVSSAEAAREILKTHDLVFADRPFLS 108
CYP71AJ25           QIGPDPHISLRALAEKF---GPLMLLKFGSVPVLVVSSADAAREILKTHDLVFSDRPISS 108
CYP71AJ3            QIGPDPQISLRDLAREY---GPVMHLKFGSVPVLVVSSADGAREIFKTHDLVFADRPYSS 96
CYP71AJ1            QIGPDPQASLRDLAQKY---GPLMFLKFGTVPVLVVSSADAAREALKTHDLVFADRPYSS 108
CYP71AJ2            QIGPDPQHSLRDLALKY---GPLMSLKFGTVPVLVVSSADAAREVLKTHDLIFADRPYSS 97
                     :       :                 :*    : :         :  :      :

R1                  RFGKLLANGLV----NHQGEKWAKHRRILNPAFHHEKIKRMLPVFSACSEEMITRWENSM 197
R4                  EVSKLLSQGLT----YHEGEKWVKHRRIINPAFQLEKLKLMLPAFSACCEELISRWIGSI 202
R11                 ALSKLFADGVA----NYEGEKWVKHRRILNPAFHLEKLKLMLPAFSACCEELVSRWAQSL 199
R8                  AGDQFSFNYKAVVWAP-YGYLWRALRRLTVIEIFSSN---SLQKSSALRNEEIGILIRSL 163
R2                  SQLLASFNGTALVTSS-YGPHWRNLRRVATVQLLSAH---RVACMSGVIAAEVRAMARRL 164
R9                  SQLLVSFNGAALATAS-YGAHWRNLRRIVAVQLLSAH---RVGLMSGLIAGEVRAMVRRM 379
R12                 SQLLVSFNGAALATAS-YGAHWRNLRRIVAVQLLSAH---RVGLMSGLIAGEVRAMVRRM 160
R3                  AVRSINHQDASLVWLPSSSARWRLLRRLSVTQLLSPQ---RIEATKALRMNKVKELVSFI 166
R5                  AVRAYNHERHSISFLH-VCTEWRTLRRIVSSNIFSNS---SLEAKQHLRSKKVEELIAYC 155
R10                 AGERLMYGCKNITFAP-YGDDWRRGKKIAVVHLLSPR---RVESFAPVRAAEVAALVART 161
R6                  AAKIFGYGCKDVAFVY-YREEWRQKIKTCKVELMSLK---KVRLFHSIRQEVVTELVEAI 169
R7                  AGKRFSYNCCNVSFAP-YGNYWREARKILVLELLSTK---RVQSFEAIRDEEVSSLVQII 157
CYP71AJ13           IWSRIFYNGKDVAFAP-YTEYWRQVKSICILQLLSNK---RVRSFHNVREEEVGLLVENI 163
CYP71AJ14           IWSRIFYNGKDVAFAP-YTEYWRQVKSICVLQLLSNK---RVRSFYNVREEEVGLLVENI 163
CYP71AJ12           IWSRIFYNGKDVAFAP-YTEYWRQVKSICVLQLLSNK---RVRSFYNVREEEVGLLVENI 163
CYP71AJ11           IWDGIFYNGKDVVFAP-YSEYWRQVKSICVLQLLSNK---RVRSFQIVREEEVALLVEKI 161
CYP71AJ9            IWDRIFYKGKDVVFAP-YSEYWRQVKSICVLQLLSNK---RVRSFQAVREEEVALLVENI 164
CYP71AJ8            IWDSIFYKGKDVVFAP-YSEYWRQVKSICVLQLLSNK---RVRSFQTVREEEVALLVENI 161
CYP71AJ7            IWDRIFYNGKDVVFAP-YSEYWRQVKSICVLQLLSNK---RVRSFQTVREEEVALLVENI 162
CYP71AJ15           IWDRIFYNGKDVVFAP-YSEYWRQVKSICVLQLLSNK---RVRSFQTVREEEVALLVENI 162
CYP71AJ6            IWDKIFYNGKDVVFAP-YSEYWRQVKSICVLQLLSNK---RVRSFQTVREEEVALLVENI 162
CYP71AJ5            IWDKIFYNGKDVVFAP-YSEYWRQVKSICVLQLLSNK---RVRSFQTVREEEVALLVENV 163
CYP71AJ4            AIDKLCYKGRDVAFSR-YSEYWRQVRSTCVTQLLSNS---RVHSFHNIREEEVALLIQNI 152
CYP71AJ21           VANRIFYKGRDVAFAR-YSEYWRQVKSMCVTQLLSSR---RVQSFHNVREEEVALLIQNI 164
CYP71AJ25           VANRLFYNGRDVAFAL-YSEYWRQVKSMCVTQLLSSR---RVHSFHNVREEEVALLIRNI 164
CYP71AJ3            VANRIFYNGRDMVFAR-YTEYWRQVKSTCVTQLLSVK---RVQSFHNVREEEVALLLDNI 152
CYP71AJ1            VANKIFYNGKDMVFAR-YTEYWRQVKSICVTQLLSNK---RVNSFHYVREEEVDLLVQNL 164
CYP71AJ2            VANKVFYNGKDMVFAR-YTEYWRQVKSICVTQLLSNK---RVNSFQNVREEEVDLLVQNI 153
```

```
                                     *          :        :          :

R1              ---S---SQGVSEVDVWPEFQNLTGDVISRTAFGSSYQEGTKIF--------QLQGEQAE 243
R4              ---G---CDGSYEVDCWPELKSLTGDVISRTAFGSSYLEGRRVF--------ELQAEQFE 248
R11             ---G---PDGCCELDVEPELQTLTGDVISRTAFGSSYLEGRKIF--------QLQAEQAE 245
R8              FKASTNNGSSGARVNLSHWVFTFAVNVMMRTGTGKRCVS-EE--DMETEKGKQIIEEIRG 220
R2              FHAAEASPDGAARVQLKRRLFELSLSVLMETIAQTKATRSEADADTDMSVEAQEFKEVVD 224
R9              YRAAAASPAGAARIQLKRRLFEVSLSVLMETIAHTKATRPETDPDTDMSVEAQEFKQVVD 439
R12             YRAAAASPAGAARIQLKRRLFEVSLSVLMETIAHTKATRPETDPDTDMSVEAQEFKQVVD 220
R3              SESS---DR-EESVDISRVAFITTLNIISNILFSVDLGSYNAK------ASINGVQDTVI 216
R5              RKAA---LS-NENVHIGRAAFRTSLNLLSNTIFSKDLTDPYED------SG-KEFREVIT 204
R10             RRAA----EAGEAVELREFLYGYTNAVVTRAATGAAGA------------TAEKLKQLMG 205
R6              GEAC---GSERPCVNLTEMLMAASNDIVSRCVLGRKCDDACGG------SGSSSFAALGR 220
R7              ---C---SSLSSPVNISTLALSLANNVVCRVAFGKGSDEGGND------YGERKFHEILF 205
CYP71AJ13       ---R---NSGSEIVNLSDLFYTLLSNVVSRIALGKKYTNTTEG------GEENSFRELFQ 211
CYP71AJ14       ---K---DSGSNIVNLSDLTYTLLSNVVSRIALGKRYTNSTED------GEENSFRELFQ 211
CYP71AJ12       ---K---DSGSNIVNLSDLTYTLLSNVVSRIALGKKYTNSTED------GEENSFRELFQ 211
CYP71AJ11       ---K---ESGSKPVNLSELFYALLSNVVSRIALGRKYGITTEG------GKDNSFKELFQ 209
CYP71AJ9        ---K---ESGSKPVNLSELFYALLSNVVSRIALGRKYSIISEG------GKESSLKELFQ 212
CYP71AJ8        ---K---ESGSKAVNLSELFYALLSNVVSRIALGRKYAIITEG------GRESSFKELFQ 209
CYP71AJ7        ---R---ESGSKAVNLSELFYALLSNVVSRIALGRKYAITTEG------GKGNSFKELFQ 210
CYP71AJ15       ---R---ESGSRTVNLSELFYTLLSNVVSRIALGRKYAITTEG------GKENAFKEVFQ 210
CYP71AJ6        ---R---ESGSKTVNLSELFYTLLSNVVSRIALGRKYAITTEG------GKENSFKELFQ 210
CYP71AJ5        ---R---ESGSKTVNLSELFYTLLSNVVSRIALGRKYAITTEG------GKENSFKELFQ 211
CYP71AJ4        ---E---NSASEVINLGEQLIQLTRNVVCRVSVGSEYLSGHKG--------K-LYQKLLA 197
CYP71AJ21       ---E---HPPSKIVNLSDLLAELAQNVVCRVALGRKYGRGIDG--------NSSYKILLG 210
CYP71AJ25       ---E---YPPSKIVNLSDLLAELTQNVVCRVALGRKYESGDKG--------N-SYKILLG 209
CYP71AJ3        ---E---NSKSKVINLSEMLIELTGNVVCRAALGSGYN------------VDSYKSLLL 193
CYP71AJ1        ---E---NSHSKVANLTELLIEVTGNVVCRVSVGSGDK------------VDSYKILIL 205
CYP71AJ2        ---E---NSCSKVINLTELLIEVTGNVVCKVSVGSGDK------------VDSYKILIL 194
                              .           :: .

R1              RLMQAFQT-LFIPGYWF-----------LPTKNNRRMRAIDREICTILRGIIEKKDRAIK 291
R4              RAMKCMQK-ISIPGYMS-----------LPIENNRKMHQINKEIESILRGIIGKKMQAMK 296
R11             RLMSIVHK-FGIPGYMS-----------LPTKNNRRMRQIKREVETILRGLIGKRMQAMK 293
R8              FFFATLVV-LNVCDFMPV--LKWFGYKGL----EKRMVLAHQKRNEFLNNLLDEFRQKKI 273
R2              KLIPHLGA-ANMWDYLPV--MRWFDVFGV----RNKILHAVSRRDAFLRRLIDAERRRLA 277
R9              EIIPHIGA-ANLWDYLPA--LRWFDVFGV----RRKILAAVSRRDAFLRRLIDAERRRLD 492
R12             EIIPHIGA-ANLWDYLPA--LRWFDVFGV----RRKILAAVSRRDAFLRRLIDAERRRLD 273
R3              SVMDAAGT-PDAANYFPF--LRFLDLQGNVKTFKVCTERLVRVFRGFIDAKIAEKSSQN- 272
R5              NIMVDSAK-TNLVDVFPV--LKKIDPQGIKRGMARHFSKVLGIFDQLIEERMRTGR---- 257
R10             NSAALMAG-FQPEDVLPDAPARFVRWA---TGMDKKIDDMADVWDKFLSEIVAAHKEKGA 261
R6              KIMRLLSA-FSVGDFFPS--LGWVDYL---TGLIPEMKTTFLAVDAFLDEVIAEHESS-- 272
R7              ETQELLGE-FNVADYFPG--MAWINKI---NGLDERLEKNFRELDKFYDKIIEDHLNSSS 259
CYP71AJ13       NIAQLIGY-FSFSDYIPW--LYWIDSL---SGLKKRVEKAANEIDAFLEGVIRDHSIA-- 263
CYP71AJ14       NIAQLIGY-FSFSDYIPW--LYWIDSL---NGLKGRVEKAANEIDAFLESVIRDHSTA-- 263
CYP71AJ12       NIAQLIGY-FSFSDYIPW--LYWIDSL---NGLKGRVEKAANEIDAFLEGVIRDHSTA-- 263
CYP71AJ11       NIAQLIGY-FSVGDYIPW--LFWVDSL---NGLKGRVEKASTEVDVFLEGVIRDHSVA-- 261
CYP71AJ9        SIAQLIGY-FSVGDYIPW--LFWIDSL---NGLKGQVEKASAEVDVFLEGVIRDHRIA-- 264
CYP71AJ8        SIAQLIGY-FSVGDYIPW--LFWVDSL---SGLKGRVEKASAEVDVFLEGVIRDHLIA-- 261
CYP71AJ7        NIAQLIGY-FSVGDYIPW--LFWIDSV---SGLKGRVEKAANEADLFLESVIKDHSIA-- 262
CYP71AJ15       NIAQLIGY-FSVGDYIPW--LFWVDSV---TGLKGRVEKAANEVDLFLEGVIKDHSIA-- 262
CYP71AJ6        NIAQLIGY-VSVGDYIPW--LFWIDSV---NGLKGRVEKAANEADLFLEGVIKDHSVA-- 262
CYP71AJ5        NIAQLIGY-FSVGDYIPW--LFWIDSV---NGLKGRVEKAANEADLFLEGVIKDHSIA-- 263
CYP71AJ4        EVTEMLAYTYSIGDFIPL--LGWVDWL---SGSKAKVEKTAKEVDAFLEGALRDHIKTMA 252
CYP71AJ21       EIMELIGYSRSMRDFFPL--LGWVDRL---TGLNARAEKAAKEVDTFLEGVLRDHPSTVA 265
CYP71AJ25       EIMELLGYSRSMGDFFPL--LGWVDWL---NGLKAKVEKAANEVDTFLEGVLRDHPSTVA 264
CYP71AJ3        QIMDMLGYSRSIEDFFPS--LGWVDWI---TGLKGKVEKAANGVDAFLEGVLKNHTNPS- 247
CYP71AJ1        EIMDMLGYSRSIEDFFPL--LGWVDWL---TGLRGKVAEAAKGVDTFLEGVLKEHLSTT- 259
CYP71AJ2        EIMEMLGYSRSIEDFFPM--FGWVDWL---TGLRGKVAKAAKGVDDFLEGVLKEHLTARA 249
                             .                                : 
```

```
R1              S---------GEASSDDLLGLLLESNRRESN---GKADLGMSTEDIIEECKLFYFAGMET  339
R4              E---------GESTKDDLLGILLESNTKHMEENGQS-SQGLTMKDIVEECKLFYFAGAET  346
R11             E---------GEPTKDDLLGLLLESNMKETTENGQSSSLGMTIEDVMEECKLFYFAGMET  344
R8              AGISESSTDSINAKKTTLVETLLSLQESEP--------EFYTDDLIKSVLLVLFIAGTET  325
R2              DGGSD-------GDKKSMIAVLLTLQKTEP--------KVYTDTMITALCANLFGAGTET  322
R9              D-GDE-------GEKKSMIAVLLTLQKTEP--------EVYTDNMITALTANLFGAGTET  536
R12             D-GDE-------GEKKSMIAVLLTLQKTEP--------EVYTDNMITALTANLFGAGTET  317
R3              --------NPKDVSKNDFVDNLLD-YKGDE--------SELSISDIEHLLLDMFTAGTDT  315
R5              ------------FEQGDVLDVCLKMMQDNP--------NEFNHTNIKALFLDLFVAGTDT  297
R10             D-------DGAGEDDEDFLDVLLRLRREG------SHGLELTDDRIKATVEDLIAAATET  308
R6              -----------NKKNDDFLGILLQLQECG------RLDFQLDRDNLKAILVDMIIGGSDT  315
R7              W--------MKQRDDEDVIDVLLRIQKD-P-----NQEIPLKDDHIKGLLADIFIAGTDT  305
CYP71AJ13       L--------STGASSDDLLNTLLEIQKQDT-----NSAFSIDNDSIKGVILNMYFDGTDS  310
CYP71AJ14       L--------TNGASSDDLLKTLLEIEKQDS-----NSAFSIDKDSIKGVILNMYFDGTDS  310
CYP71AJ12       L--------INGASSDDLLKTLLEIQKQDS-----NSAFSIDKDSIKGVILNMYFDGTDS  310
CYP71AJ11       L--------DNGASSDDLLYNLLEIQKQNT-----NSAFSIDKDSIKGVILNMFFDGTDT  308
CYP71AJ9        L--------DNGASSDDLLYNLPEIQKQNT-----NSAFSIDKDSIKGVILNMFFDGTDT  311
CYP71AJ8        L--------ENGASRDDLLYNLLEIQKQNN-----DSAFSIDKDSIKGVILNMFFDGTDT  308
CYP71AJ7        L--------DKGASSDDLLYNLLEIQKQDT-----NSAFSIDKDSIKGVILNMFFDGTDT  309
CYP71AJ15       L--------DNGVSSDALLYNLLEIQNQDT-----NSAFSIDKDSIKGVILNMFFDGTDT  309
CYP71AJ6        L--------DNGASSDDLLYNLLEIQKQDT-----NSTFSIDKDSIKGVILNMFFDGTDT  309
CYP71AJ5        L--------DNGVSTDDLLYNLLEIQKQDT-----NSAFSIDKDSIKGVILNMFFDGTDT  310
CYP71AJ4        S--------NKGSANDDFLSILLEIREADA-----G--STLDEECIKAIVWDMILGGTET  297
CYP71AJ21       S--------NNGHANKDFVSILLEIQNTDA-----G--SSMDKDCIKAVIWDMFVAGTDT  310
CYP71AJ25       S--------NNGYANKDFVSILLEIQNTDA-----G--SSMDKDCIKALIWDMFGAGTDT  309
CYP71AJ3        ----------TSSANKDFVSILLEIQEADA-----G--SSMDKECIKSLIWDMLGAGTET  290
CYP71AJ1        -----------GSKYNDFVSILLEIQEADA-----G--SSMDNECIKSLIWDMLGAGTET  301
CYP71AJ2        S--------NNASADNDFVSILLEIQEADA-----G--STMDNECIKSLIWDMLGAGTET  294
                                 .:                          :      :   . ::

R1              TSVLLTWTLIVLSMHPEWQEQARKEVLHHFGR---TKPDFENLSRLKIVTMVLYEVLRLY  396
R4              TSVLLTWAMLLLSMHPEWQDRAREEILGLFRK---NKPDYEGLSRLKIVTMILYEVLRLY  403
R11             TSVLLTWTMILLSMHPEWQDRAREEVLGLFGK---NKPGYDGLSRLKIVTMILYEVLRLY  401
R8              TSMTIQWAMRLLLAHPKAFTKLRAEIDSKVGN--DGLLNESDIPKLPYLHRVINETLRLY  383
R2              TSTTTEWAMSLLLNHPAALKKAQAEIDASVGT--SRLVSVDDVPSLAYLQCIVSETLRLY  380
R9              TSTTSEWAMSLLLNHPDTLKKAQAEIDASVGN--SRLITADDVTRLGYLQCIVRETLRLY  594
R12             TSTTSEWAMSLLLNHPDTLKKAQAEIDASVGN--SRLITADDVTRLGYLQCIVRETLRLY  375
R3              SSSTLEWAMTELLKNPKTMAKAQAEIDCVIGQ--NGIVEESDISKLPYLQAVVKETFRLH  373
R5              TSITIEWAMTELLRKPHIMSKAKEELEKVIGK--GSIVKEDDVLRLPYLSCIVKEVLRLH  355
R10             SSQTLEWAMAELVANPRVMAKLRDEIARVATA-DQQTIAESDLNRMEYLKAVFKEVLRLH  367
R6              TSTTLEWTFAEFLRNPNTMKKAQEEVRRVVGINSKAVLDENCVNQMNYLKCVVKETLRLH  375
R7              SSTTIEWAMSELIKNPRVLRKAQEEVREVAKG--KQKVQESDLCKLEYLKLVIKETLRLH  363
CYP71AJ13       TSAVLEWTMAALIKHPDIMCKLKDEVREIGRG--KSRISGDDLEKMHYLRAVIKESMRIY  368
CYP71AJ14       TSAVLEWTMAALIKHPDIMCKLKNEVREIGRG--KSRINGDDLEQMHYLRAVIKESMRLY  368
CYP71AJ12       TSAVLEWTMAALIKHPDIMCKLKNEVREIGRG--KPRINGDDLEQMHYLRAVIKESMRLY  368
CYP71AJ11       TSAVLEWTMAALIKHPDIMRKLQNEVRDIGRG--KSTISGDDLENMHYLKAVIKESMRIY  366
CYP71AJ9        TSAVLEWTMAALIKNPDIMRKLQNEVREIGRG--KSTISGDDLENMHYLKAVIKESMRIY  369
CYP71AJ8        TSAVLEWTMAALIKNPDIMHKLQSEVREIGRG--KSTISGDDLENMHYLKAVIKESMRIC  366
CYP71AJ7        TSAVLEWTMAALIKHPDVMCKLKNEVREIGRG--KLRINGDDLENMHYLKAVVKESMRLY  367
CYP71AJ15       TSAVLEWTMAALIKHPDIMCKLKNEVREIGRG--KSKISGDDLEKMHYLKAVVKESMRIY  367
CYP71AJ6        TSAVLEWTMAALIKHPDIMCKLKNEVREIGRG--KSKICGDDLEKMHYLKAVVKESMRIY  367
CYP71AJ5        TSAVLEWTMAALIKHPDIMCKLKNEVREIGRG--KSKISGDDLEKMHYLKAVVKESMRVY  368
CYP71AJ4        TSTTLEWIVAAIIKNPDVMFKLQKEVREIGKG--KSKIEEVDLVKMNYLKAVMKESMRLY  355
CYP71AJ21       TSSTLEWAIAALIKNPHVMVKLQTEIREIGRG--KSKITEGDLAKMHYLKAVMKESMRLY  368
CYP71AJ25       TSTTLEWTIAALIKSPDVMVKLQKEVREIGRG--KSNISEDDLVKMNYLKAVIKESMRLY  367
CYP71AJ3        IATALEWTIGALIKSPDAMSKLQKEVREIGKG--KSRIEEGDLVKMDYLKAVMKESMRLY  348
CYP71AJ1        ISTALEWTLAALIKNPDAMFKLQNEVREIGKG--KSKISEADLVKMNYLQAVMKESMRLY  359
CYP71AJ2        ISTALEWTLAALIKNPDAMLKLQNEVREIGKG--KSKISEADLGKMTYLQAVMKESMRLY  352
                 :    * .  :   *    : : *:                :  :  :  :. * :*:
```

```
R1              PPA-IFVTRRTYKAMELGGITYPAGVNLMLPILFIHHDPNIWGKDASEFNPQRFADGIS-  454
R4              PPF-IEIGRKTYKEMEIGGVTYPAGVSIKIPVLFIHHDPDTWGSDVHEFKPERFSEGIS-  461
R11             PPA-IAFSRKTYKEMEIGDATYPAGVILELPVLHIHHDPDIWGSDVHEFRPERFAEGIA-  459
R8              PPVPLLLPHYSLEDCTVGGYEVPKHTILMVNAWAIHRDPKLWD-EPEKFKPERFEA---M  439
R2              PAAPLLLPHESSADCKVGGYNVPADTMLIVNAYAIHRDPAAWE-HPLEFRPDRFED---G  436
R9              PAAPMLLPHESSADCKVGGYNIPRGSMLLINAYAIHRDPAVWE-EPEKFMPERFED---G  650
R12             PAAPMLLPHESSADCKVGGYNIPRGSMLLINAYAIHRDPAVWE-EPEKFMPERFED---G  431
R3              TPVPLLIPRKAESDAEILGFMVLKDTQVLVNVWAIGRDPSVWD-NPSQFEPERFLG---K  429
R5              PPSPLLLPRKVVTQVELSGYTIPAGTLVFVNAWAIGRDPTVWD-DSLEFKPQRFLE---S  411
R10             APAPLLVPHESTTPAVVQGYEIPAKTALFINVWAIGRDPAAWD-APEEFRPERFVVGGKP  426
R6              PPLPLLIARETSSSVKLRGYDIPAKTMVFINAWAIQRDPELWD-DPEEFIPERFET---S  431
R7              PPAPLLVPRVTTASCKIMEYEIPADTRVLINSTAIGTDPKYWE-NPLTFLPERFLD---K  419
CYP71AJ13       TPVPLLVAREAMQDVKVMGYDIKAGTQVLINAWAIATDGAVWD-NPEEFIPERFLN---N  424
CYP71AJ14       TPVPLLVAREAMQDVKVMGYDIKAGTQVLINAWAIATDPAVWD-NPGEFIPERFLN---N  424
CYP71AJ12       TPVPLLVAREAMQDVKVMGYDIKAGTQVLINAWAIATDPAVWD-NPEEFIPERFLN---N  424
CYP71AJ11       TPVPLLVAREAMQDVNVMGYDIKAGTQVLINAWAIATDPTLWD-NPEEFIPERFLN---N  422
CYP71AJ9        TPVPLLVAREAMQDVNVMGYYIKAGTQVMINAWAIATDPTLWD-NPEEFIPERFLN---N  425
CYP71AJ8        TPVPLLVAREAMQDVNVMGYDIRAGTQVMINAWAIATDPTVWD-KPEEFIPERFLN---N  422
CYP71AJ7        TPVPLLVAREAMQDVKVMGYDVTDGTQVLINAWAIATDPALWD-NPEEFIPERFLN---N  423
CYP71AJ15       TPVPLLVAREAMQDVKVMGYDVKAGTQVLINAWAIATDPALWD-NPQEFIPERFLN---N  423
CYP71AJ6        TPVPLLVAREAMQDVKLMGYDVKSGTQVLINAWAIATDPALWD-NPEEFIPERFLN---N  423
CYP71AJ5        TPVPLLVAREAMQDVKVMGYDVKAGTQVLINAWAIATDPALWD-NPEKFIPERFLN---N  424
CYP71AJ4        ITA-FLLPREAKQDVKLMGYDISSGTQVLINTWETARDPSLWD-NPEEFRPERFLN---S  410
CYP71AJ21       FTAPLLMPREARQDVKVMGYDIRSGTQVLVNAWAIARDPALWN-NPEEFRPERFFN---N  424
CYP71AJ25       FTAPLIVPREARQDVKVMGYDIRKGTQVLINAWAIARDPSLWE-NPEKFQPERFLN---S  423
CYP71AJ3        FTAPLLVPREARQDVKFMGYDIKSGTQVLINAWAIARDPSSWD-NPEEFRPERFLN---S  404
CYP71AJ1        FTAPLLVPREARQDIKFMGYDISSGTQVLINAWAIARDPLLWD-KPEEFRPERFLN---S  415
CYP71AJ2        FTAPLLVPRESRQDVKFMGYDISAGTQVLINVWAIARDPSLWE-KPEEFRPERFLN---S  408
                    : . :       .          : :       *   *      * *:**

R1              --NAVKHPAAFFPFGGGPRICIGQNFALLEAKMALSTILQRFSFELSPSYTHAPYTV---  509
R4              --KASKDPGAFLPFGWGPRICIGQNFALLEAKMALCLILQRLEFELAPSYTHAPHTM---  516
R11             --KASRDRLAFFPFGWGPRICIGQNFALLEAKIALSMMLQSFEFELAPSYTHAPRTV---  514
R8              EGEKEGFNYKLVPFGMGRRACPGAAMGLRTVSLVLGSLIQSFDWKSVEEEK-----LDAC  494
R2              KAE----GLFMIPFGMGRRRCPGETLALRTIGMVLATLVQCFDWEPVDGVK-----VDMT  487
R9              GCD----GNLLMPFGMGRRRCPGETLALRTVGLVLGTLIQCFDWERVDGVE-----VDMT  701
R12             GCD----GNLLMPFGMGRRRCPGETLALRTVGLVLGTLIQCFDWERVDGVE-----VDMT  482
R3              DMDVRGRDYELTPFGAGRRICPGMPLAMKTVSLMLASLLYSFDWKLPKGVL--SEDLDMD  487
R5              RLDVRGHDFDLIPFGAGRRICPGIPLATRMVPIMLGSLLNNFDWKIDTKVP--YDVLDMT  469
R10             PVDFRGTDYQLIPFGAGRRICPGINFALPVLELALVSLLRHFDWELPDGMR--PVDLDMG  484
R6              QVDLNGQDFQLIPFGIGRRGCPAMSFGLASTEYVLANLLYWFNWNMSESGRILMHNIDMS  491
R7              EIDYRGKNFELLPFGAGRRGCPGINFSIPLVELALANLLFHYNWSLPEGML--PKDVDME  477
CYP71AJ13       PIDYKGLHFEFIPFGAGRRGCPGIQYAMAINELALANLVHIFDLALPDGKR--FENLDMD  482
CYP71AJ14       SVDYKGLHFEFIPFGAGRRGCPGIQYAMAINELALATLVHIFDFALPDGKR--FEDLDMA  482
CYP71AJ12       SVDYKGVHFEFIPFGAGRRGCPGIQYAMAINELALANLVHIFDFALPDGKR--FEDLDMA  482
CYP71AJ11       PVDYKGLHFEFIPFGAGRRGCPGIQYAMAINELALANLVHIFDFALPDGKR--FEDLDMT  480
CYP71AJ9        PVDHKGMHFEFIPFGARRRGCPGIQYAMAINELALANLVHIFDFALPGGKR--LEDLDMD  483
CYP71AJ8        PLDYKGLHFEFIPFGAGRRGCPGIQYAMAINELALANLVHIFDFALPDGKR--FEDLDMD  480
CYP71AJ7        PIDYKGLHFEFIPFGAGRRGCPGIQYAMAINELAVANLVHIFDFALPDGRR--LEDLDLT  481
CYP71AJ15       PTDYKGLHFEFIPFGAGRRGCPGIQYAMAINELALANLVHIFDFALPDGRR--LEDLDMT  481
CYP71AJ6        PIDYKGLHFEFIPFGAGRRGCPGIQYAMAINELALANLVHIFDFALPDGRR--LEDLDMT  481
CYP71AJ5        PIDYKGLHFEFIPFGAGRRGCPGIQYAMAINELALANLVHIFDFALPDGRR--LEDLDMT  482
CYP71AJ4        PIDYKGLHYEYLPFGGGRRGCPGIQFAMAVNELAVANVVYKFDFKMPDGER--FEDLDMS  468
CYP71AJ21       PIDYKGLHYEYLPFGAGRRVCPGIQFAVAVNELAVANLVHKFDFELPHGER--MEDMDMT  482
CYP71AJ25       PIDYKGLHYEYLPFGGGRRGCPGIQFAMAVNELAVANVVHKFDFELPNGET--REDLDMT  481
CYP71AJ3        PIDYKGFNYEYIPFGAGRRGCPGIQFAISVNELVVANVVNKFNFELPDGKR--LEEMDMT  462
CYP71AJ1        PIDYKGFHYEFLPFGAGRRGCPGIQFAMCINELVVANLVHKFNFELPDGKR--LEDLDMT  473
CYP71AJ2        HIDYKGFNYEYLPFGAGRRGCPGIQFAMAVNELVVANVIHKFNFELPDGER--LEDLDMT  466
                  .         ***   * * .   .       :  ::   .             :
```

```
R1              ----LTL---HPQHGAPIVLRKI--------  525
R4              ----VTL---HPMHGAQIKVRAI--------  532
R11             ----IML---RPMHGAQIKLRAI--------  530
R8              YNSRITLNKDKPLEAVCIPRQNWRGFLS---  522
R2              EGGGFTIPKAVPLEAVCRPRAVMRDVLQNL-  517
R9              EGGGLTIPKVVPLEAMCRPRDAMGGVLRELV  732
R12             EGGGLTIPKVVPLEAMCRPRDAMGGVLRELV  513
R3              ETFGLTLHKTNPLHAVPVKKRANIN------  512
R5              EKNGTTISKAKPLCVVPIPLN----------  490
R10             EAPGLTTPRRVPLVLVPRCKTLAQPALQ---  512
R6              ETNGLTVSKKVPLHLEPEPYKT---------  513
R7              EALGITMHKKSPLCLVASHYNLL--------  500
CYP71AJ13       SETGMTVHKKSPLLVIATPCI----------  503
CYP71AJ14       SETGMTVHKKSPLLVIATPRI----------  503
CYP71AJ12       SETGMTVHKKSPLLVIATPRI----------  503
CYP71AJ11       AETGMTLHKKSPLLAIATSRV----------  501
CYP71AJ9        AETGMTLHKKSPLLVVATSRV----------  504
CYP71AJ8        AETGMTLHKKSPLLVIATSRV----------  501
CYP71AJ7        SETGMTLHKKSPLMVIATSRV----------  502
CYP71AJ15       SETGMTLHKKSPLLVIATSRV----------  502
CYP71AJ6        SETGMTLHKKSPLLVIATSRV----------  502
CYP71AJ5        SETGMTLHKKSPLLVIATSRV----------  503
CYP71AJ4        GVPGISLYRK---------------------  478
CYP71AJ21       GVTGLTVRRKSPLLVIATPHV----------  503
CYP71AJ25       GVTGITLRRKSPLLVIATPHV----------  502
CYP71AJ3        ASTGITFHKKS--------------------  473
CYP71AJ1        AASGITLRKKSPLLVVARPHV----------  494
CYP71AJ2        AVSGITLRKK---------------------  476
```
