# Supplementary material for: Genome-Wide Evolutionary Analysis of Putative Non-Specific Herbicide Resistance Genes and Compilation of Core Promoters between Monocots and Dicots
Source: Genes (Basel). 2022 Jun 29;13(7):1171. doi: 10.3390/genes13071171 (PMC9316059; doi:10.3390/genes13071171)
Supplement: Supplementary file 1 [file genes-13-01171-s001.zip › Supplementary file S7.pdf]

Supplementary file S7: Homology modelled 3D structure of Resistant GST genes

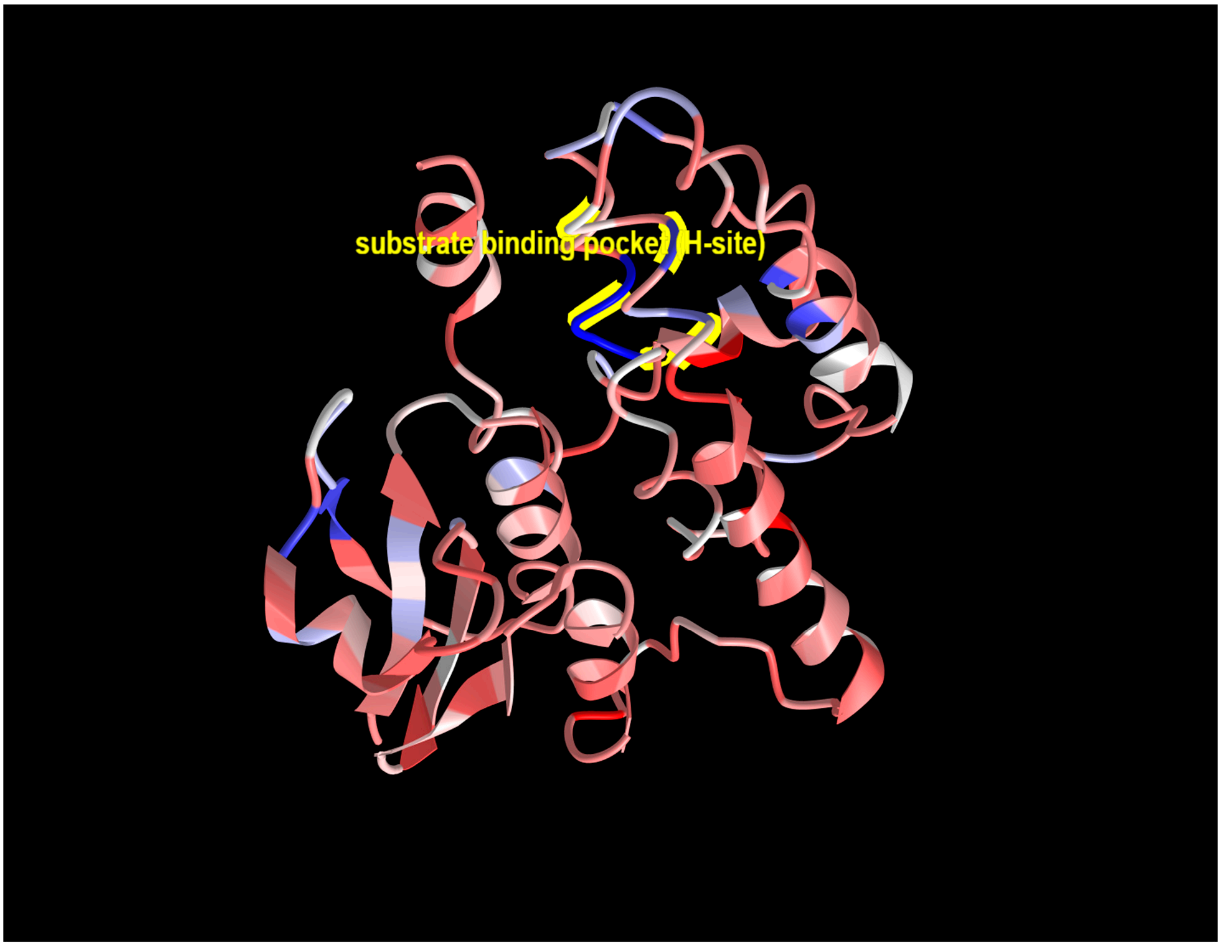

Figure 1: 3D homology modelling of resistant GST gene Resistant 1. The Substrate binding pocket (H-site) is highlighted in yellow

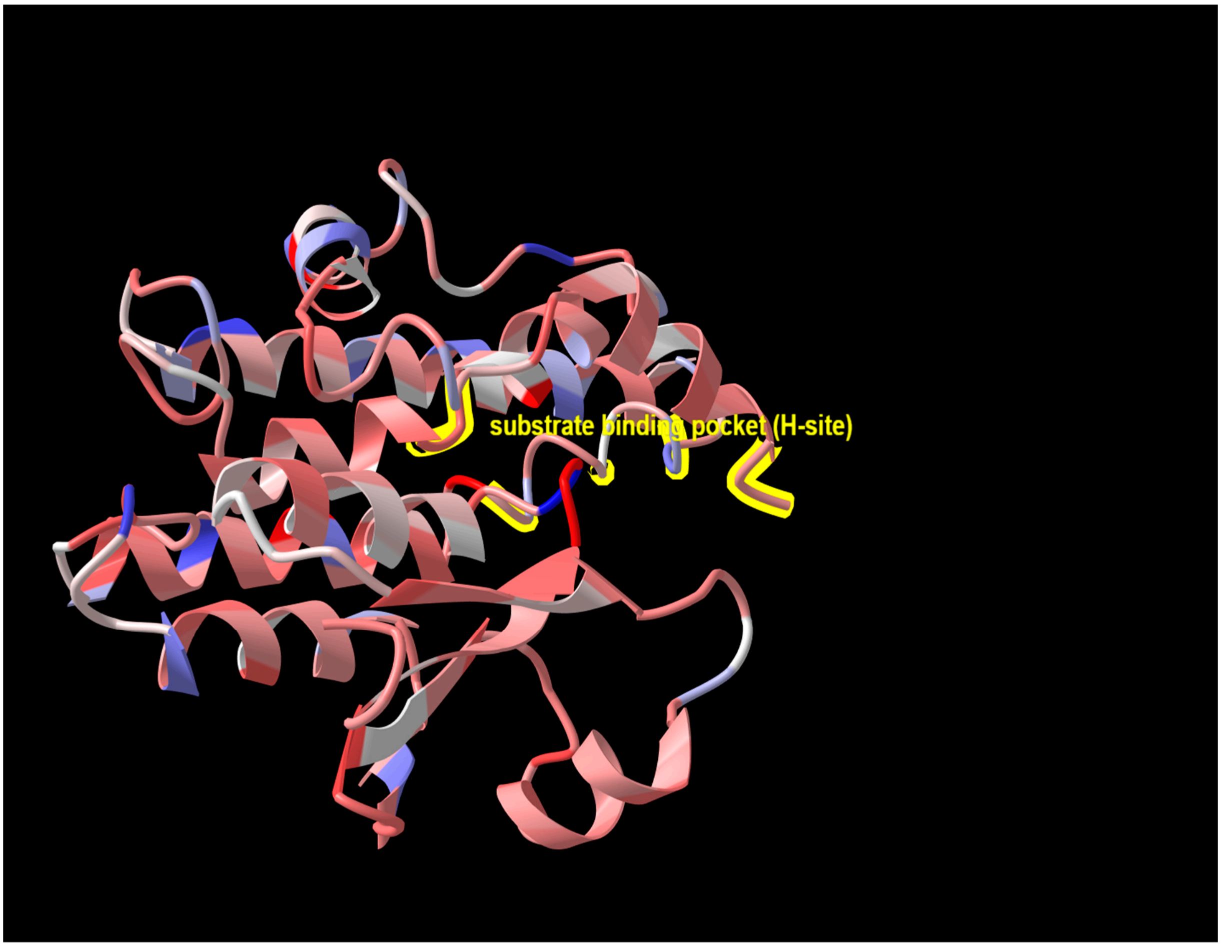

Figure 2: 3D homology modelling of resistant GST gene Resistant 2. The Substrate binding pocket (H-site) is highlighted in yellow

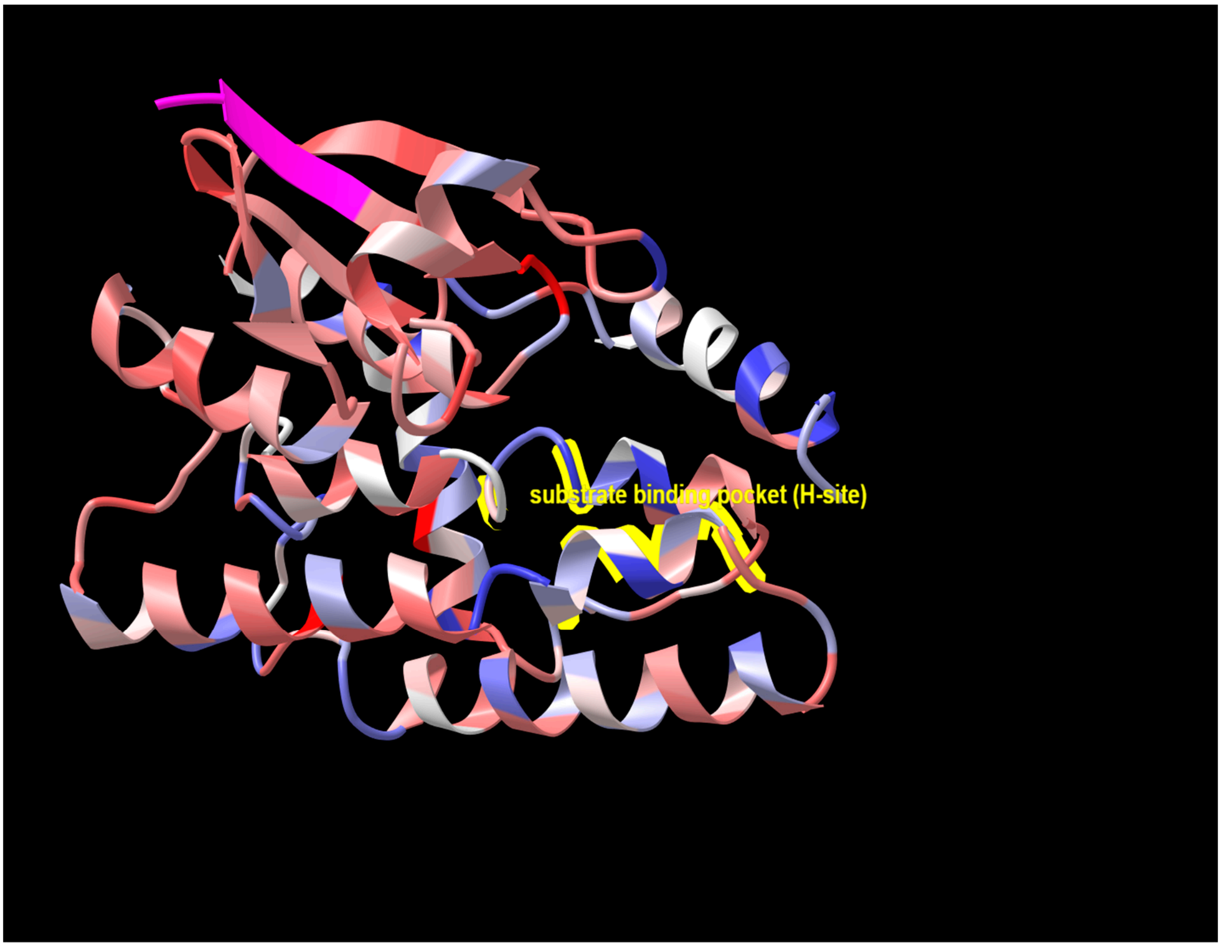

Figure 3: 3D homology modelling of resistant GST gene Resistant 3. The Substrate binding pocket (H-site) is highlighted in yellow

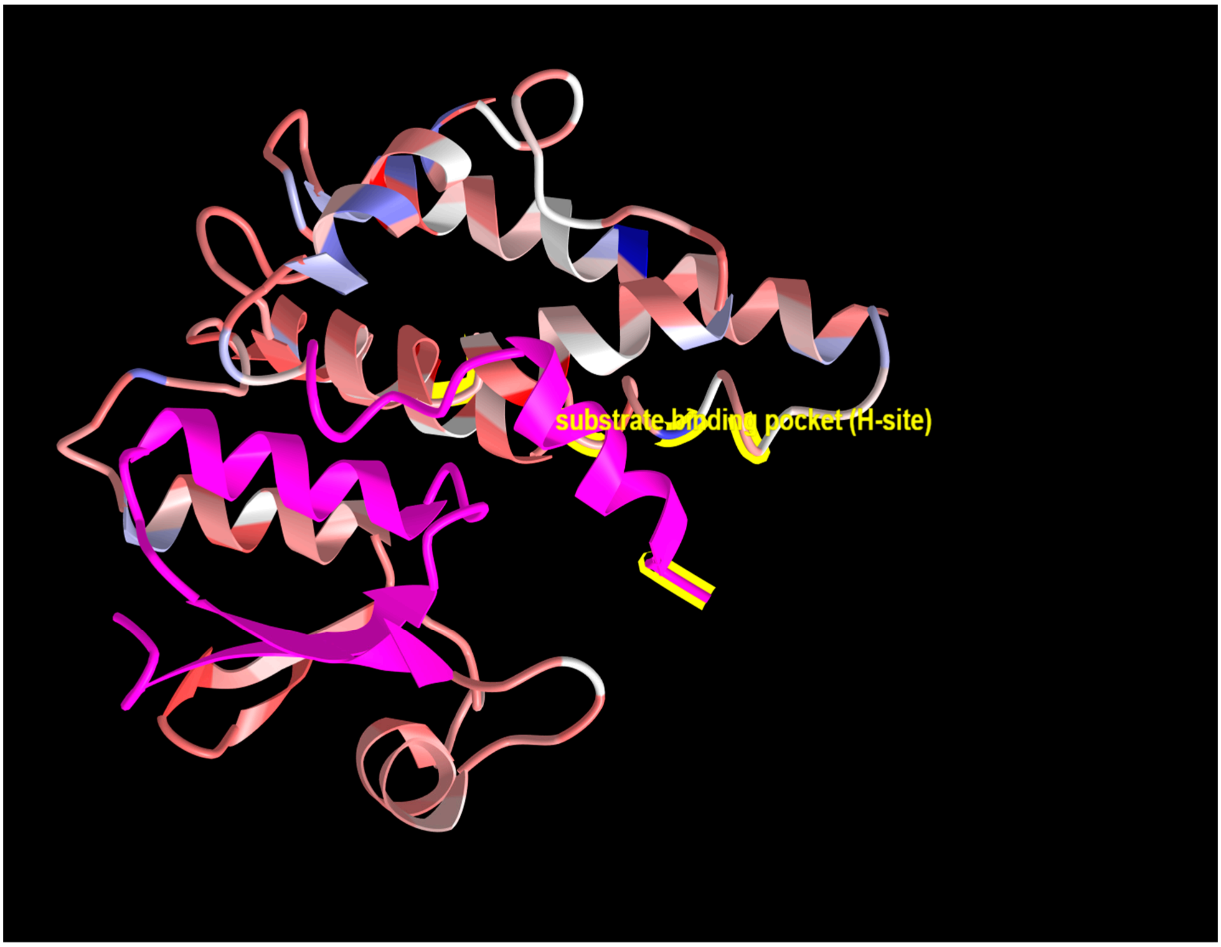

Figure 4: 3D homology modelling of resistant GST gene Resistant 4. The Substrate binding pocket (H-site) is highlighted in yellow

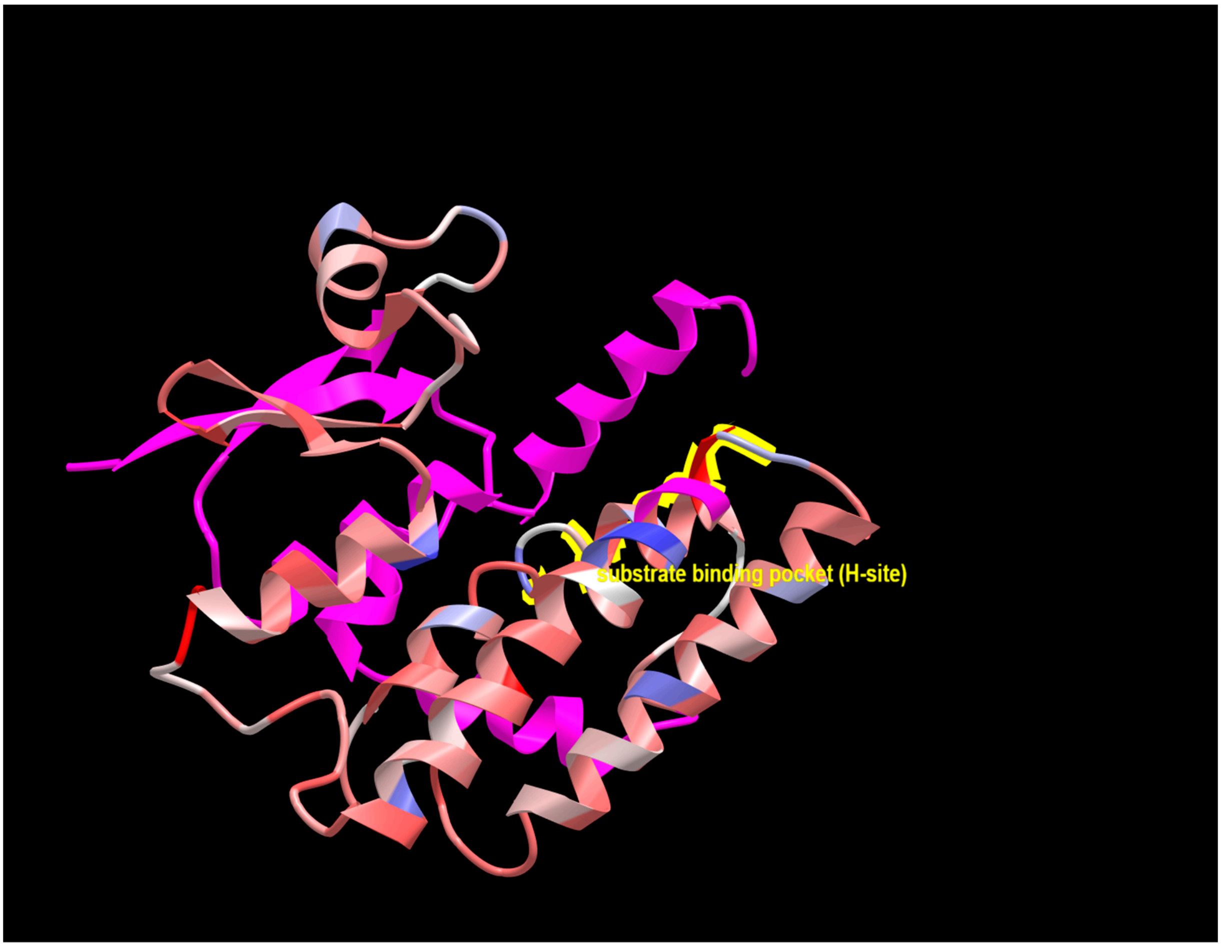

Figure 5: 3D homology modelling of resistant GST gene Resistant 5. The Substrate binding pocket (H-site) is highlighted in yellow

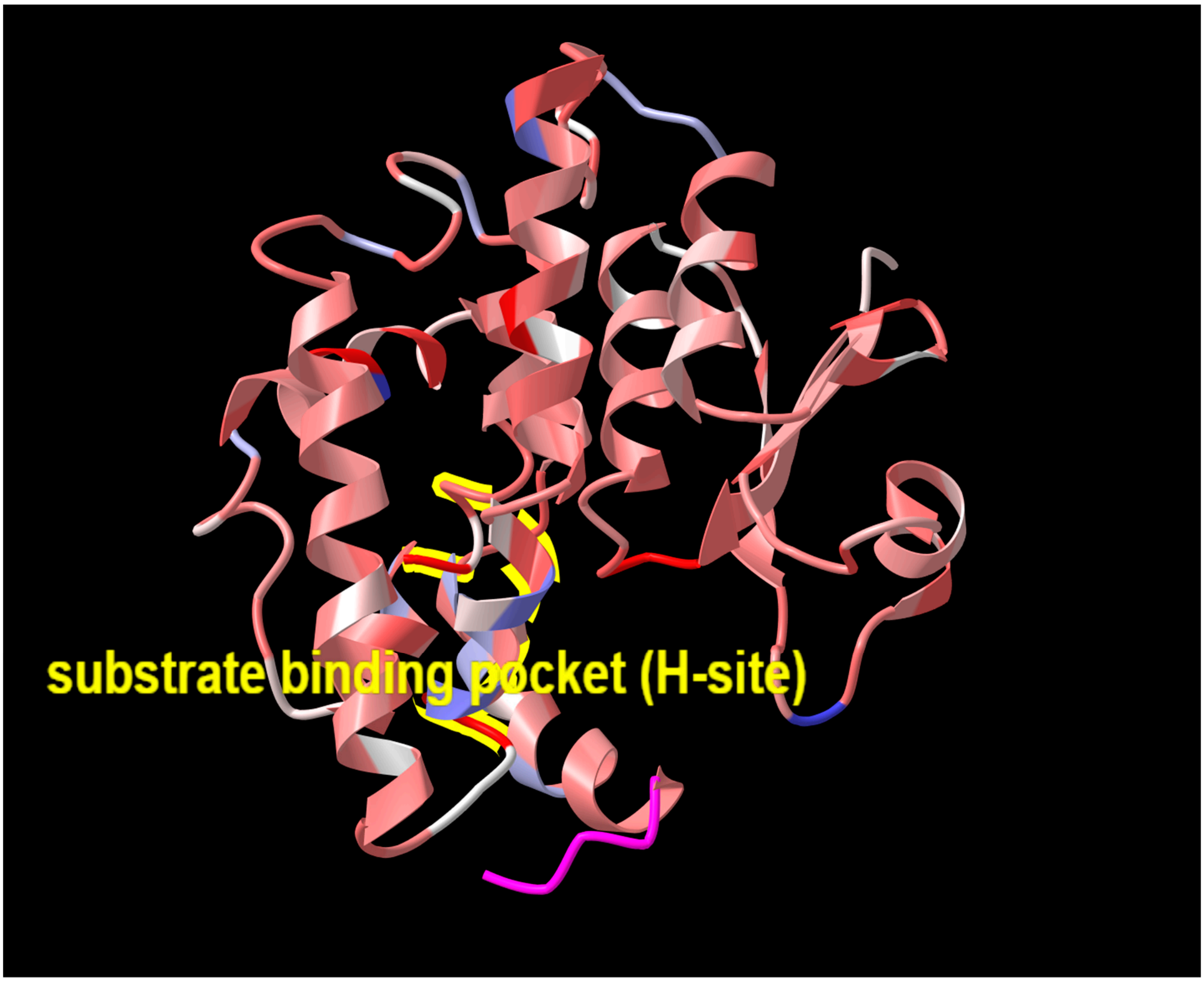

Figure 6: 3D homology modelling of resistant GST gene Resistant 6. The Substrate binding pocket (H-site) is highlighted in yellow

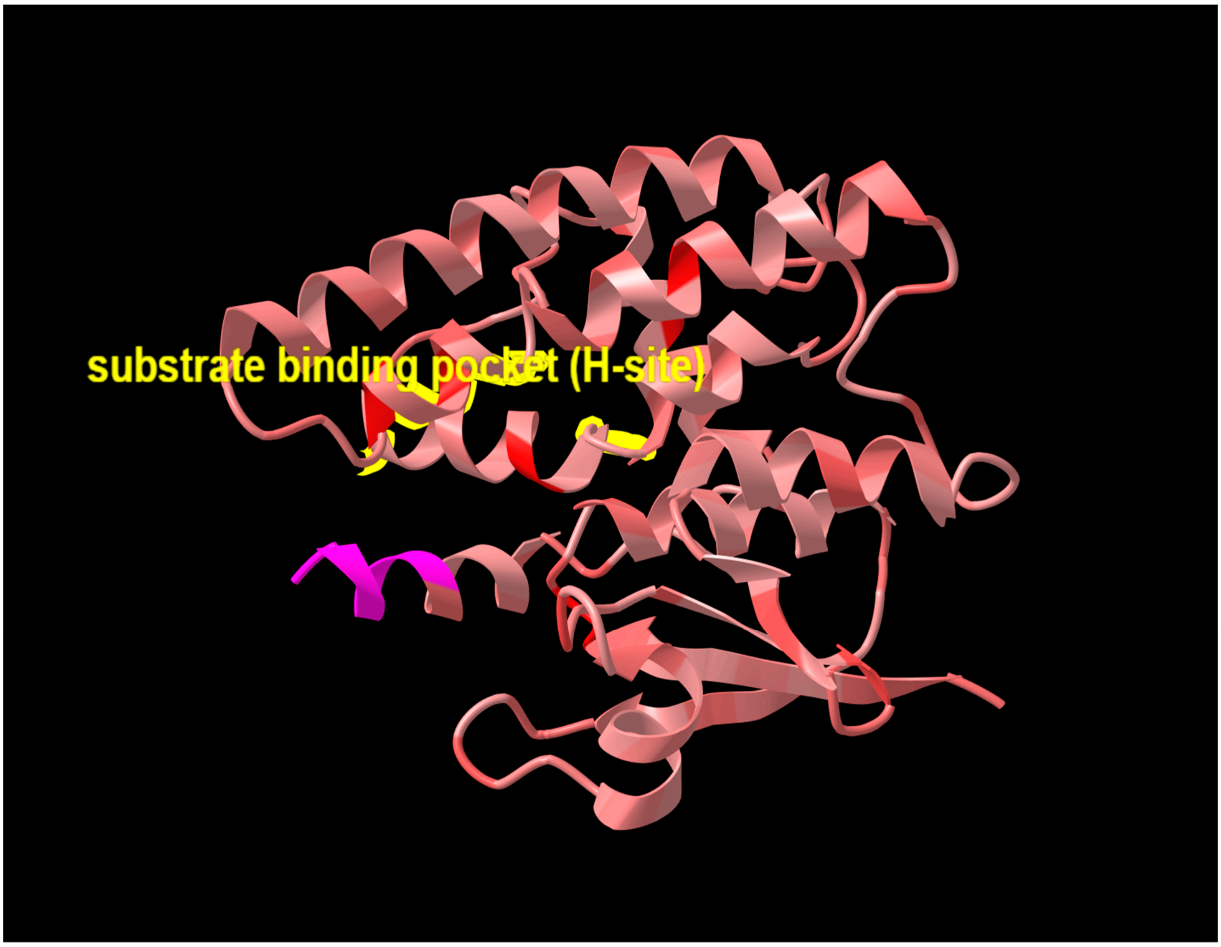

Figure 7: 3D homology modelling of resistant GST gene Resistant 7. The Substrate binding pocket (H-site) is highlighted in yellow

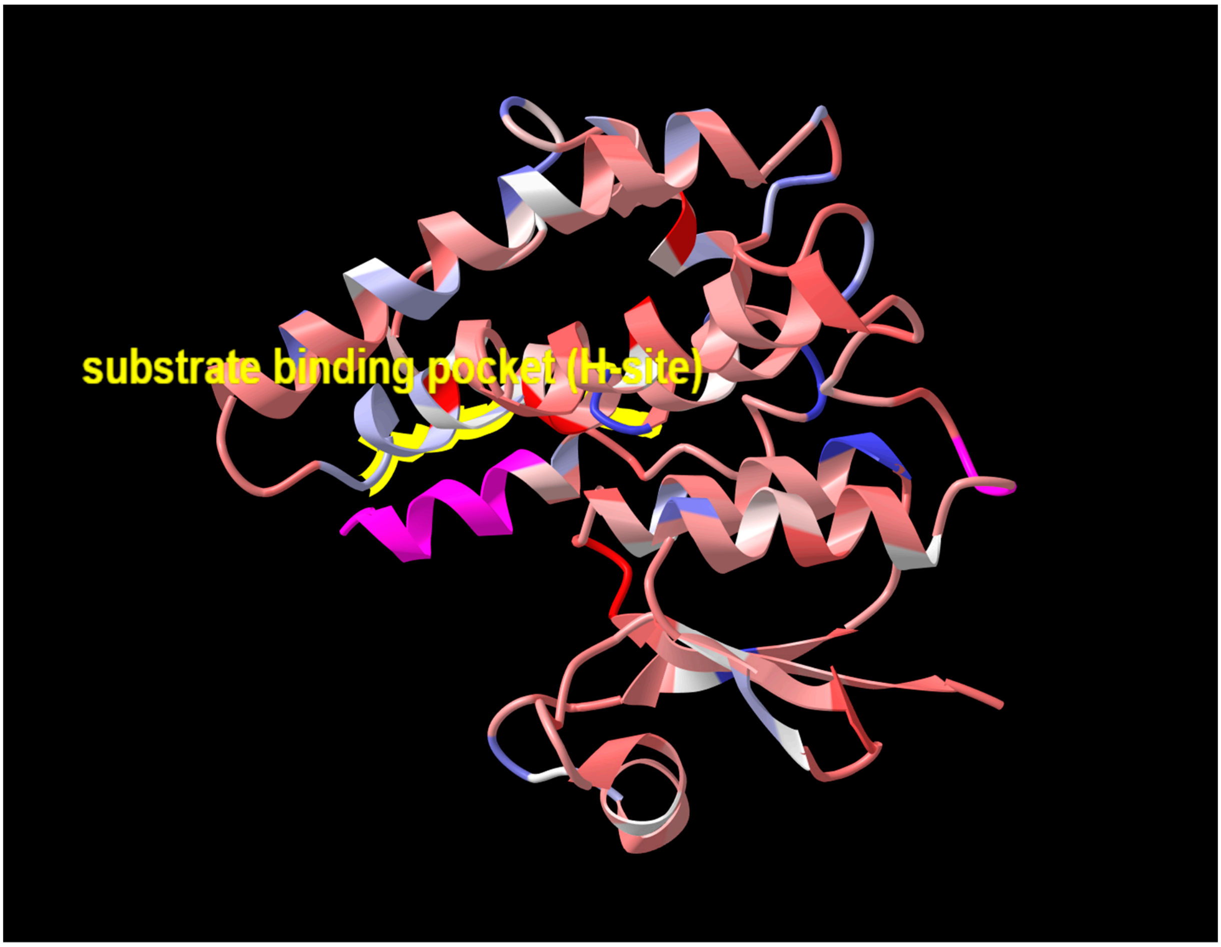

Figure 8: 3D homology modelling of resistant GST gene Resistant 8. The Substrate binding pocket (H-site) is highlighted in yellow

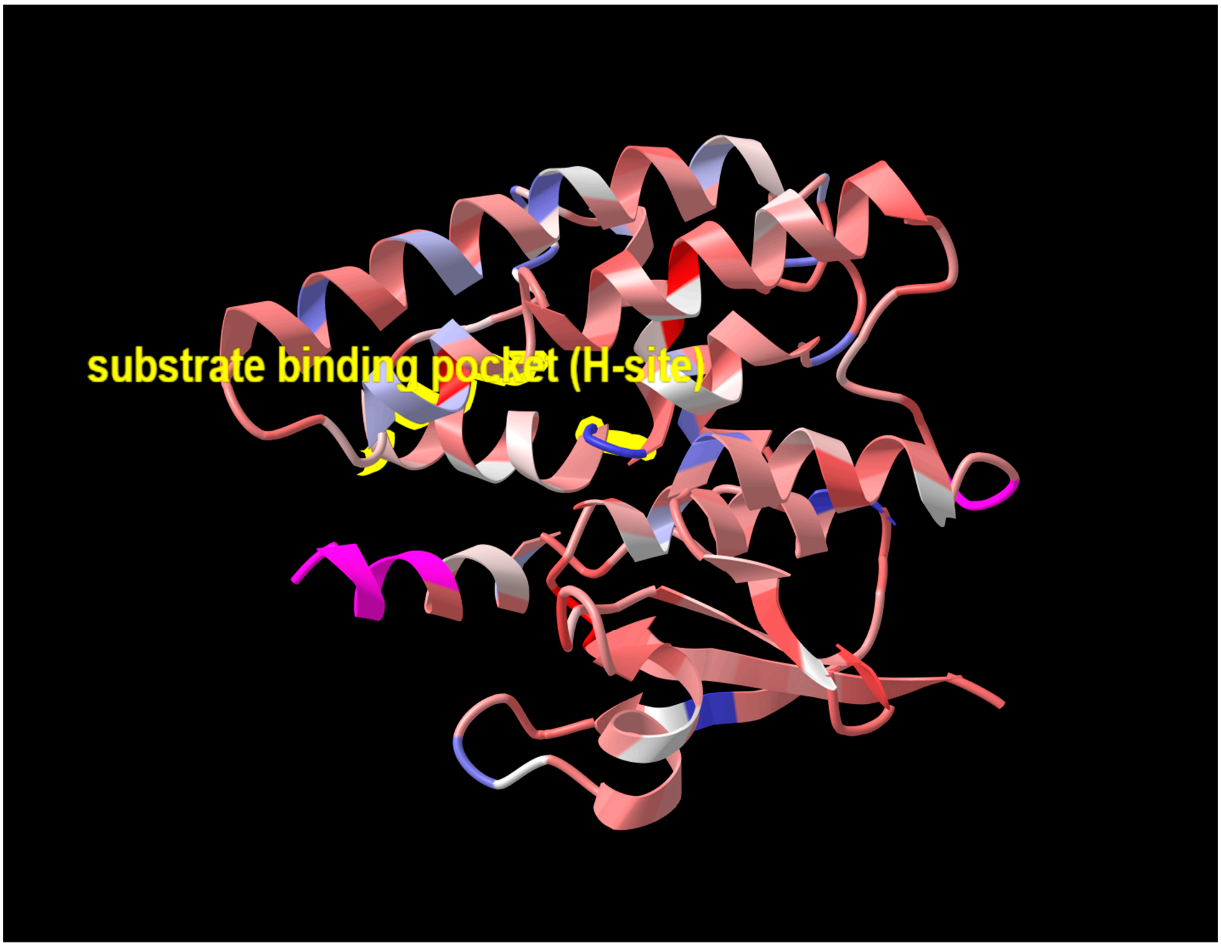

Figure 9: 3D homology modelling of resistant GST gene Resistant 9. The Substrate binding pocket (H-site) is highlighted in yellow

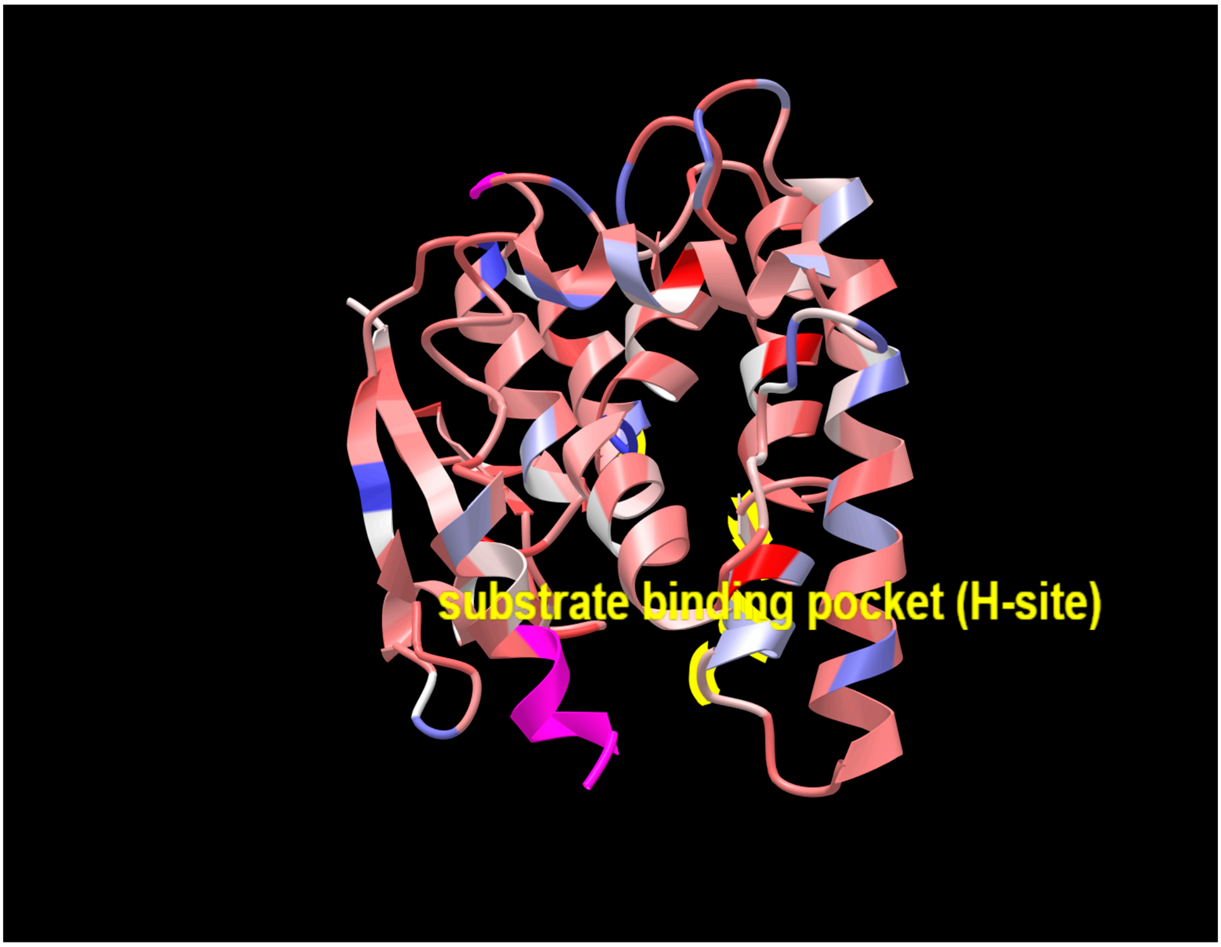

Figure 10: 3D homology modelling of resistant GST gene Resistant 10. The Substrate binding pocket (H-site) is highlighted in yellow

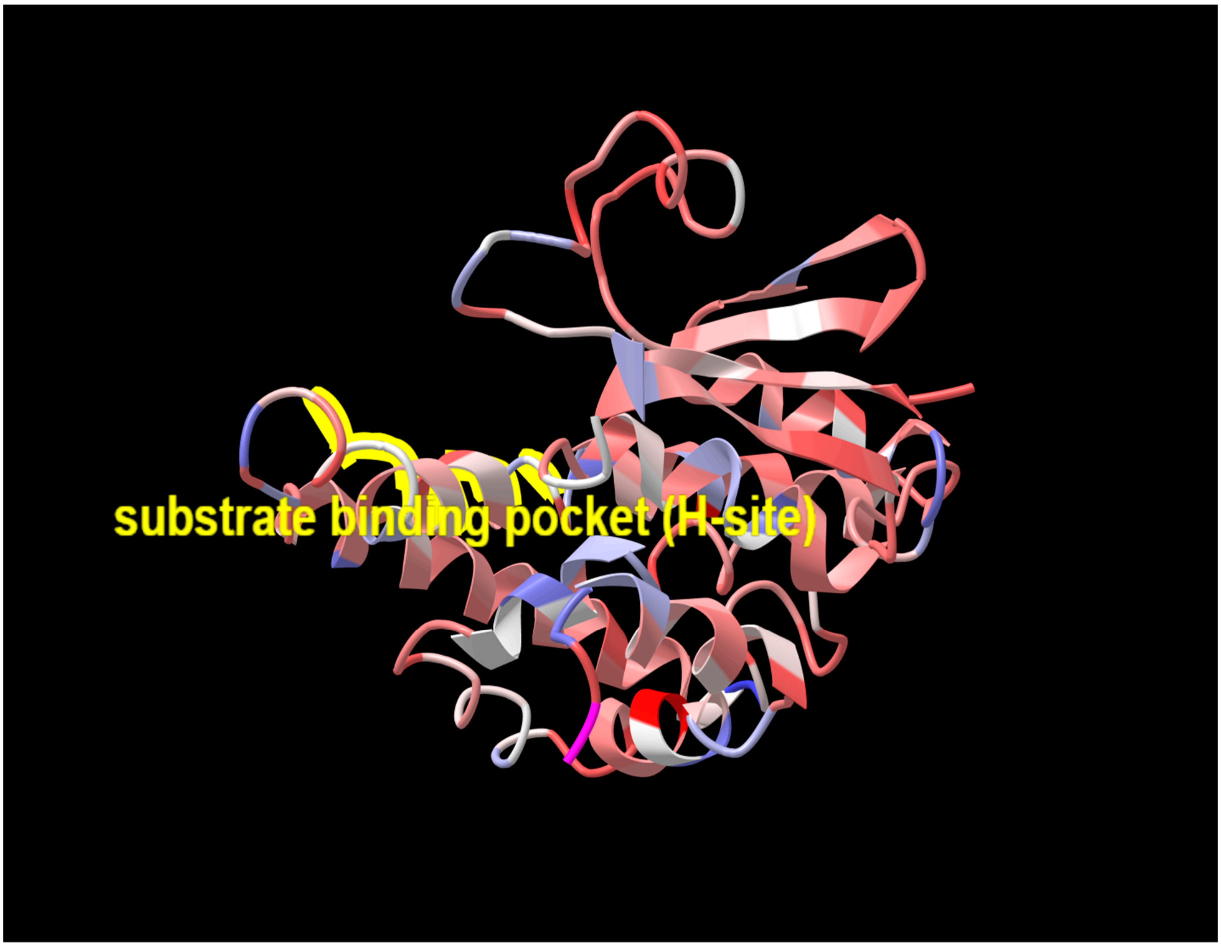

Figure 11: 3D homology modelling of resistant GST gene Resistant 11. The Substrate binding pocket (H-site) is highlighted in yellow

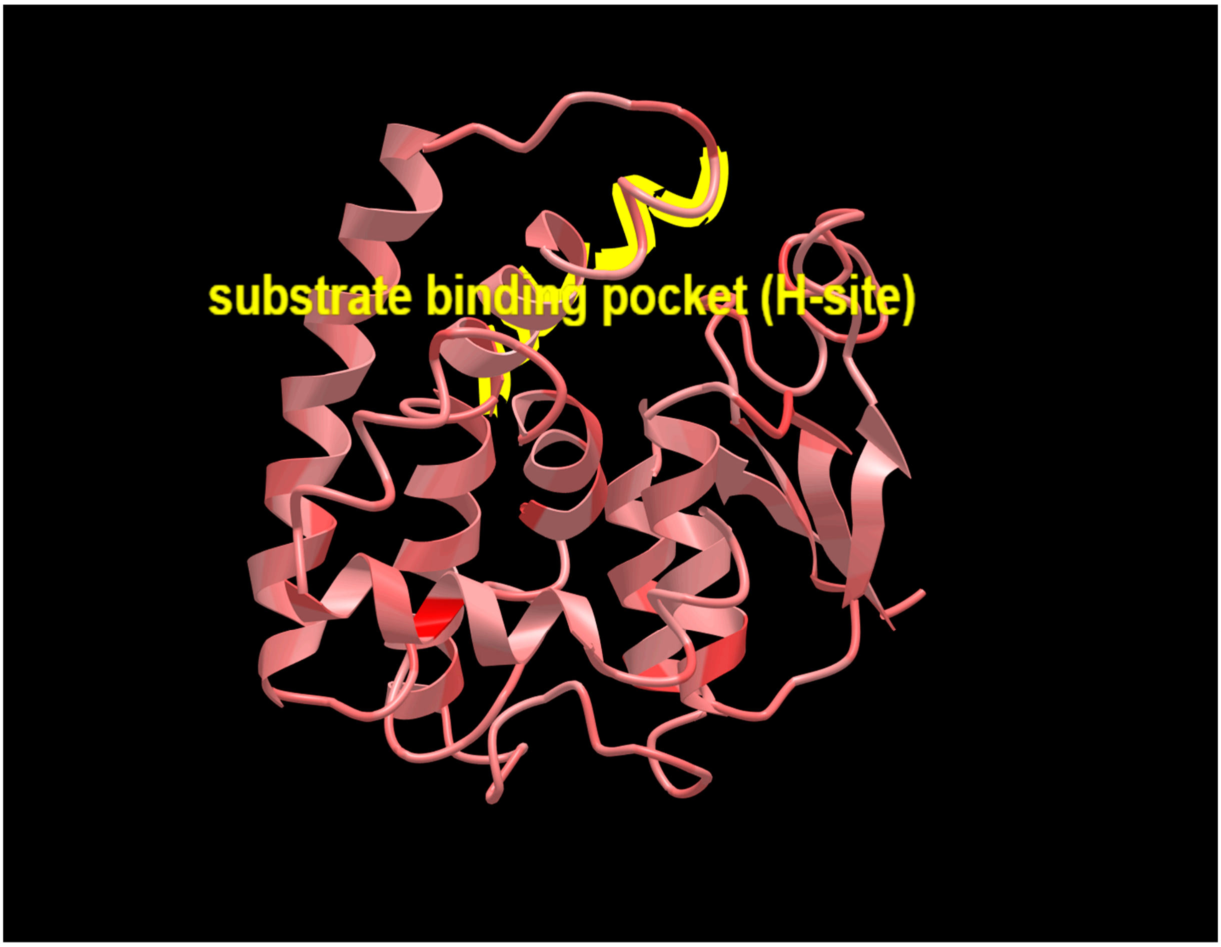

Figure 12: 3D homology modelling of resistant GST gene Resistant 12. The Substrate binding pocket (H-site) is highlighted in yellow

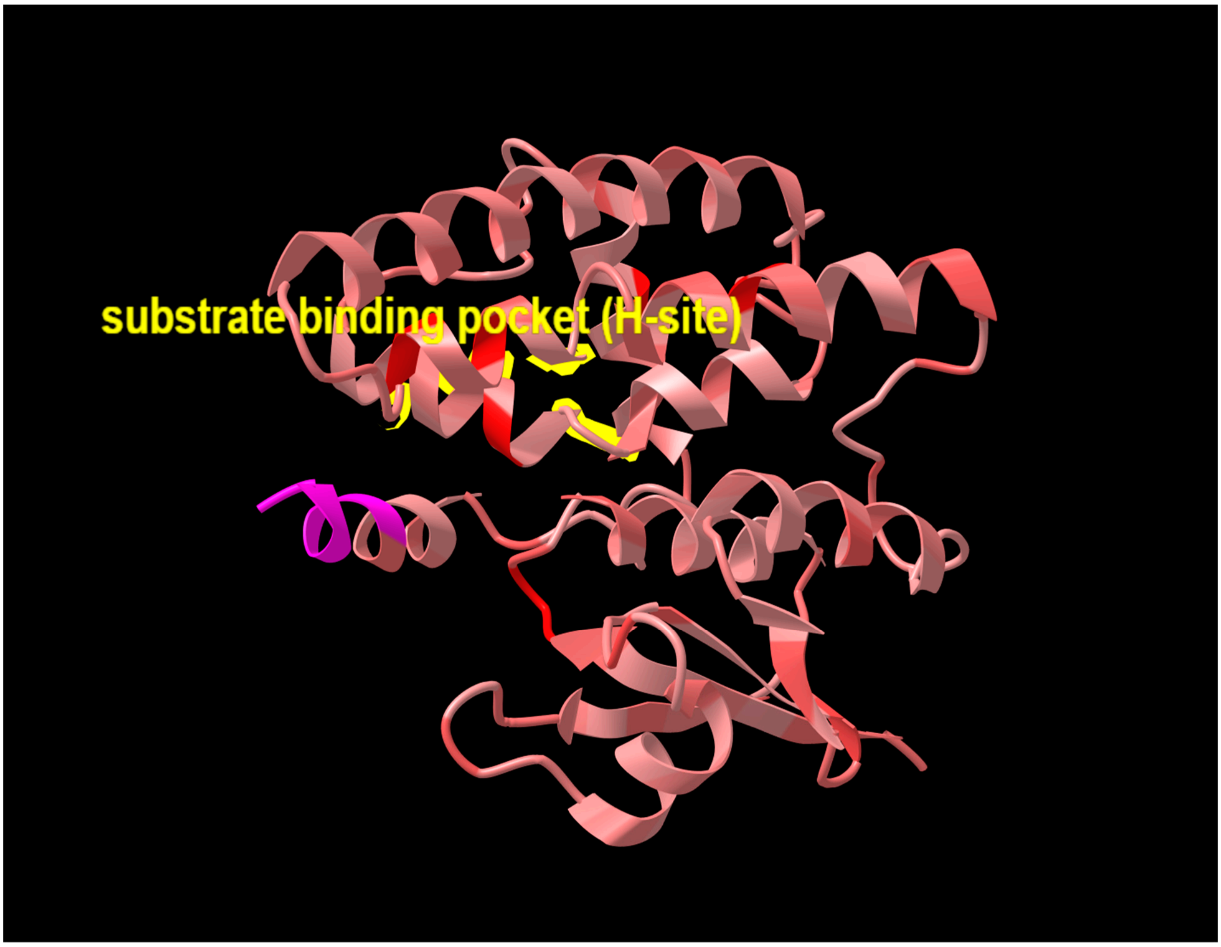

Figure 13: 3D homology modelling of resistant GST gene Resistant 13. The Substrate binding pocket (H-site) is highlighted in yellow

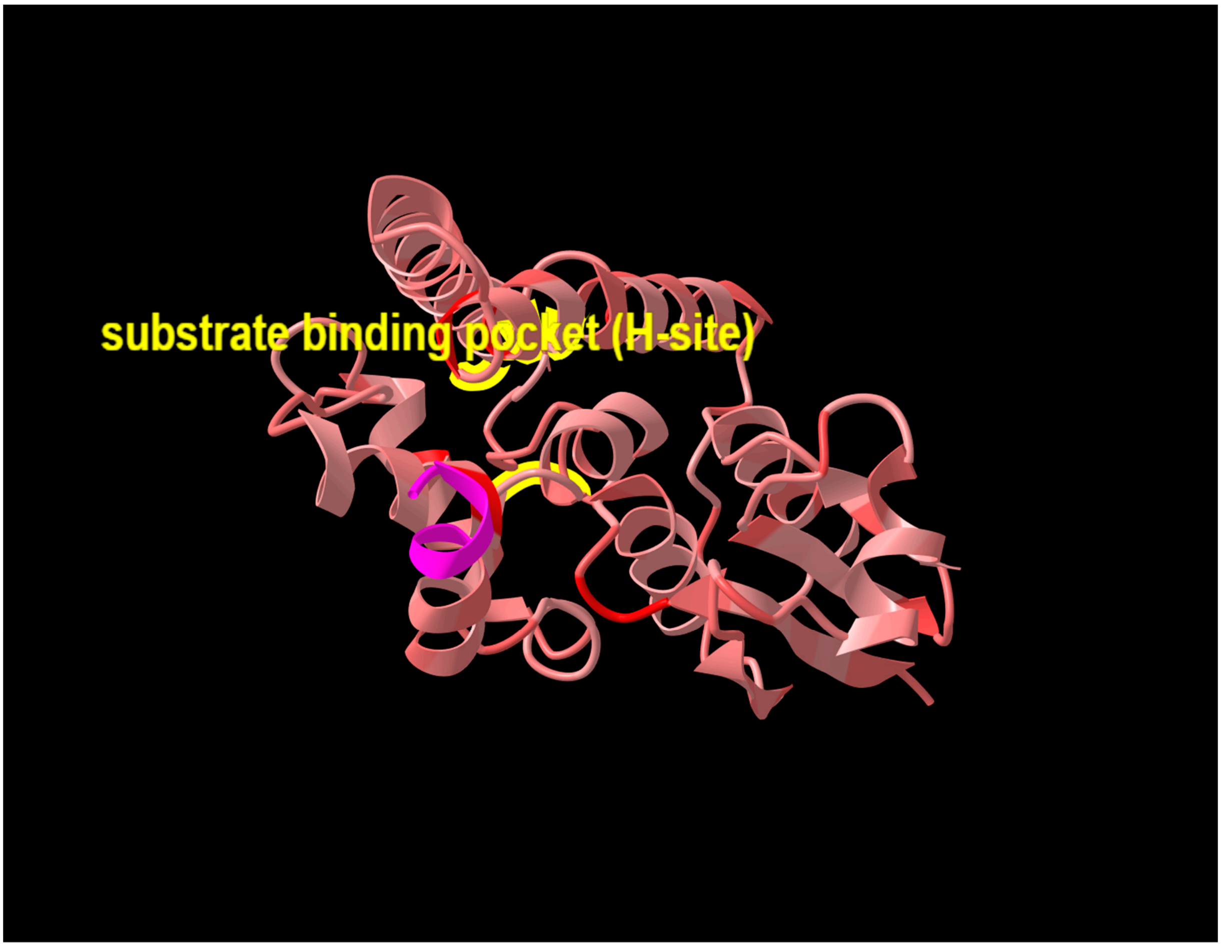

Figure 14: 3D homology modelling of resistant GST gene Resistant 14. The Substrate binding pocket (H-site) is highlighted in yellow

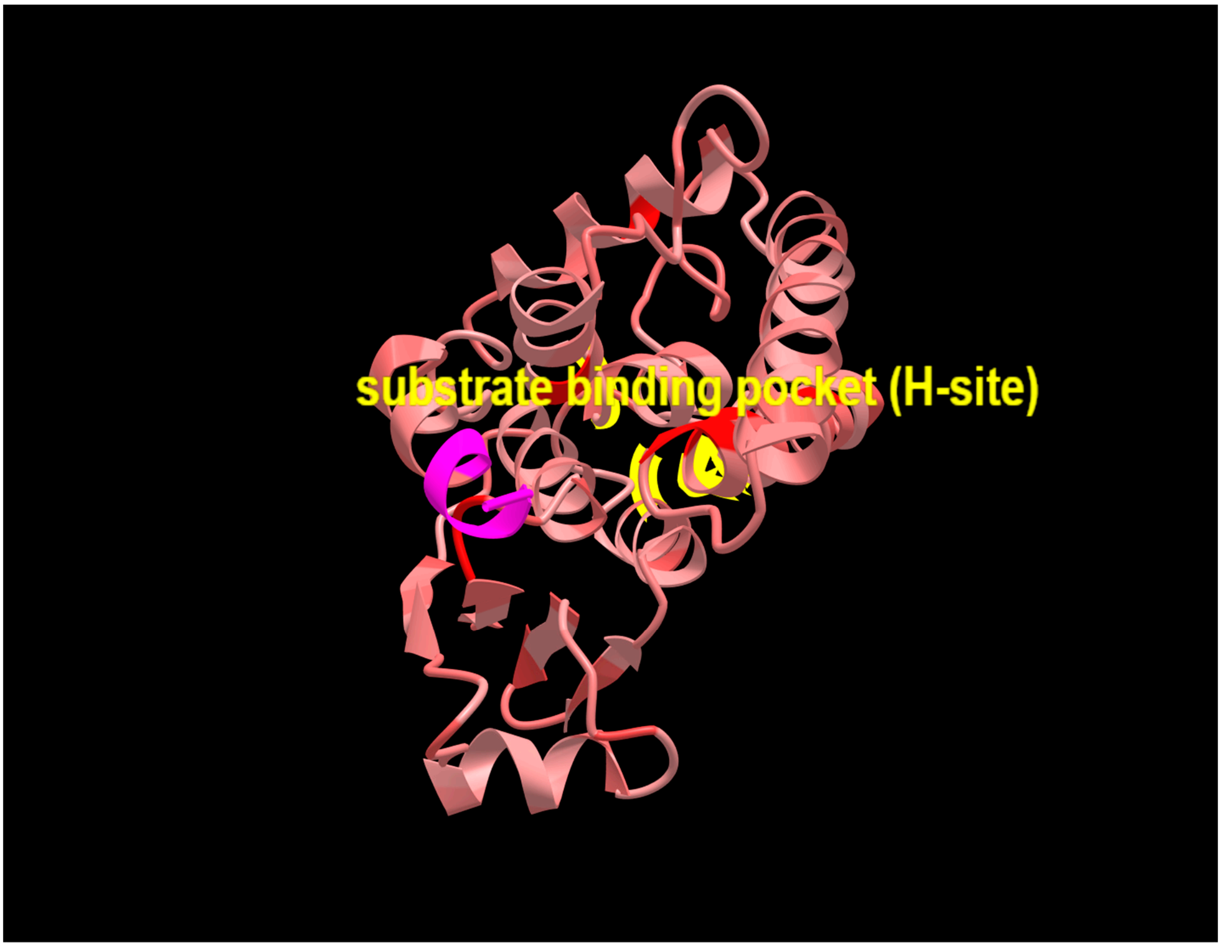

Figure 15: 3D homology modelling of resistant GST gene Resistant 15. The Substrate binding pocket (H-site) is highlighted in yellow

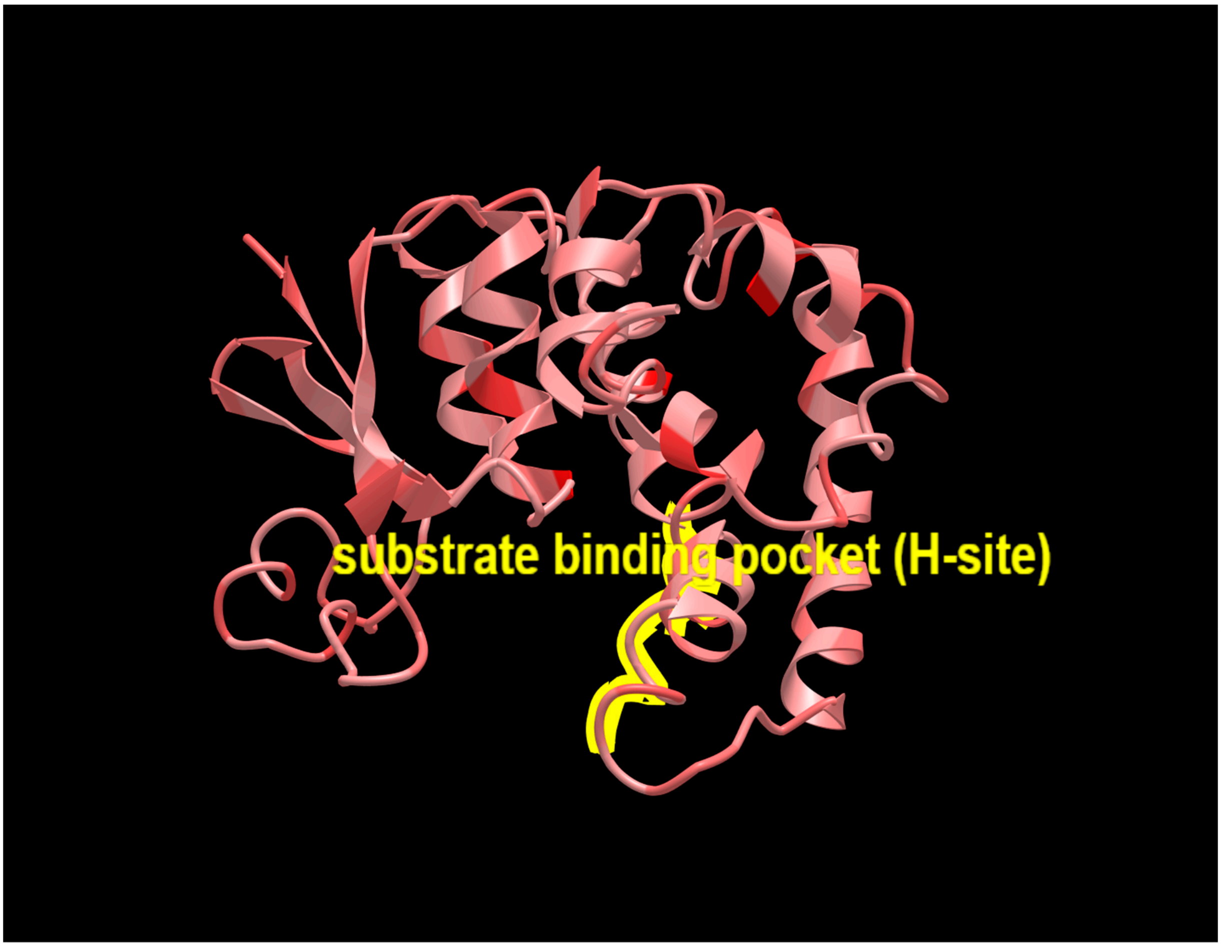

Figure 16: 3D homology modelling of resistant GST gene Resistant 16. The Substrate binding pocket (H-site) is highlighted in yellow

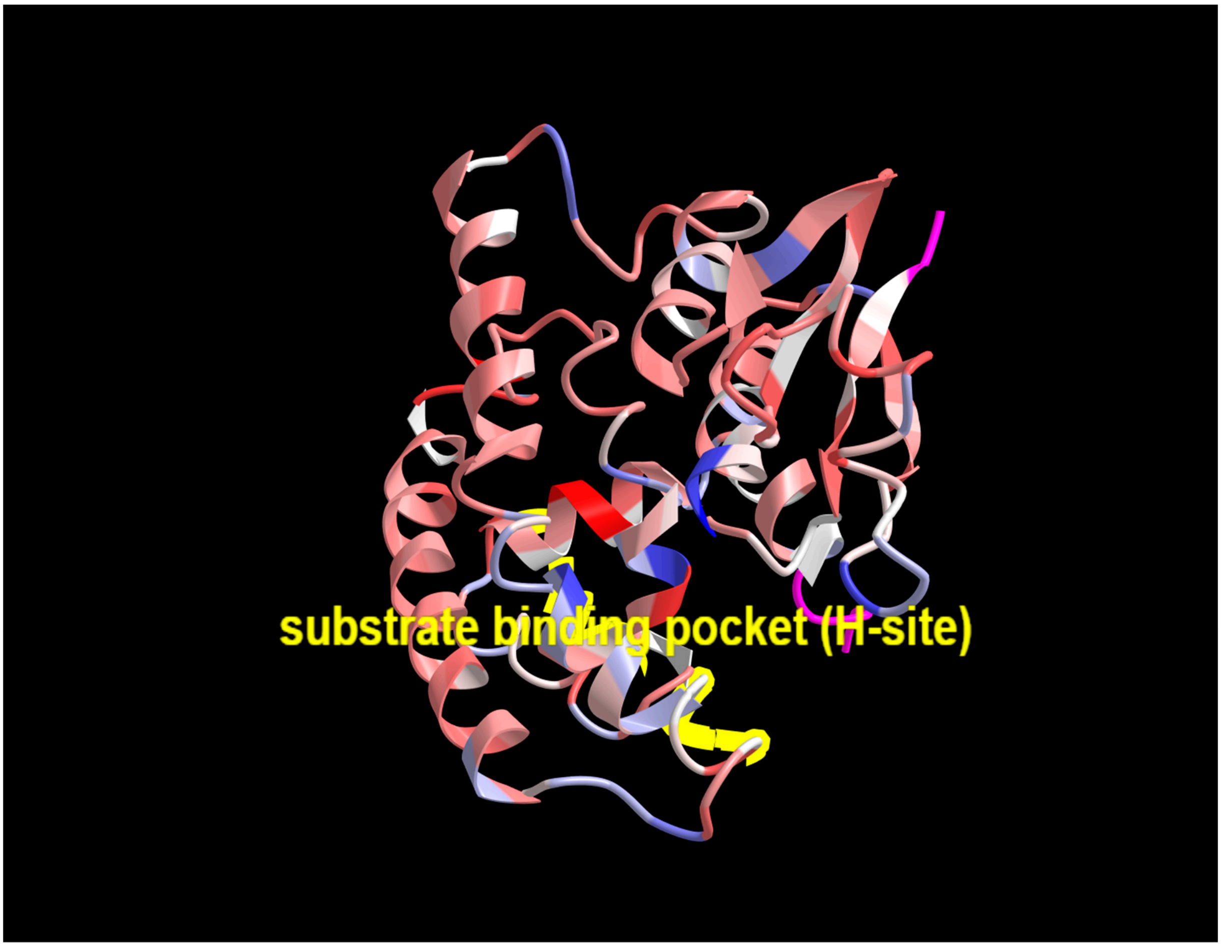

Figure 17: 3D homology modelling of resistant GST gene Resistant 17. The Substrate binding pocket (H-site) is highlighted in yellow

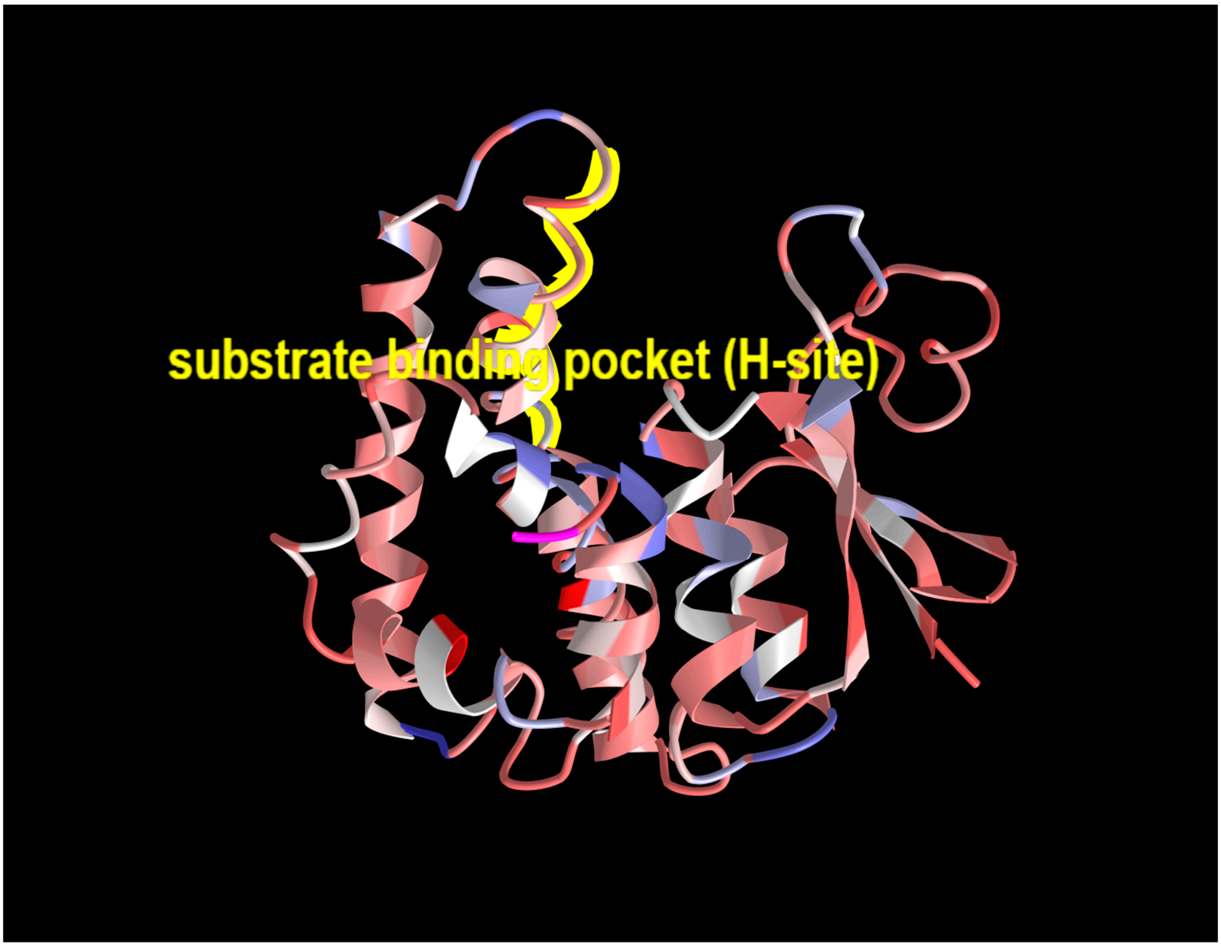

Figure 18: 3D homology modelling of resistant GST gene Resistant 18. The Substrate binding pocket (H-site) is highlighted in yellow

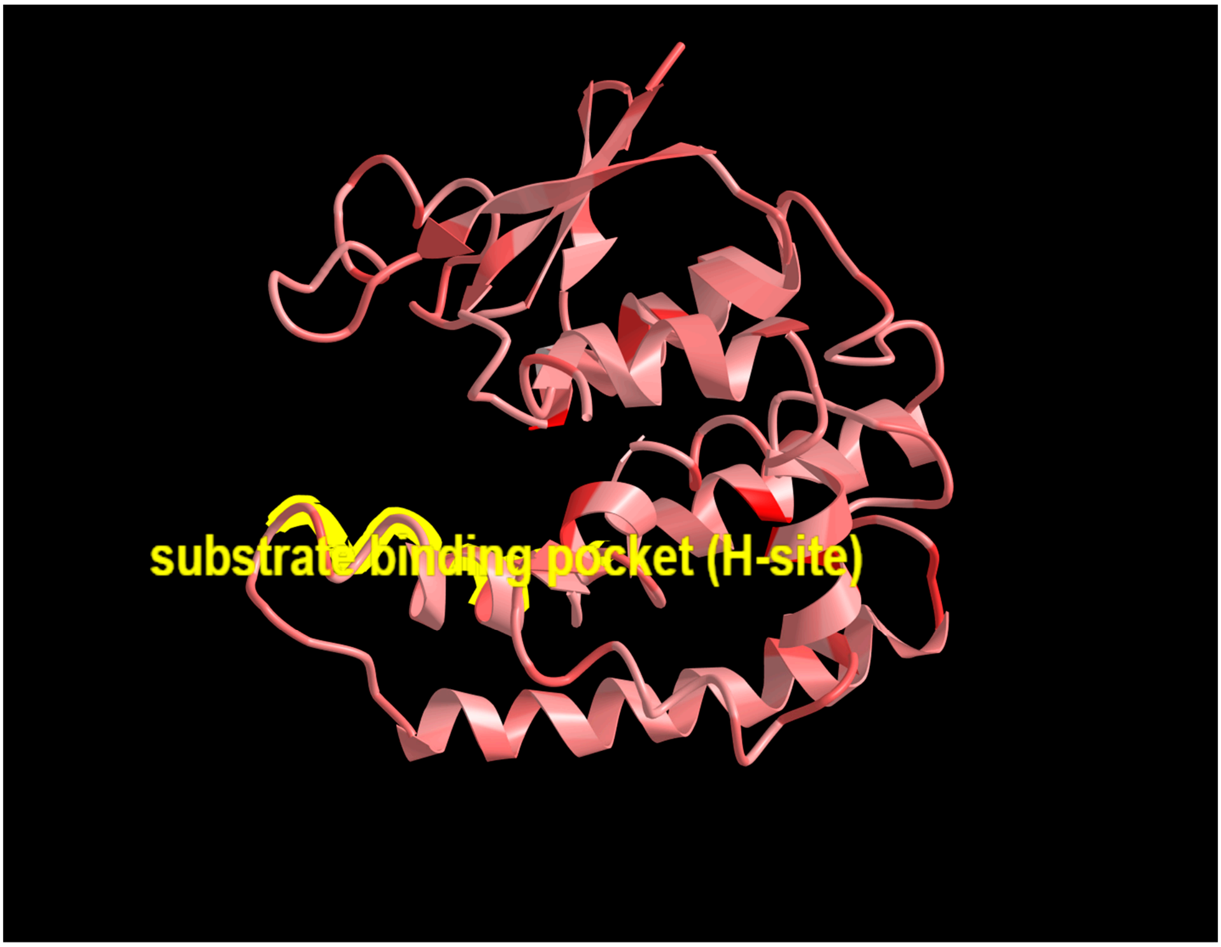

Figure 19: 3D homology modelling of resistant GST gene Resistant 19. The Substrate binding pocket (H-site) is highlighted in yellow

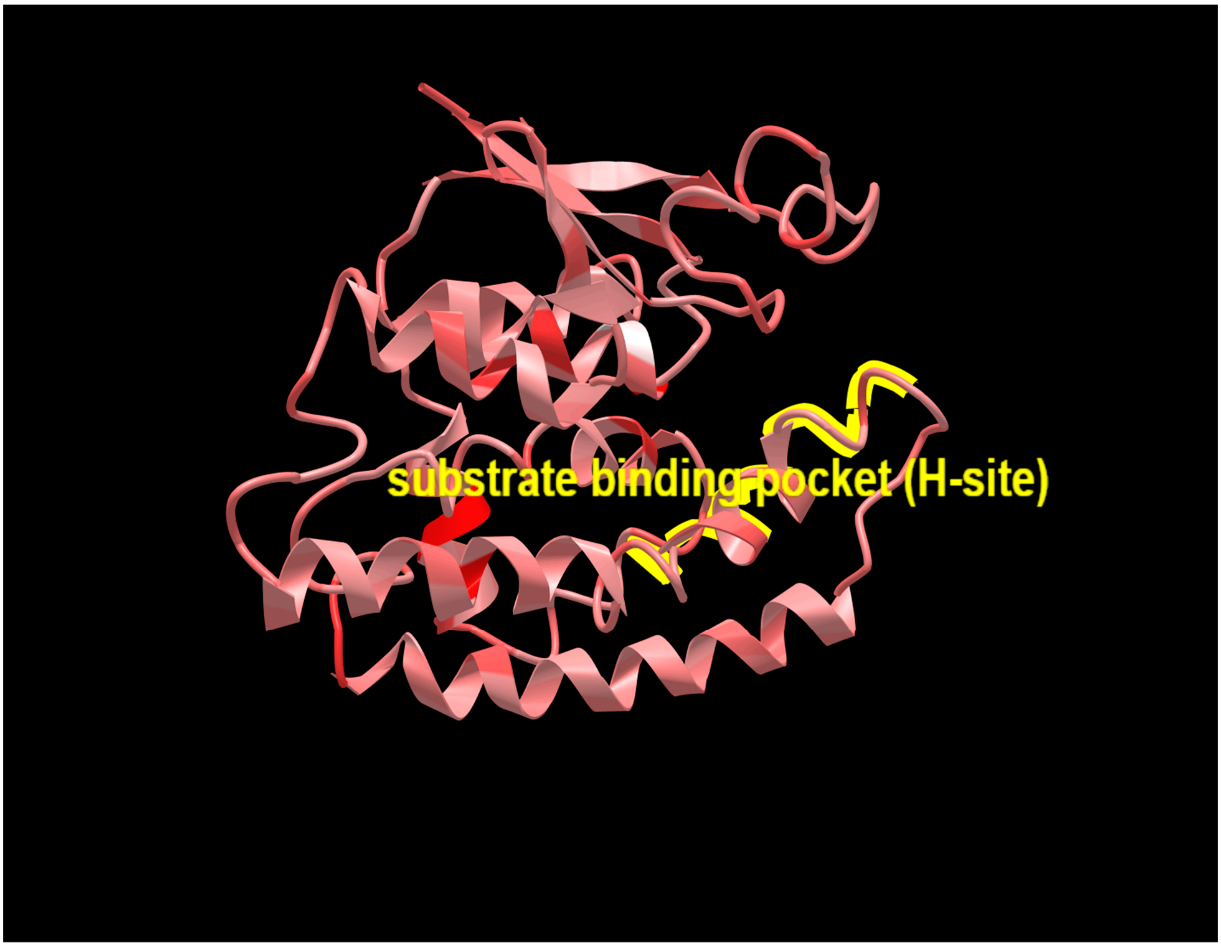

Figure 20: 3D homology modelling of resistant GST gene Resistant 20. The Substrate binding pocket (H-site) is highlighted in yellow

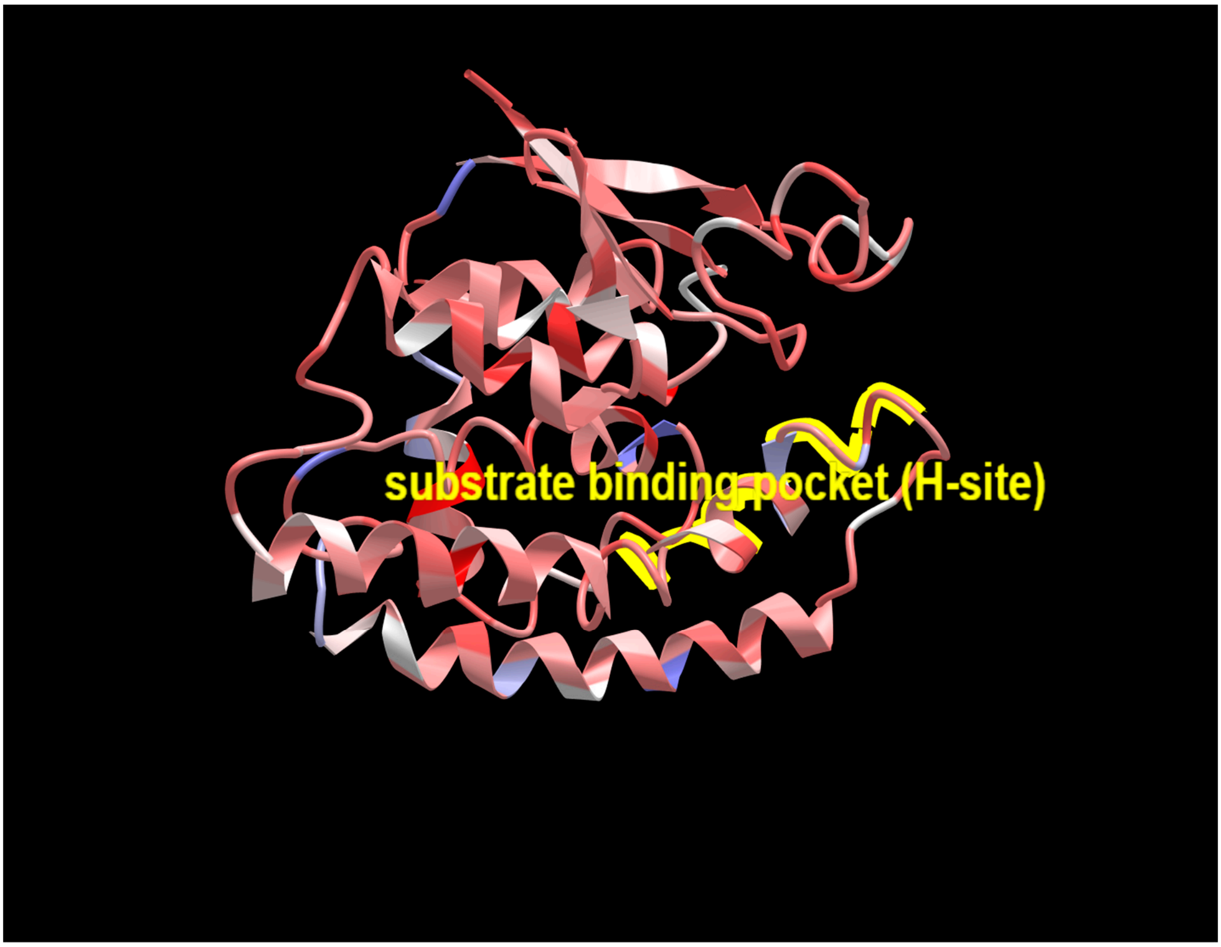

Figure 21: 3D homology modelling of resistant GST gene Resistant 21. The Substrate binding pocket (H-site) is highlighted in yellow

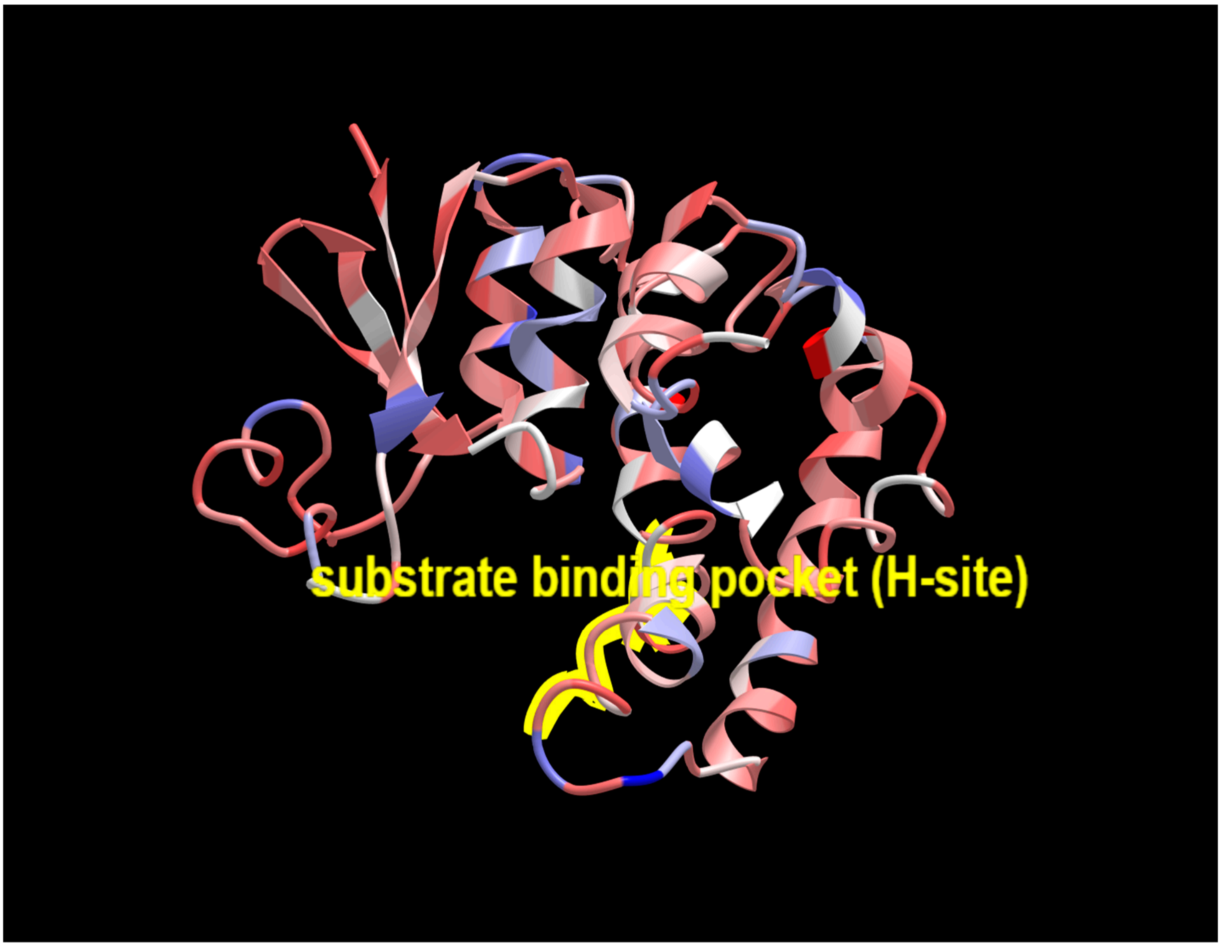

Figure 22: 3D homology modelling of resistant GST gene Resistant 22. The Substrate binding pocket (H-site) is highlighted in yellow
