# Supplementary material for: Genome-Wide Evolutionary Analysis of Putative Non-Specific Herbicide Resistance Genes and Compilation of Core Promoters between Monocots and Dicots
Source: Genes (Basel). 2022 Jun 29;13(7):1171. doi: 10.3390/genes13071171 (PMC9316059; doi:10.3390/genes13071171)
Supplement: Supplementary file 1 [file genes-13-01171-s001.zip › Supplementary file S8.pdf]

Supplementary file S8: Sequence showing the GSH binding site (G-site) and substrate binding pocket site (H-site) in the resistant GST sequence

>Resistant1

MASSQEEVTLLGVVGSPFLHRVQIALKLKGVEYKYLEDDLNNKSDLLLKYNPVYKMIPVLVHNEKPISESLVIVEYIDDTW
KNNPILPSDPYQRALARFWAKFIDDKCVVPAWKSAFMTDEKEKEKAKEELFEALSFLENELKGKFFGGEEFGFVDIAAVLI
PIIQEIAGLQLFTSEKFPKLSKWSQDFHNHPVVNEVMPPKDQLFAYFKARAQSFVAKRKN

>Resistant2

MSKSEDLKLLGGWFSPFALRVQIALNLKGLEYEVVEETLNPKSDLLLKSNPVHKKIPVFFHGDKVICESAIIVEYIDEAWTN
VPSILPQNAYDRANARFWFAYIDEKWFTSLRSVLVAEDDEAKKPHFEQAEEGLERLEEVFNKYSEGKAYFGGDSIGFIDI
GFGSFLSWMRVIEEMSGRKLLDEKKHPGLTQWAETFAADPAVKGILPETDKLVEFAKILQLKWTAAAAAAK

>Resistant3

MAEQDKVILHGMWASPYAKRVELALNFKGIPYEYVEEDLRNKSDLLLKYNPVHKKVPVLVHNGKAIAESMVILEYIDET
WKDGPKLLPSDSYKRAQARFWCHFIQDQLMESTFLVVKTDGEAQQKAIDHVYEKLKVLEDGMKTYLGEGNAIISGVEN
NFGILDIVFCALYGAYKAHEEVIGLKFIVPEKFPVLFSWLMAIAEVEAVKIATPPHEKTVGILQLFRLSALKSSSATE

>Resistant4

ETFGSKSELLLKSNPVYKKIPVMIHGDKPISESMIIVQYIDDVWSSAGHSIIPSDPYDASIARFWATYIDDKFFPSLFAIAKSK
DEEERKAAIEQVIAAFGILEEAYQKTSKGKDFFGGEKIGYIDISFGCYVGWIKASEKMNGIKLFDETKVPGLTKWAEKSVP
DESV

>Resistant5

ESLAHKSPLLLEMNPVHKKVPVLIHNGKPVSESNIIVQYIDDTWQNSSPPLLPSDPYLKAQARFWADFIDNKVRFSGIWR
TKGEEQERAKKEFVDLLKVLEGQLGDKPYLVGESFGYVDIMLIPFSGYFYALETIGKMDIE

>Resistant6

MANEVILLDFWPSMFGMRTRIALREKGVEFEYREEDLRNKSPLLLQMNPIHKKIPVLIHNGKPVNESIIQVQYIDEVWSH
KNPILPSDPYLRAQARFWADFIDKKLYDAQRKVWATKGEEQEAGKKDFIEILKTLESELGDKPYFSGDDFGYVDIALIGFY
TWFPAYEKFANFSIESEVPKLIAWVKKCLQRESVAKSLPDPEKVTEFVSELRKKFVPE

>Resistant7

MAGGDDLKLLGAWPSPFVTRVKLALALKGLSYEDVEEDLYKKSELLLKSNPVHKKIPVLIHNGAPVCESMIILQYIDEVFA
STGPSLLPADPYERAIARFWVAYVDDKLVAPWRQWLRGKTEEEKSEGKKQAFAAVGVLEGALRECSKGGGFFGGDGV
GLVDVALGGVLSWMKVTEALSGDKIFDAAKTPLLAAWVERFIELDAAKAALPDVGRLLEFAKAREAAAAASK

>Resistant8

MAGGDDLKLLGTWPSPYAIRVKLALAHKGLSYEYAEEDLANTSELLLSSNPVHKKIPALIHNGVAVCESNIIVEYIDEAFAG
PSILPADPYERAIARFWAAYVDDKLFGAWATMLFSGKTEEEKLEGKNALFAALETLEGALAECSDGKDFFGGHTVGLVD
MALGSHLSWLKATEVMAGEEILRSDRTQLLAAWMARFSELYAAKAALPDVDRMAKMRQERLAAAAAA

>Resistant9

MAGGDDLKLLGTWPSPYAIRVKLALAHKGLSYEYAEEDLANKSELLLSSNPVHRKIPVLIHNGVPVCESNIILEYIDEAFAG
PSILPADPYERAMARFWAAYVDDKLLAAWATMVFKGKTEEEKLEGKKALFAALETLEGALAKCSDGKDFFGGDTVGLV
DMVLGSHLSFLKATEAMAGEEILRSDRTQLLAAWMARFSELDAAKAALPDVDRVVEFAKMRQARLAAAAAAASNN

>Resistant10

MAGGNDLKLLGTWPSPYAIRVKLALAHKGLSYEYAEEDLANKSELLLSSNPVHRKIPVLIHNGVPVCESNIILEYIDEAFAG
RSILPADPYERAMARFWAAYVDDKLLAAWATMVFKGKTEEEKLEGKKALFAALETLEGALAKCSDGKDFFGGDTVGLV
DMVLGSHLSFLKATEAMAGEEILRSDRTQLLAAWMARFSELDAAKAALPDVDRVVEFVKMRQARLAAAAAAASNN

>Resistant11

MAPVKVFGPAMSTNVARVLVFLEEVGADYEVVDMDFKVMEHKSPEHLARNPFGQIPAFQDGDLLLFESRAISKYVLRK
YKTGEVDLLREGNLKEAAMVDVWTEVDAHTYNPALSPIVYQCLFNPMMRGIPTDEKVVAESLEKLKKVLEVYEARLSQ
HEYLAGDFVSFADLNHFPYTFYFMATPHAALFGSYPHVKAWWERIMARPAIKKISATMVPPKA

>Resistant12

MAGIKVFGHPASIATRRVLIALHEKNLDFELVHVELKDGEHKKEPFLSRNPFGQVPAFEDGDLKLFESRAITQYIAHRYEN
QGTNLLQTDSKNISQYAIMAIGMQVEDHQFDPVASKLAFEQIFKSIYGLTTDEAVVAEEEAKLAKVLDVYEARLKEFKYL
AGETFTLTDLHHIPAIQYLLGTPTKKLFTERPRVNEWVAEITKRPASEKVQ

>Resistant13

MAGGDDLKLLGAWPSPFVTRVKLALALKGLSYEDVEEDLYKKSELLLKSNPVHKKIPVLIHNGAPVCESMIILQYIDEVFA
STGPSLLPADPYERAIARFWVAYVDDKLVAPWRQWLRGKTEEEKSEGKKQAFAAVGVLEGALRECSKGGGFFGGDGV
GLVDVALGGVLSWMKVTEALSGDKIFDAAKTPLLAAWVERFIELDAAKAALPDVGRLLEFAKAREAAAAASK

>Resistant14

MAGGDDLKLLGAWPSPFVTRVKLALALKGLSYEDVEEDLYKKSELLLKSNPVHKKIPVLIHNGAPVCESMIILQYIDEVFA
STGPSLLPADPYERAIARFWVAYVDDKLVAPWRQWLRGKTEEEKSEGKKQAFAAVGVLEGALRECSKGGGFFGGDGV
GLVDVALGGVLSWMKVTEALSGDKIFDAAKTPLLAAWVERFIELDAAKAALPDVGRLLEFAKAREAAAAASK

>Resistant15

MAGGDDLKLLGAWPSPFVTRVKLALALKGLSYEDVEEDLYKKSELLLKSNPVHKKIPVLIHNGAPVCESMIILQYIDEVFA
STGPSLLPADPYERAIARFWVAYVDDKLVAPWRQWLRGKTEEEKSEGKKQAFAAVGVLEGALRECSKGGGFFGGDGV
GLVDVALGGVLSWMKVTEALSGDKIFDAAKTPLLAAWVERFIELDAAKAALPDVGRLLEFAKAREAAAAASK

>Resistant16

MAPMKLYGAVMSWNLTRCATALEEAGSDYEIVPINFATAEHKSPEHLVRNPFGQVPALQDGDLYLFESRA
ICKYAARKNKPELLREGNLEEAAMVDVWIEVEANQYTAALNPILFQVLISPMLGGTTDQKVVDENLEKLKKVLEVYEARL
TKCKYLAGDFLSLADLNHVSVTLCLFATPYASVLDAYPHVKAWWSGLMERPSVQKVAALMKPSA

>Resistant17

MATPAVKVYGWAISPFVSRALLALEEAGVDYELVPMSRQDGDHRRPEHLARNPFGKVPVLEDGDLTLFESRAIARHVL
RKHKPELLGGGRLEQTAMVDVWLEVEAHQLSPPAIAIVVECVFAPFLGRERNQAVVDENVEKLKKVLEVYEARLATCTY
LAGDFLSLADLSPFTIMHCLMATEYAALVHALPHVSAWWQGLAARPAANKVAQFMPVGAGAPKEQE

>Resistant18

MAPVKVFGPAMSTNVARVLVCLEEVGAEYEVVDIDFKAMEHKSPEHLVRNPFGQIPAFQDGDLLLFESRAIAKYVLRKY
KTDEVDLLREGNLKEAAMVDVWTEVDAHTYNPALSPIVYECLINPLMRGLPTNQTVVDESLEKLKKVLEVYEARLSQHK
YLAGDFVSFADLNHFPYTFYFMATPHAALFDSYPHVKAWWESLMARPAIKKLAAQMVPKKP

>Resistant19

MAPMKLYGAVMSWNVTRCATALEEAGSDYEIVPINFATAEHKSPEHLVRNPFGQVPALQDGDLYLFESRAICKYAARK
NKPELLREGNLEEAAMVDVWIEVEANQYTAALNPILFQVLISPMLGGTTDQKVVDENLEKLKKVLEVYEARLTKCKYLA
GDFLSLADLNHVSVTLCLFATPYASVLDAYPHVKAWWSGLMERPSVQKVAALMKPSA

>Resistant20

MAPMKLYGAVMSWNVTRCATALEEAGSDYEIVPINFATAEHKSPEHLVRNPFGQVPALQDGDLYLFESRAICKYAARK
NKPELLREGNLEEAAMVDVWIEVEANQYTAALNPILFQVLISPMLGGTTDQKVVDENLEKLKKVLEVYEARLTKCKYLA
GDFLSLADLNHVSVTLCLFATPYASVLDAYPHVKAWWSGLMERPSVQKVAALMKPSA

>Resistant21

MAPVKLYGATLSWNVTRCVAALEEAGVEYELVPINFGTGEHKSPDHLARNPFGQVPALQDGDLYVFESRAICKYACRK
NKPELLKEGDIKESAMVDVWLEVEAHQYTAALSPILFECLIHPMLGGATDQKVIDDNLVKIKNVLAVYEAHLSKSKYLAG
DSLSLADLNHVSVTLCLAATPYASLFDAYPHVKAWWTDLLARPSVQKVAALMKP

>Resistant22

MTPVKVFGPAQSTNVARVLLCLEEVGAEYEVVNVDFTVMEHKSPEHLKRNPFGQIPAFQDGDLYLFESRAIGKYILRKYK
TREADLLREGNLREAAMVDVWTEVETHQYNSAISPIVYECIINPAMRGIPTNQKVVDESAEKLKKVLEVYEARLSQSTYL
AGDFVSFADLNHFPYTFYFMGTPYASLFDSYPHVKAWWERLMARPSVKKLAAVMAPQGA
