# Supplementary material for: Genome-Wide Evolutionary Analysis of Putative Non-Specific Herbicide Resistance Genes and Compilation of Core Promoters between Monocots and Dicots
Source: Genes (Basel). 2022 Jun 29;13(7):1171. doi: 10.3390/genes13071171 (PMC9316059; doi:10.3390/genes13071171)
Supplement: Supplementary file 1 [file genes-13-01171-s001.zip › Table S1.pdf]

Table S1: Template and target protein used for homology modelling

Gene Id	Gene	Template	Species
**Resistant 1**	GST	6GHF	Synthetic construct
**Resistant 2**	GST	5KEJ	*Mangifera indica*
**Resistant 3**	GST	4J2F	Ricinus communis
**Resistant 4**	GST	5KEJ	*Mangifera indica*
**Resistant 5**	GST	5G5A	*Arabidopsis thaliana*
**Resistant 6**	GST	5G5A	*Arabidopsis thaliana*
**Resistant 7**	GST	1GWC	*Aegilops tauschii*
**Resistant 8**	GST	1GWC	*Aegilops tauschii*
**Resistant 9**	GST	1GWC	*Aegilops tauschii*
**Resistant 10**	GST	1GWC	*Aegilops tauschii*
**Resistant 11**	GST	6RIV	*Alopecurus myosuroides*
**Resistant 12**	GST	1GNW	*Arabidopsis thaliana*
**Resistant 13**	GST	1GWC	*Aegilops tauschii*
**Resistant 14**	GST	1GWC	*Aegilops tauschii*
**Resistant 15**	GST	1GWC	*Aegilops tauschii*
**Resistant 16**	GST	1BYE	*Zea mays*
**Resistant 17**	GST	1BYE	*Zea mays*
**Resistant 18**	GST	6RIV	*Alopecurus myosuroides*
**Resistant 19**	GST	1BYE	*Zea mays*
**Resistant 20**	GST	1BYE	*Zea mays*
**Resistant 21**	GST	1BYE	*Zea mays*
**Resistant 22**	GST	6RIV	*Alopecurus myosuroides*
**R1**	CYP450	5T6Q	*Oryctolagus cuniculus*
**R2**	CYP450	6VBY	*Sorghum bicolor*
**R3**	CYP450	5YLW	*Salvia miltiorrhiza*
**R4**	CYP450	1TQN	*Homo sapiens*
**R5**	CYP450	5YLW	*Salvia miltiorrhiza*
**R6**	CYP450	5YLW	*Salvia miltiorrhiza*
**R7**	CYP450	5YLW	*Salvia miltiorrhiza*
**R8**	CYP450	5YLW	*Salvia miltiorrhiza*
**R9**	CYP450	5YLW	*Salvia miltiorrhiza*
**R10**	CYP450	6VBY	*Sorghum bicolor*
**R11**	CYP450	1TQN	*Homo sapiens*
**R12**	CYP450	6VBY	*Sorghum bicolor*
