# Supplementary material for: Genome-Wide Evolutionary Analysis of Putative Non-Specific Herbicide Resistance Genes and Compilation of Core Promoters between Monocots and Dicots
Source: Genes (Basel). 2022 Jun 29;13(7):1171. doi: 10.3390/genes13071171 (PMC9316059; doi:10.3390/genes13071171)
Supplement: Supplementary file 1 [file genes-13-01171-s001.zip › Table S2.pdf]

Table S2: List of resistant CYP450 and GST used for this study

Gene Id	Gene class	Species	Resistant against herbicide	Reference
**Resistant1**	GST 11	*Glycine max*	fomesafen and acifluorfen	Patent: US 7056715-A
**Resistant2**	GST 5	*Glycine max*	fomesafen and acifluorfen	Patent: US 7056715-A
**Resistant3**	GST 12	*Glycine max*	fomesafen and acifluorfen	Patent: US 7056715-A
**Resistant4**	GST 17	*Papaver rhoeas*	quinclorac	Rouse, 2017
**Resistant5**	GST 19	*Arabidopsis thaliana*	1-chloro-2,4-dinitrobenzene and chloroacetanilide	DeRidder et al. 2002
**Resistant6**	GST 19	*Arabidopsis thaliana*	1-chloro-2,4-dinitrobenzene and chloroacetanilide	DeRidder et al. 2002
**Resistant7**	GST 6	*Aegilops tauschii*	dimethenamid	Riechers et al. 1997
**Resistant8**	GST 6	*Alopecurus myosuroides*	Chlorotoluron, fenoxaprop-p-ethyl	Cummins et al. 2013
**Resistant9**	GST 6	*Alopecurus myosuroides*	Chlorotoluron, fenoxaprop-p-ethyl	Cummins et al. 2013
**Resistant10**	GST 6	*Alopecurus myosuroides*	Chlorotoluron, fenoxaprop-p-ethyl	Cummins et al. 2013
**Resistant11**	GSTF1	*Lolium rigidum*	Chlorotoluron, fenoxaprop-p-ethyl	Cummins et al. 2013
**Resistant12**	GSTF2	*Arabidopsis thaliana*	fenchlorazole-ethyl	Thom et al. 2002
**Resistant13**	GST 6	*Aegilops tauschii*	dimethenamid	Riechers et al. 1997
**Resistant14**	GST 6	*Aegilops tauschii*	dimethenamid	Riechers et al. 1997
**Resistant15**	GST 6	*Aegilops tauschii*	dimethenamid	Riechers et al. 1997
**Resistant16**	GSTF1	*Zea mays*	alachlor and atrazine	Shah et al. 1986
**Resistant17**	GSTF4	*Zea mays*	benoxacor	Irzyk et al. 1995
**Resistant18**	GSTF1	*Hordeum vulgare*	1-chloro-2,4-dinitrobenzene, fenoxaprop	Scalla and Roulet, 2002
**Resistant19**	GSTF1	*Zea mays*	alachlor and atrazine	Shah et al. 1986
**Resistant20**	GSTF1	*Zea mays*	alachlor and atrazine	Shah et al. 1986
**Resistant21**	GSTF1	*Triticum aestivum*	fenoxaprop-ethyl	Goetzberger et al. 2000

**Resistant22**	GSTF1	*Oryza sativa*	fenoxaprop-ethyl	Patent: WO WO03000898
**R1**	CYP72A15	*Lolium rigidum*	Glufosinate-ammonium, quinclorac, 2,4-dichlorophenoxyacetic acid (2,4-D)	Fischer et al. 2001
**R2**	CYP81A10v7	*Lolium rigidum*	diclofop-methyl, tralkoxydim, chlorsulfuron	Han et al. 2021
**R3**	CYP76C1	*Arabidopsis thaliana*	Phenylurea herbicides	Hofer et al. 2014
**R4**	CYP72A31	Oryza sativa	Bispyribac sodium	Saika et al. 2014
**R5**	CYP76B1	*Helianthus tuberosus*	7-ethoxycoumarin *O*-de-ethylase	Batard et al. 1998
**R6**	CYP71A10	*Glycine max*	Phenylurea herbicides	Siminszky et al. 1999
**R7**	CYP81B2	*Nicotiana tabacum*	Chlortoluron, 2,4-dichlorophenoxyacetic acid (2,4-D)	Yamada et al. 2000
**R8**	CYP71A11	*Nicotiana tabacum*	Chlortoluron, 2,4-dichlorophenoxyacetic acid (2,4-D)	Yamada et al. 2000
**R9**	CYP81A6	*Arabidopsis thaliana*	bentazon and sulfonylurea herbicides	Liu et al. 2012
**R10**	CYP71AK2	*Echinochloa phyllopogon*	bispyribac-sodium	Iwakami et al. 2014
**R11**	CYP72A254	*Echinochloa phyllopogon*	bispyribac-sodium	Iwakami et al. 2014
**R12**	CYP81A6	*Oryza sativa*	bentazon and sulfonylurea herbicides	Pan et al. 2006
