# Supplementary material for: Genome-Wide Evolutionary Analysis of Putative Non-Specific Herbicide Resistance Genes and Compilation of Core Promoters between Monocots and Dicots
Source: Genes (Basel). 2022 Jun 29;13(7):1171. doi: 10.3390/genes13071171 (PMC9316059; doi:10.3390/genes13071171)
Supplement: Supplementary file 1 [file genes-13-01171-s001.zip › Table S3.pdf]

Table S3: Information of the differential expression study used for this study

Expt. ID	Plant	Accession number	Herbicide	Rates applied (g/ha)	Active ingredient	Mode of action	Citation
**AT-00531, AT-00532, AT-00533**	*Arabidopsis thaliana* (accession Col-0)	GSE8912, GSE8913, GSE8925, GSE8926 GSE8927	Arsenal™[a]	0.395	Imazapyr	ALS inhibitor	Das et al. 2010
**AT-00531, AT-00532, AT-00533**	*Arabidopsis thaliana* (accession Col-0)	GSE8912, GSE8913, GSE8925, GSE8926 GSE8927	Beacon™	0.586	Primisulfuron-methyl	ALS inhibitor	Das et al. 2010
**AT-00531, AT-00532, AT-00533**	*Arabidopsis thaliana* (accession Col-0)	GSE8912, GSE8913, GSE8925, GSE8926 GSE8927	FirstRate™	0.163	Cloransulam-methyl	ALS inhibitor	Das et al. 2010
**AT-00531, AT-00532, AT-00533**	*Arabidopsis thaliana* (accession Col-0)	GSE8912, GSE8913, GSE8925, GSE8926 GSE8927	Oust™	0.131	Sulfometuron methyl	ALS inhibitor	Das et al. 2010
**AT-00531, AT-00532, AT-00533**	*Arabidopsis thaliana* (accession Col-0)	GSE8912, GSE8913, GSE8925, GSE8926 GSE8927	Roundup original™	10.652	Glyphosate	EPSPS inhibitor	Das et al. 2010
**AT-00459**	*Arabidopsis thaliana* (accession Col-0)	GSE28431		200	Fenclorim	Enhances the metabolism of pretilachlor in rice	Skipsey et al. 2011
**AT-00475**	*Arabidopsis thaliana* (accession Col-0)	GSE24052	Cadence	70	Dicamba	Synthetic auxin	Gleason et al. 2011
**AT-00581**	*Arabidopsis thaliana*	GSE25849		1500	Mefenpyr-diethyl	Safener	Behringer et al. 2011

	[Salicylic acid-insensitive-1; (sail-1) ecotype Nossen (No)Shah et al. 1997]; [tga2tga3tga5tga6 (tga2/3/5/6; Kesarwani et al. 2007]; [sid2-2 (Nawrath and Metraux 1999)]					
**AT-00581**	*Arabidopsis thaliana [Salicylic acid-insensitive-1; (sail-1) ecotype Nossen (No)Shah et al. 1997];* [tga2tga3tga5tga6 (tga2/3/5/6; Kesarwani et al. 2007]; [sid2-2 (Nawrath and Metraux 1999)]	GSE25849	1500	Isoxadifen-ethyl	Safener	Behringer et al. 2011
**AT-00838**	*Arabidopsis thaliana* (accession Col-0)	GSE147962	110	Paraquat	Photosynthesis inhibitor	Zandalinas et al. 2021
